# Selective targeting of TBXT with DARPins identifies regulatory networks and therapeutic vulnerabilities in chordoma

**DOI:** 10.1126/sciadv.adu2796

**Published:** 2025-09-03

**Authors:** Charles S. Umbaugh, Marie Groth, Cihan Erkut, Kwang-Seok Lee, Joana Marinho, Simon Linder, Florian Iser, Jonas N. Kapp, Petra Schroeter, Simay Dolaner, Asli Kayserili, Dominic Helm, Martin Schneider, Julia Hartmann, Philipp Walch, Thomas F. E. Barth, Kevin Mellert, Birgit Dreier, Jonas V. Schaefer, Andreas Plückthun, Stefan Fröhling, Claudia Scholl

**Affiliations:** ^1^ Division of Applied Functional Genomics, German Cancer Research Center (DKFZ), Heidelberg, Germany.; ^2^ National Center for Tumor Diseases (NCT), NCT Heidelberg, a partnership between DKFZ and Heidelberg University Hospital, Heidelberg, Germany.; ^3^ Division of Translational Medical Oncology, DKFZ, Heidelberg, Germany.; ^4^ Faculty of Biosciences, Heidelberg University, Heidelberg, Germany.; ^5^ German Cancer Consortium (DKTK), Heidelberg, Germany.; ^6^ Department of Biochemistry, University of Zurich, Zurich, Switzerland.; ^7^ Proteomics Core Facility, DKFZ, Heidelberg, Germany.; ^8^ Institute of Pathology, Ulm University, Ulm, Germany.; ^9^ Institute of Human Genetics, Heidelberg University, Heidelberg, Germany.

## Abstract

The embryonic transcription factor TBXT (brachyury) drives chordoma, a spinal neoplasm without effective drug therapies. TBXT’s regulatory network is poorly understood, and strategies to disrupt its activity for therapeutic purposes are lacking. We developed designed ankyrin repeat proteins that block TBXT-DNA binding (T-DARPins). In chordoma cells, T-DARPins reduced cell cycle progression, spheroid formation, and tumor growth in mice and induced signs of senescence and differentiation. Transcriptomic and proteomic analyses identified gene networks involved in cell cycle regulation, embryonic cell identity, and interferon response and revealed features of regulome components, such as susceptibility to pharmacologic inhibition and the fine-tuning of TBXT downstream effectors through IGFBP3. Finally, we found high interferon signaling in chordoma cell lines and patient tumors, which was promoted by TBXT and associated with sensitivity to JAK2 inhibitors. These findings demonstrate the potential of DARPins for probing nuclear proteins to understand the regulatory networks of transcription factor–driven cancers, including entry points for therapies that warrant testing in patients.

## Teaser

Designed ankyrin repeat proteins targeting the embryonic transcription factor TBXT provide insights into chordoma biology.

## INTRODUCTION

Chordoma is a rare cancer that typically arises at the sacrum, mobile spine, or skull base, likely from remnants of the notochord, and accounts for up to 4% of primary bone tumors and 20% of primary spine tumors (*1*, *2*). Clinical characteristics include locally aggressive growth, a tendency to recur, the potential for metastasis, and resistance to conventional chemotherapy (*3*). First-line treatment is usually surgery with the goal of complete resection, followed by adjuvant radiotherapy. However, local control is rarely achieved because most chordomas are adjacent to vital structures, particularly at the skull base (*4*). As local therapies are exhausted in most patients as the disease progresses and chordomas are typically resistant to conventional chemotherapy (*4*), novel approaches to systemic treatment of advanced chordoma are urgently needed.

A potential therapeutic target is the *TBXT* gene (also known as brachyury), which is amplified in 7%, duplicated in 27%, and overexpressed in more than 90% of cases (*5*, *6*) and has been identified as the key driver of chordoma development and progression (*5*, *7*, *8*). It encodes a T-box family transcription factor that regulates mesoderm and notochord formation and axial skeleton development during embryogenesis but is not expressed in most adult tissues (*9*). TBXT exerts transcriptional activity by binding as a homodimer to T-box element-containing DNA in the promoter or enhancer regions of target genes (*10*, *11*). TBXT itself is expressed under the control of a superenhancer, regulates its own expression, and can control other genes such as *SOX9*, *SAE1*, *TPX2*, and *ATP6V1B2* through direct binding to other superenhancers (*7*).

Preclinical studies have identified several potential targets for chordoma drug treatment, e.g., mTOR, PI3K, PDGFR, VEGFR, and EGFR (*4*, *12*), some of which have been evaluated in single-agent clinical trials. However, the clinical efficacy of these approaches has thus far been limited to stable disease and occasional partial responses (*4*, *13*). Other therapeutic strategies include TBXT-focused vaccines or immunotherapies, which have led to modest improvements in symptoms or progression-free survival, respectively (*4*). Nevertheless, it is hoped that increased response rates may be achieved through combination therapies, potentially incorporating future TBXT-targeting agents. Preclinical efforts to target TBXT directly or indirectly have explored blocking the enhancer-associated components CDK7/12/13 (*7*, *8*); inhibiting the receptor tyrosine kinase EGFR with afatinib, which results in TBXT suppression (*14*); and degradation via bifunctional TBXT-binding nucleic acids that recruit an E3 ligase (*15*). Direct pharmacologic inhibition of TBXT is complicated by the fundamental architecture of transcription factors, which impedes direct binding of small molecules to functionally important domains. However, a novel derivative of the EGFR inhibitor afatinib, DHC-156 (*16*), and the identification of TBXT binders with low micromolar potency (*17*) offer initial proof of principle for the feasibility of direct TBXT inhibition.

A novel class of molecules with the potential to inhibit “undruggable” proteins are designed ankyrin repeat proteins (DARPins), consisting of a variable number of internal repeats with a randomized surface that mediates tight and specific binding to the target protein, flanked by capping repeats with a hydrophilic surface (*18*). They functionally resemble monoclonal antibodies at only about 10% of the molecular mass, are readily produced in routine bacterial cultures, and can be fused to generate multivalent proteins that bind multiple epitopes or adjacent target proteins (*18*). DARPins have demonstrated their potential as specific and effective therapeutics targeting extracellular proteins. For example, abicipar pegol inhibits the function of VEGF as effectively as an established Fab fragment of a VEGF-directed monoclonal antibody but with potentially longer duration (*19*). In contrast to antibodies, DARPins fold in the cytoplasm and can thus be developed for intracellular applications, where tightly regulated expression is achieved by delivering DARPin-encoding DNA via viral vectors with inducible and/or tissue-specific promoters (*18*, *20*). A recent example is the inhibition of KRAS isoforms that drive oncogenic signaling in the cytoplasm of cancer cells (*21*). However, it has not yet been investigated whether DARPins can also block the function of nuclear proteins, whose deregulation underlies many hematologic and solid-organ malignancies.

In this study, we developed and preclinically characterized DARPins that bind TBXT, thereby blocking its interaction with DNA. Furthermore, we tested these compounds in chordoma cells to determine their utility for direct targeting of an essential transcription factor, explore the phenotypic and transcriptional consequences of TBXT inhibition, and identify components of the gene network regulated by TBXT that could be new entry points for therapeutic intervention.

## RESULTS

### Development of DARPins that block the TBXT-DNA interaction

**Fig. 1.**
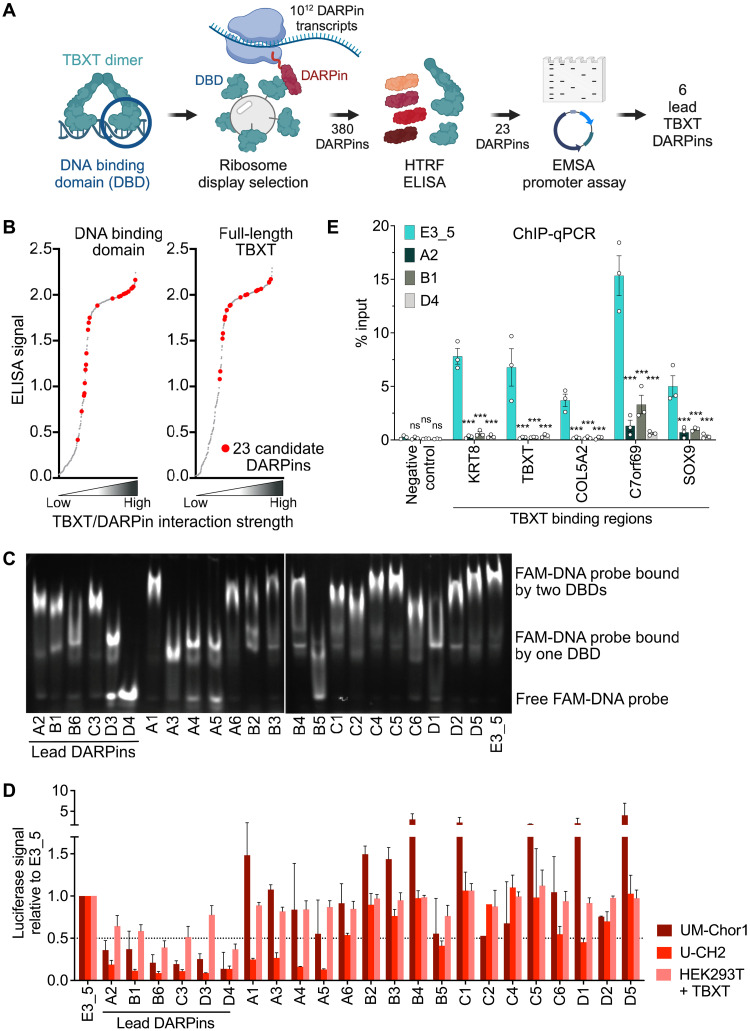
Generation of T-DARPins. (**A**) Schematic of the screening, selection, and testing of DARPins binding the TBXT DBD. Created in BioRender. S.F. (2024) BioRender.com/l53i374. (**B**) ELISA signal intensities (*y* axis) with purified full-length TBXT (right) and the DBD (left) against crude bacterial extracts of the 380 DARPins retrieved by ribosome display selection, ordered by signal strength (*x* axis). The 23 candidate DARPins are shown in red. (**C**) EMSA with a 6-FAM–labeled DNA probe containing two palindromic TBXT binding motifs, recombinant TBXT DBD, and the 23 candidate DARPins or E3_5. (**D**) Dual-luciferase assay with a TBXT-responsive reporter vector in UM-Chor1, U-CH2, and HEK293T cells stably expressing HA-tagged TBXT and lentivirally transduced with 23 candidate TBXT DARPins or E3_5. The dotted line indicates a 50% luciferase signal relative to control. Mean + SEM of two to five biological replicates: UM-Chor1 and U-CH2 with E3_5, A2, B1, B6, C3, or D4, *n* = 5; UM-Chor1 and U-CH2 with the remaining DARPins, *n* = 2; TBXT-expressing HEK293 with C2 and D5, *n* = 2; TBXT-expressing HEK293 with the remaining DARPins, *n* = 3. (**E**) ChIP-qPCR analysis of TBXT chromatin occupancy in UM-Chor1-iDARPin cells expressing either DARPin E3_5 or T-DARPins A2, B1, or D4 for 24 hours. TBXT enrichment was assessed by qPCR at a negative control region (*KLK3*) and five established TBXT binding sites (*KRT8*, *TBXT*, *COL5A2*, *C7orf69*, and *SOX9*). Two-way ANOVA with Dunnett’s test for multiple comparisons versus negative control E3_5; mean ± SEM of three biological replicates. ns, not significant, ***P ≤ 0.001.

To generate DARPins that prevent TBXT from binding to DNA, we performed ribosome display selection with a library of ~10^12^ DARPins against the DNA binding domain (DBD) of TBXT, which yielded 380 candidates with varying specificity and affinity (Fig. 1A). These were further narrowed down to 23 potential TBXT-targeting DARPins based on monomeric behavior in size-exclusion chromatography, signal intensities in enzyme-linked immunosorbent assays (ELISAs), and homogeneous time-resolved fluorescence (HTRF) using biotinylated DBD and full-length TBXT proteins (Fig. 1, A and B, and fig. S1, A to C). The selection criteria included binding to both the DBD and full-length TBXT, as well as high but varying HTRF signals, which potentially enhanced the likelihood of identifying DARPins with different binding geometries that could inhibit TBXT through different mechanisms.

To directly measure the capability of the 23 DARPins to disrupt TBXT binding to DNA, we performed electrophoretic mobility shift assays (EMSAs) using a 6-carboxyfluorescein (6-FAM)–labeled DNA probe containing two palindromic TBXT binding sites and recombinant DBD protein. DNA bound by two TBXT DBDs is less mobile during gel electrophoresis and thus shifted to the top. In contrast, unbound DNA or DNA partially occupied by a TBXT DBD moves faster through the gel and is detected in the middle or at the bottom, respectively (Fig. 1C). We observed DARPins that largely failed to disrupt DBD-DNA complexes (e.g., A1, C4, C5, and D5) and behaved like the nontargeting control DARPin E3_5 (*22*). We also found DARPins with a moderate ability to disrupt the interaction between the DBD and one or two TBXT binding sites (e.g., A2, B1, B6, C3, D3, A3, A4, A5, and D1), and one DARPin (D4) that caused complete displacement of the DNA probe (Fig. 1C).

Next, we measured the candidate DARPins’ capacity to inhibit TBXT transcriptional activity. For this, we generated a reporter vector containing two TBXT response elements upstream of a minimal promoter that drives the expression of *Firefly* luciferase upon binding of TBXT. Using dual-luciferase reporter assays in U-CH2 and UM-Chor1 chordoma cells expressing endogenous TBXT, 6 of 23 candidate DARPins (A2, B1, B6, C3, D3, and D4) blocked TBXT binding to the response elements, as evidenced by a reduction in the luciferase signal by more than 50% relative to E3_5 (Fig. 1D). These lead DARPins also reduced TBXT binding to the response elements in TBXT-negative human embryonic kidney (HEK) 293 cells expressing exogenous hemagglutinin (HA)-tagged TBXT (Fig. 1D).

Finally, we aimed to provide direct evidence that T-DARPins inhibit the interaction of TBXT with its genomic binding sites in chordoma cells. To this end, we induced expression of E3_5 or the T-DARPins A2, B1, or D4 for 24 hours in UM-Chor1-iDARPin cells (see below) and performed chromatin immunoprecipitation of TBXT followed by quantitative PCR (ChIP-qPCR) of previously identified TBXT binding sites (*8*). Consistent with the expected mode of action of T-DARPins, the binding of TBXT to its targets *KRT8*, *TBXT*, *COL5A2*, *C7orf69*, and *SOX9* was significantly decreased, while a negative control region in *KLK3* was unaffected (Fig. 1E).

Thus, we identified six DARPins, referred to as T-DARPins, that bind TBXT at its DBD, thereby displacing it from DNA and preventing it from binding to TBXT-specific transcription factor binding motifs in cells.

### T-DARPins are specific for TBXT and bind disease-associated TBXT variants

**Fig. 2.**
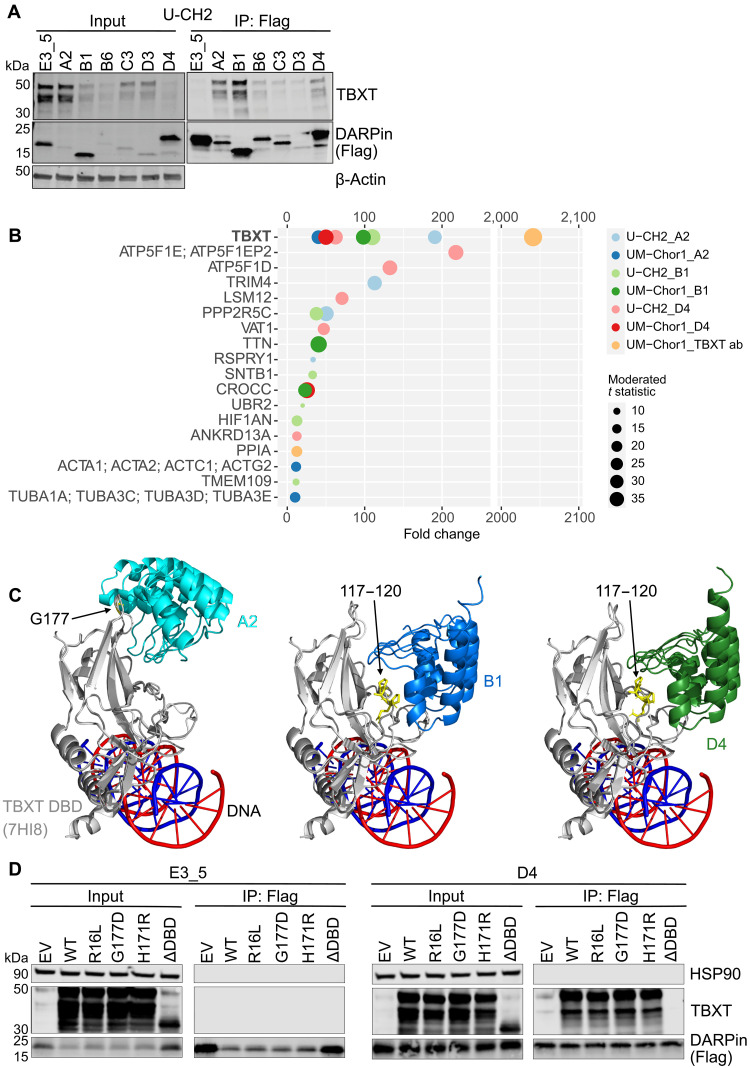
T-DARPins are specific for TBXT and bind TBXT variants. (**A**) IP of Flag-tagged DARPins from U-CH2 cells with an anti-Flag antibody 5 days after transduction, followed by Western blotting (right). The whole-cell lysate input is shown on the left. One representative image of three independent experiments. (**B**) Enrichment of proteins identified by AP-MS of anti-Flag IPs for DARPins A2, B1, D4, and E3_5 and of anti-TBXT antibody (ab) IPs 4 days after transduction of U-CH2 and UM-Chor1 cells. Sphere sizes represent empirical Bayes moderated *t* statistic (*n* = 3) as an indicator of statistical certainty, and bait types and cell lines are indicated by colors. (**C**) Simulation of molecular interactions between T-DARPin structures predicted with AlphaFold2 and the TBXT DBD (PDB: 7HI8). T-DARPin:TBXT DBD (7H18) complexes were overlaid with the TBXT DBD:DNA PDB structure 6F58. The DNA is displayed as blue and red helices. (**D**) Anti-Flag bead pulldowns of DARPin E3_5 and T-DARPin D4 cotransfected into HEK293T cells with EV or a vector expressing HA-tagged WT TBXT, TBXT-R16L, TBXT-G177D, TBXT-H171R, or TBXT lacking the DBD (ΔDBD).

Next, we asked whether the T-DARPins effectively and specifically bind endogenous TBXT in chordoma cells. Immunoprecipitation (IP) of Flag-tagged T-DARPins with an anti-Flag antibody from U-CH2 cell lysate confirmed the binding of TBXT to T-DARPins A2, B1, B6, C3, D3, and D4, whereas E3_5 did not pull down TBXT (Fig. 2A). In the corresponding whole-cell lysates, we noticed that T-DARPin–expressing U-CH2 cells had markedly reduced TBXT levels, which was also observed in U-CH1, JHC7, and UM-Chor1 cells (Fig. 2A and fig. S2, A and B). Together with the finding of reduced *TBXT* mRNA levels (fig. S2C), this suggested that the T-DARPins altered the binding of TBXT to its superenhancer, leading to suppression of TBXT in chordoma cells by disrupting a previously reported autoregulatory loop (*7*, *8*). In addition, the six T-DARPins down-regulated the *YAP1* oncogene, a known transcriptional target of TBXT in chordoma (fig. S2B) (*23*).

We then focused on characterizing T-DARPins A2, B1, and D4, as they showed the strongest binding to TBXT (Fig. 2A), displaced TBXT from DNA in chordoma cells within 24 hours (Fig. 1E), and may act via different modes as seen from the EMSA (D4 versus A2 and B1; Fig. 1C). To determine their selectivity, we immunoprecipitated A2, B1, D4, and E3_5 from U-CH2 and UM-Chor1 cell lysates with an anti-Flag antibody and analyzed the respective T-DARPin interactome by affinity purification mass spectrometry (AP-MS). For comparison, we performed IP with an anti-TBXT antibody. TBXT was the only protein strongly enriched in both cell lines and with all three T-DARPins compared to E3_5 (Fig. 2B). Other proteins were also pulled down by T-DARPins, albeit at much lower levels and typically only by a single T-DARPin in one cell line, rather than consistently across conditions, suggesting they are off-target binders. Of note, we did not detect other members of the T-box transcription factor family, which comprises 17 proteins, including the founding member TBXT (*24*), demonstrating that T-DARPins A2, B1, and D4 are highly selective (Fig. 2B and data S1).

Next, we simulated the binding of T-DARPins to the TBXT DBD to understand the predicted binding modes for T-DARPins A2, B1, and D4. As T-DARPins have not yet been crystallized, structures for A2, B1, and D4 were generated using AlphaFold2 (AF2). To test the precision of this approach, we superimposed the E3_5 AF2 structure over the crystal structure of E3_5 [Protein Data Bank (PDB): 1MJ0] and obtained a root mean square deviation of only 0.363 Å, indicating that AF2 generates accurate DARPin structures (fig. S2D). We then performed protein-protein docking using HADDOCK 2.4 (*25*) with wild-type (WT) TBXT (PDB: 7HI8) and the AF2 structures of A2, B1, and D4. This revealed that the exposed surface binding loops in the variable region of all three T-DARPins were inserted into a groove at the intersection of the three central β sheets of the TBXT DBD (fig. S2E). In addition, the predicted orientation of A2 differs from those of B1 and D4 (Fig. 2C and fig. S2E). The binding loops of A2 reside adjacent to the TBXT loop containing G177 (or G177D), a region hypothesized to facilitate cooperativity between two DBD protomers on the same DNA molecule (*17*) (Fig. 2C). On the other hand, B1 and D4 converge on the same β sheets, but their variable regions interact with the recently identified pocket B spanning residues 117 to 120 of TBXT DBD (*17*). Of note, B1 contains one fewer loop than D4, yet still exhibits similar binding behavior. Together, this suggests two different binding modes of T-DARPins: one in which a T-DARPin (A2) potentially disrupts the interaction interface between two neighboring TBXT DBDs, and another in which T-DARPins (B1 and D4) bind the DBD and may inhibit TBXT function by altering its structure in a way that prevents DNA binding.

Single-nucleotide variants in *TBXT* have been identified in patients with sporadic chordoma and spinal developmental diseases. These include G177D in the TBXT DBD, which is a risk factor for chordoma and present in up to 94% of patients with chordoma, and R16L and H171R, which are associated with congenital scoliosis and sacral agenesis (*10*, *26*–*28*). To investigate whether the T-DARPins bind these clinically relevant TBXT variants, we expressed HA-tagged WT TBXT and TBXT containing the single-nucleotide variants or lacking the DBD together with T-DARPins A2, B1, D4, or E3_5 in HEK293T cells, followed by DARPin IPs and Western blotting. The three T-DARPins bound to all TBXT variants with similar efficiency than WT TBXT, whereas removing the DBD abolished DARPin binding, as expected (Fig. 2D and fig. S2F). Together, we demonstrated the selectivity of T-DARPins for TBXT, including disease-associated TBXT variants, and their predicted binding modes to TBXT DBD.

### T-DARPins impair chordoma cell proliferation, spheroid formation, and tumor growth

**Fig. 3.**
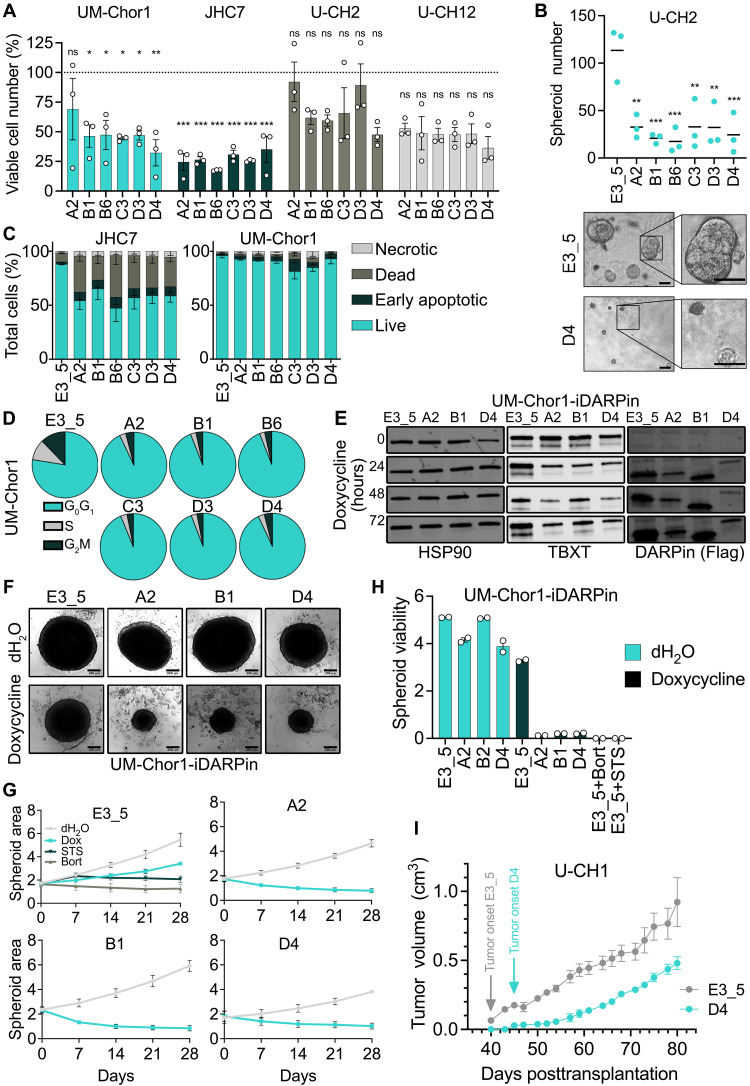
T-DARPins impair the proliferation, spheroid formation, and tumor growth of chordoma cells. (**A**) Viable cell numbers on day 24 after lentiviral transduction of chordoma cell lines with six T-DARPins relative to E3_5. One-way ANOVA with Dunnett’s test; mean ± SEM of three biological replicates. *P ≤ 0.05, **P ≤ 0.01, ***P ≤ 0.001. (**B**) U-CH2 spheroid counts 34 days after transduction with indicated DARPins. Representative images are shown below; scale bar, 50 μm. One-way ANOVA with Dunnett’s test; mean of three biological replicates. *P ≤ 0.05, **P ≤ 0.01, ***P ≤ 0.001. (**C**) Apoptosis in JHC7 and UM-Chor1 cells 10 days posttransduction with DARPins, measured by annexin V/propidium iodide (PI) staining and flow cytometry. Mean ± SEM of two biological replicates. Necrotic, PI^+^/annexinV^−^; early apoptotic, PI^−^/annexin V^+^; dead, PI^+^/annexin V^+^; live, PI^−^/annexin V^−^. (**D**) Cell cycle analysis of UM-Chor1 cells 12 days posttransduction with DARPins. Representative of two biological replicates. (**E**) Western blot of UM-Chor1-iDARPin cells expressing E3_5, A2, B1, or D4 without or with doxycycline (0.5 μg/ml) treatment for 24, 48, and 72 hours. (**F**) Images of UM-Chor1-iDARPin spheroids after 28 days without or with doxycycline (0.5 μg/ml). Representative of three biological replicates. Scale bar, 200 μm. (**G**) Area (μm^2^ × 10^5^) of UM-Chor1-iDARPin spheroids treated with H_2_O, doxycycline (0.5 μg/ml), 10 μM staurosporine (STS), or 1 μM bortezomib (Bort). Mean ± SEM of three biological replicates (four to six spheroids averaged per condition). (**H**) Viability (luminescence units × 10^7^) of UM-Chor1-iDARPin spheroids after 28 days of treatment as in (G), measured with CellTiter-Glo 3D. Mean ± SEM of two biological replicates (four to six spheroids averaged per condition). (**I**) Tumor growth of U-CH1 cells transduced with D4 or E3_5 and injected subcutaneously into NSG mice. Mean volume ± SEM of six tumors per condition until sacrifice of the first mouse due to maximum tumor size.

Next, we investigated the cellular effects of T-DARPins using chordoma cell lines. T-DARPin expression for 24 days reduced viable cells to varying degrees compared to E3_5 under regular culture conditions, with JHC7 and UM-Chor1 being the most affected and U-CH2 and U-CH12 responding the least (Fig. 3A). However, when we grew U-CH2 and U-CH12 cells in three-dimensional (3D) Matrigel cultures, spheroid formation was significantly reduced upon T-DARPin expression (Fig. 3B and fig. S3A) to a similar extent as when TBXT was suppressed by RNA interference (fig. S3B). The T-DARPins induced apoptosis in JHC7 but not U-CH2, UM-Chor1, and U-CH12 cells (Fig. 3C and fig. S3C). In contrast to the other cell lines, JHC7 has a genomic *TBXT* amplification (*5*), which may explain its higher TBXT dependence. In line with the observation that the main cellular consequence of TBXT inhibition is impaired cell proliferation, the T-DARPins increased the proportion of UM-Chor1 cells in the G_0_-G_1_ phase of the cell cycle (Fig. 3D). In addition, we observed morphologic changes, including loss of an organized cytoskeleton, enlarged cells with a pancake-like appearance, and narrow spindle-like cells, indicating senescence or differentiation (fig. S3D). Of note, the T-DARPins localized to the nucleus, ensuring effective TBXT inhibition in cells (fig. S3D). To verify that T-DARPins do not exert general toxic effects, we expressed them in three TBXT-negative nonchordoma cancer cell lines and did not observe reduced cell viability (fig. S3E).

The greater TBXT dependence of chordoma cells in 3D cultures is consistent with TBXT’s function in tissue organization during development (*29*). Because faithful chordoma models for biology research and drug discovery are needed (*12*), we established UM-Chor1 spheroids using ultralow attachment plates and confirmed that they are suited for long-term culture by detecting stable TBXT expression and proliferation, as determined by Ki-67 positivity, after 45 days (fig. S3F). To enable fast induction of T-DARPins and subsequent TBXT inhibition in spheroids, we engineered UM-Chor1 cells, named UM-Chor1-iDARPin cells, to express the tetracycline-responsive transactivator rtTA3 and contain a CMV promoter with multiple doxycycline-responsive elements that drive DARPin expression. We observed tight control of DARPin induction with no expression in the absence of doxycycline and rapid expression of E3_5 and T-DARPins A2, B1, and D4 in the presence of doxycycline (Fig. 3E). The down-regulation of TBXT protein observed after 12 days with lentiviral DARPin delivery (fig. S2, A and B) was seen as early as 24 hours in UM-Chor1-iDARPin cells, suggesting rapid interruption of the TBXT autoregulatory loop (Fig. 3E). Induction of T-DARPins A2, B1, and D4 in 7-day-old UM-Chor1-iDARPin spheroids resulted in complete growth arrest over 28 days, similar to treatment with the broad-spectrum kinase inhibitor staurosporine or the proteasome inhibitor bortezomib, as evidenced by smaller spheroid size and reduced cell viability (Fig. 3, F to H).

To determine the efficacy of T-DARPins in vivo, we injected U-CH1 cells stably expressing E3_5 or T-DARPin D4 subcutaneously into NOD.Cg-*Prkdc^scid^ Il2rg^tm1Wjl^*/SzJ (NSG) mice (fig. S3G). Cells had robust DARPin expression and reduced TBXT levels with T-DARPin D4 before injection (fig. S3H). U-CH1 E3_5 yielded tumors at all six injection sites after 40 days, while the appearance of the first U-CH1 D4 tumor was delayed by 1 week, and tumor growth was slowed in the first 3 weeks compared to E3_5 tumors (Fig. 3I), demonstrating the capacity of T-DARPins to inhibit chordoma growth in vivo. However, the tumors then grew at rates similar to those of the control tumors, suggesting the outgrowth of cells that had developed resistance to TBXT inhibition (Fig. 3I). Consistent with this, D4 tumors had lost DARPin expression and restored TBXT expression at the end of the experiment (fig. S3I). PCR amplification of DARPin transcripts from the tumors revealed that D4 contained a deletion of approximately 100 base pairs, whereas the length of E3_5 transcripts remained unaffected (fig. S3J). Together, these results demonstrated that T-DARPins effectively inhibit endogenous TBXT, resulting in reduced proliferation and spheroid growth of chordoma cells and impaired tumor formation in xenografts.

### T-DARPins cause widespread transcriptomic and proteomic changes

**Fig. 4.**
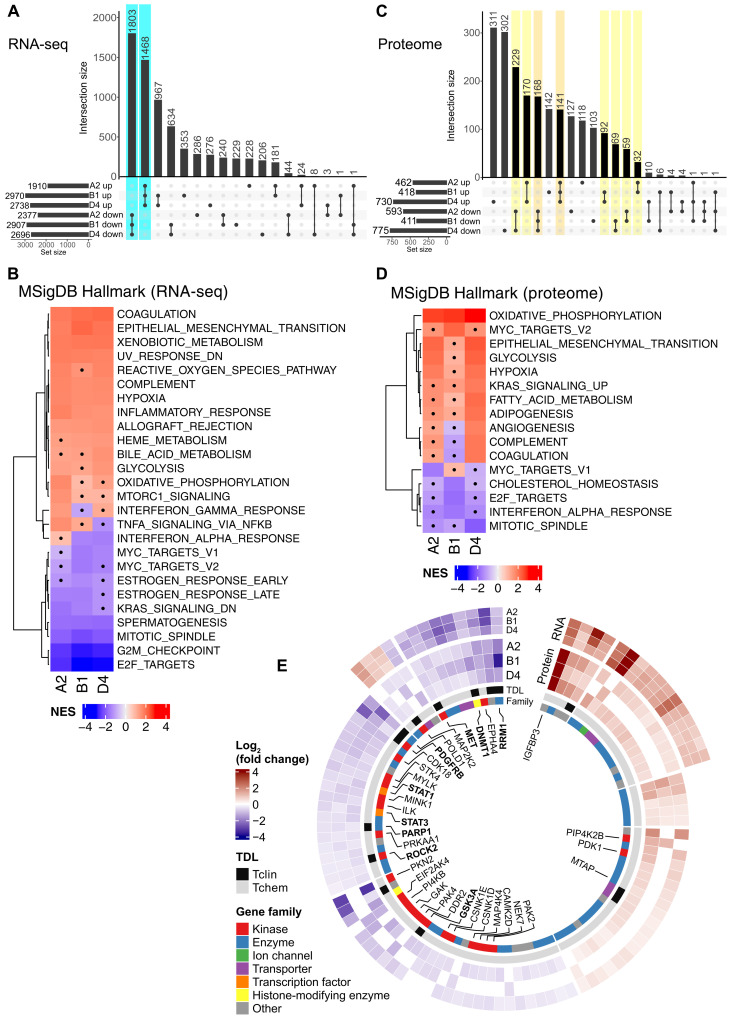
Assessment of the TBXT regulome by T-DARPin expression in UM-Chor1 cells. (**A**) UpSet plot of RNA-seq data showing the number and intersections of genes significantly deregulated by T-DARPin expression as a matrix of six sets (T-DARPins A2, B1, and D4; up- or down-regulated). Rows show the number of genes per set. Columns show the number of intersecting genes at the top and the intersecting sets as connected dots at the bottom. DEGs, as defined in the text, are indicated in teal. Significantly deregulated genes were identified using generalized linear models (GLMs) with a Wald test (*n* = 3, FDR < 1%). (**B**) GSEA of RNA-seq data with the MSigDB Hallmark gene collection. Dots represent enrichments that are not statistically significant based on the permutation test. NES, normalized enrichment score. (**C**) UpSet plot of DDA-MS data showing the number and intersections of proteins significantly deregulated by T-DARPin expression as a matrix of six sets (T-DARPins A2, B1, and D4; up- or down-regulated). Rows show the number of proteins per set. Columns show the number of intersecting proteins at the top and the intersecting sets as connected dots at the bottom. DEPs, as defined in the text, are indicated in orange and yellow. Significantly deregulated proteins were identified using empirical Bayes moderated *t* test (*n* = 3, FDR < 5%). (**D**) GSEA of DDA-MS data with the MSigDB Hallmark gene collection. Dots represent enrichments that are not statistically significant based on the permutation test. (**E**) Circos plot of DEPs categorized according to their target development levels (TDLs). The inner heatmap shows the log_2_(fold change) of proteins deregulated by T-DARPins A2, B1, and D4. The outer heatmap shows the log_2_(fold change) of the corresponding RNA-seq data if the respective DEP-encoding gene was also among the DEGs.

Despite the knowledge that TBXT is the essential chordoma driver, the TBXT-regulated transcriptome is not fully understood. We performed RNA sequencing (RNA-seq) of UM-Chor1 cells transduced with E3_5 or T-DARPin A2, B1, or D4. The changes induced by the three T-DARPins relative to E3_5 were highly concordant (fig. S4A and data S2). We found 1803 significantly down-regulated and 1468 significantly up-regulated genes [log_2_(fold change) > 0.585 or <−0.585 (corresponding to a 1.5-fold change), false discovery rate (FDR) < 1%] that were common to the three T-DARPins (Fig. 4A and data S2), demonstrating that TBXT has widespread transcriptional properties in chordoma cells.

Among the 3271 differentially expressed genes (DEGs) were TBXT targets previously identified by non–protein-based methods such as CRISPR knockout or degron tagging (*7*, *8*), further validating the on-target activity of T-DARPins. These included the embryonic notochord markers *ACAN*, *COL2A1*, *SOX9*, and *TBXT* themselves, which are abnormally active in chordoma cells (*30*); *TPX2*, *C7orf69*, *WEE1*, *ATP6V1B2*, and *SAE1*, which are controlled by binding of TBXT to their superenhancers (*7*, *8*); collagen genes associated with the embryonic notochord (*COL17A1* and *COL26A1*) (*31*) or chordoma (*COL11A2*) (*32*); and the chordoma-associated cytokeratin genes *KRT18* and *KRT19* (*8*, *30*) (fig. S4, B and C). Gene set enrichment analysis (GSEA) using the Hallmark, ARCHS^4^ Tissue, and Gene Ontology Biological Process (GO_BP) gene signature collections (Fig. 4B; fig. S4, D to F; and data S3) revealed significant suppression of networks related to cell cycle checkpoint regulation, cell division, and DNA replication and repair (Fig. 4B and fig. S4E) and of gene signatures associated with embryonic stem and progenitor cells, most likely reflecting TBXT-regulated gene programs of remnant notochordal cells (fig. S4D). Gene signatures up-regulated upon TBXT inhibition were mainly associated with differentiated cell types, e.g., of muscle or neuronal origin, and epithelial-mesenchymal transition (Fig. 4B and fig. S4D), hinting toward differentiation of chordoma cells after TBXT inhibition, and with various metabolic processes and inflammatory response (Fig. 4B and fig. S4F).

We next used data-dependent acquisition mass spectrometry (DDA-MS) to determine the TBXT-dependent proteome in UM-Chor1 cells following expression of T-DARPins A2, B1, or D4 for 14 days or CRISPR-Cas9–mediated TBXT knockout (fig. S4, G and H). We identified 960 significantly differentially expressed proteins [DEPs; log_2_(fold change) > 0.2 or <−0.2 (corresponding to a 1.15-fold change), FDR < 5%] associated with at least two T-DARPins and regulated in the same direction, of which 525 were down-regulated and 435 were up-regulated (Fig. 4C, fig. S4I, and data S4). Of these, 523 were also significantly deregulated in the same direction by TBXT knockout (256 down-regulated and 267 up-regulated; data S4). Most prominent was the extreme up-regulation of insulin-like growth factor binding protein 3 (IGFBP3), which was present in all four conditions (fig. S4I). GSEA of T-DARPin proteomes using the same gene signature collections as for the RNA-seq analysis (Fig. 4D; fig. S4, J to L; and data S5) revealed suppression of networks involved in cell cycle regulation, DNA replication and repair, and interferon alpha response after TBXT inhibition (Fig. 4D and fig. S4K) and up-regulation of signatures associated with differentiated cell types, epithelial-mesenchymal transition, and metabolic processes, particularly oxidative phosphorylation and glycolysis (Fig. 4D and fig. S4, J and L), which was largely consistent with the result of GSEA of T-DARPin transcriptomes. Together, TBXT inhibition caused substantial changes in the transcriptome and proteome of chordoma cells indicative of impaired cell division, a switch from an embryonic to a more differentiated cell state, an interferon/inflammatory response, the induction of various metabolic processes, and perturbed DNA replication and repair.

### The TBXT regulome includes potential pharmacologic targets

In addition to TBXT itself, essential downstream effectors may also represent therapeutic targets that could be addressed alone or in combination with future TBXT-directed drugs. To identify such candidate drug targets, we used The Cancer Druggable Gene Atlas (*33*) and categorized the TBXT-regulated DEGs and DEPs according to the target development levels Tclin (clinically approved drugs available), Tchem (experimental drugs available), Tbio (potentially druggable), and Tdark (currently not druggable). Overall, 1065 DEGs and/or DEPs could be assigned to any of the four categories, of which 102 met the Tclin level and 291 met the Tchem level (data S6). Tclin and Tchem targets were identified in both the transcriptome and proteome (*n* = 6 and *n* = 23, respectively), the proteome only (*n* = 8 and *n* = 63, respectively), and the transcriptome only (*n* = 88 and *n* = 205, respectively; fig. S4M and data S6). As proteins are the direct targets of drugs, we subsequently focused on DEPs with Tclin (*n* = 14) and Tchem (*n* = 86) levels and not on DEGs (Fig. 4E). However, the large group of candidates identified only by RNA-seq may include relevant targets, such as the clinically actionable kinases RET, NTRK1, and CDK6 (fig. S4M).

Of the 100 Tclin/Tchem DEPs, 60 were suppressed by TBXT inhibition and, therefore, represent effectors directly or indirectly elevated by TBXT. Among these were 24 kinases, e.g., MET, GSK3A, ROCK2, and PDGFRB; the enzymes RRM1 and PARP1; and the DNA methyltransferase DNMT1 (Fig. 4E and data S6). While PDGFRB and PARP1 inhibitors have been applied as chordoma therapies (*13*, *34*), the biological and clinical role of the other candidates is unclear. The receptor tyrosine kinase MET and its ligand hepatocyte growth factor are strongly expressed in primary chordoma samples, and chordoma cell lines, particularly of sacral origin, are sensitive to MET inhibition (*35*). RRM1, the large catalytic subunit of ribonucleotide reductase responsible for generating deoxynucleoside triphosphates for DNA synthesis, is inhibited by gemcitabine (*36*), which is used to treat various cancers but whose efficacy in chordoma has not yet been tested. Similarly, the catalytic activity of DNMT1 can be blocked by azacitidine or decitabine, both used to treat acute myeloid leukemia. Finally, DEPs positively regulated by TBXT also included the transcription factors STAT1 and STAT3, which can be modulated by inhibiting upstream regulatory JAK kinases, for which several approved drugs are available.

A total of 40 Tclin/Tchem DEPs were up-regulated by TBXT inhibition and are thus directly or indirectly suppressed by TBXT in chordoma cells. Among these was the 5-methylthioadenosine phosphorylase MTAP (Fig. 4E). The *MTAP* gene is codeleted with the tumor suppressor gene *CDKN2A* in multiple cancer types, causing sensitivity to inhibition of the protein methyltransferase PRMT5 (*37*, *38*), and several PRMT5 inhibitors are being tested in clinical trials. It is, therefore, tempting to speculate that TBXT-mediated MTAP suppression may phenocopy this effect in chordoma. In addition, several cathepsins (CTSA, CTSB, and CTSL) were among the Tchem proteins induced by TBXT inhibition. Cathepsins are frequently implicated in cancer development and represent emerging drug targets (*39*). Finally, the most up-regulated candidate in both the proteome and the transcriptome was IGFBP3, which belongs to the Tchem category. In summary, the TBXT regulome contains multiple pharmacologically tractable proteins, including some targets of clinically approved drugs, and therefore represents a valuable resource for future chordoma drug discovery campaigns.

### The TBXT regulome is partially modulated by IGFBP3

**Fig. 5.**
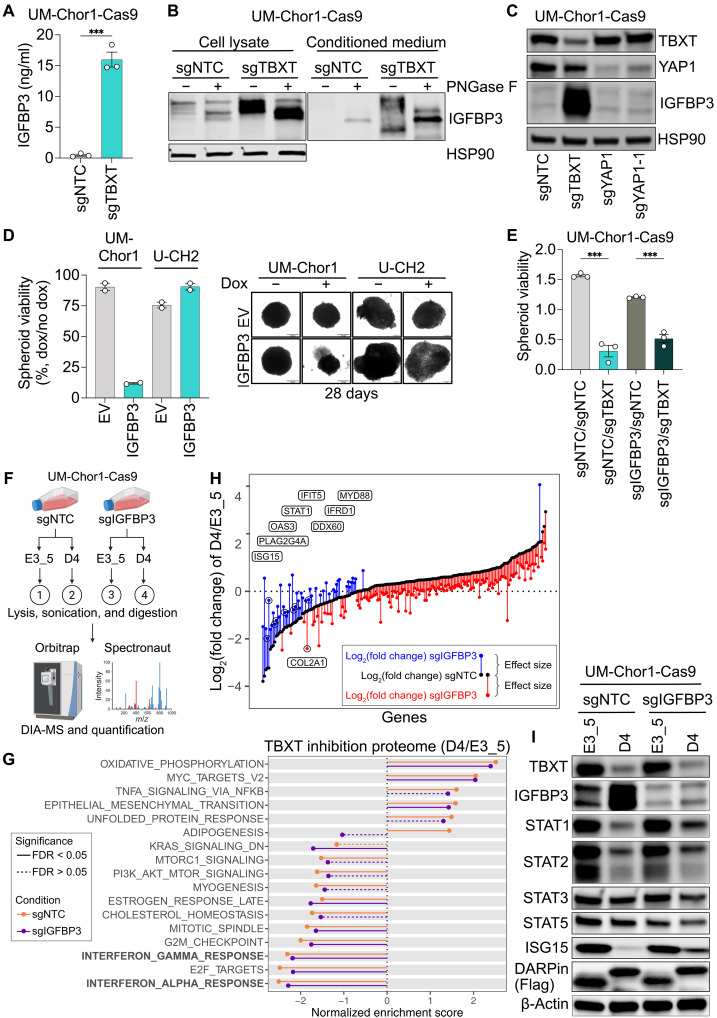
IGFBP3 modifies part of the TBXT regulome. (**A**) IGFBP3 protein levels quantified by ELISA of UM-Chor1-Cas9 cells transduced with sgNTC or sgTBXT. Mean ± SEM of three biological replicates. Unpaired *t* test. ***P ≤ 0.001. (**B**) IGFBP3 protein levels in lysates and culture medium of UM-Chor1-Cas9 cells transduced with sgNTC or sgTBXT, with or without PNGase F treatment. (**C**) Western blot of UM-Chor1-Cas9 cells transduced with sgNTC, sgTBXT, or two sgRNAs targeting YAP1. (**D**) Viability of UM-Chor1 and U-CH2 spheroids expressing doxycycline-inducible IGFBP3 or EV, treated with doxycycline (0.5 μg/ml) versus untreated controls after 28 days. Mean ± SEM of two biological replicates (five to six spheroids averaged per condition). Representative spheroid images shown on the right. (**E**) Viability (luminescence units × 10^7^) after 14 days of UM-Chor1-Cas9 spheroids double-transduced with sgNTC/sgNTC, sgTBXT/sgNTC, sgIGFBP3/sgNTC, or sgIGFBP3/sgTBXT. Mean ± SEM of three biological replicates (four to six spheroids averaged per condition). One-way ANOVA with Dunnett’s test for multiple comparisons; ***P ≤ 0.001. (**F**) Schematic of DIA-MS analysis in UM-Chor1-Cas9 cells 14 days posttransduction with sgNTC or sgIGFBP3, followed by T-DARPin D4 or E3_5, resulting in four conditions: ① control, ② TBXT inhibition, ③ IGFBP3 knockout, and ④ combined TBXT inhibition and IGFBP3 knockout. Created in BioRender. S.F. (2025) BioRender.com/r29p687. (**G**) MSigDB Hallmark GSEA comparing sgNTC (orange) versus sgIGFBP3 (purple) UM-Chor1-Cas9 proteomes under TBXT inhibition. The *x* axis shows NESs for gene sets significantly enriched in at least one condition (FDR < 5%, permutation test with Benjamini-Hochberg correction). NES values for both conditions are shown; dashed lines indicate FDR > 5%. (**H**) Expression changes of IGFBP3-dependent genes induced by T-DARPin D4 in sgNTC (black) or sgIGFBP3 (red and blue) UM-Chor1-Cas9 cells. Vertical lines indicate differences in log_2_(fold change). Red indicates reduced, blue increased expression upon IGFBP3 knockout (GLM with Wald test, *n* = 3, FDR < 1%). Interferon response genes are indicated and data points are circled. (**I**) Western blot of UM-Chor1-Cas9 cells with (sgIGFBP3) or without (sgNTC) IGFBP3 knockout and expressing DARPins E3_5 or D4.

IGFBP3 is a signaling molecule implicated in cell survival and apoptosis, depending on cellular context (*40*), and is part of the senescence-associated secretory phenotype (SASP) (*41*). It is secreted and glycosylated and exerts para- and autocrine effects (*40*). To study the role of IGFBP3 in chordoma, we first confirmed its up-regulation using ELISA in UM-Chor1 cells after TBXT knockout (Fig. 5A) and in JHC7 and U-CH2 cells after TBXT inhibition with T-DARPins (fig. S5A). IGFBP3 was also detected in conditioned medium of UM-Chor1 and U-CH2 cells, particularly after TBXT loss, and treatment with the glycosidase PNGase F decreased the molecular weight of IGFBP3 (Fig. 5B and fig. S5B). This indicated that IGFBP3 is secreted and glycosylated in chordoma cells, particularly evident after TBXT loss. Because IGFBP3 is a YAP1 target (*42*), and YAP1 is transcriptionally regulated by TBXT (*23*), we examined whether IGFBP3 induction upon TBXT inhibition is mediated via YAP1. sgRNA knockout or shRNA knockdown of YAP1 in UM-Chor1 cells did not affect IGFBP3 mRNA and protein expression, whereas TBXT knockout increased *IGFBP3* mRNA as expected (Fig. 5C and fig. S5, C and D), demonstrating that the reciprocal relationship between TBXT and IGFBP3 expression is independent of YAP1. To explore a potential direct regulation of *IGFBP3* by TBXT, we examined histone 3 lysine 27 acetylation (H3K27ac) and HA-dTAG-TBXT ChIPmentation data from UM-Chor1 cells generated by Sheppard *et al.* (*8*). We identified multiple H3K27ac-enriched regions upstream of the *IGFBP3* locus, suggesting the presence of two candidate enhancers occupied by TBXT (fig. S5E). Using TBXT ChIP-qPCR with primers flanking the two candidate enhancers in UM-Chor1-iDARPin cells after a 24-hour induction of E3_5 or T-DARPins A2, B1, and D4, we confirmed the binding of TBXT to these two elements, which was disrupted by T-DARPins (fig. S5F). These data suggest that TBXT directly regulates *IGFBP3* in chordoma.

We next hypothesized that high IGFBP3 levels might be toxic to chordoma cells and, therefore, suppressed by TBXT and that the proliferation arrest caused by TBXT inhibition might be mediated by IGFBP3 reexpression. Consistent with this, doxycycline-induced IGFBP3 overexpression decreased the viability and size of UM-Chor1 spheroids after 28 days. In contrast, U-CH2 spheroids were unaffected (Fig. 5D), suggesting a cell line– and chordoma type–specific effect of IGFBP3. Additional depletion of IGFBP3 partially restored TBXT knockout-induced UM-Chor1 spheroid viability (Fig. 5E), indicating that IGFBP3 up-regulation contributes to the growth-inhibitory effect of TBXT inhibition.

To investigate the influence of IGFBP3 on the TBXT regulome at high resolution, we performed data-independent acquisition mass spectrometry (DIA-MS) in UM-Chor1 cells stably transduced with an sgRNA targeting IGFBP3 (sgIGFBP3) or a nontargeting control sgRNA (sgNTC) in combination with T-DARPin D4 or the control DARPin E3_5 (Fig. 5F and data S7). In sgNTC cells (no IGFBP3 knockout), 3501 proteins were deregulated (1752 up and 1749 down) by TBXT inhibition via D4 relative to E3_5 (condition ② versus ①, Fig. 5F) [log_2_(fold change) > 0.2 or <−0.2 (corresponding to a 1.15-fold change), FDR < 5%]. With IGFBP3 knockout (condition ④ versus ③, Fig. 5F), fewer proteins (*n* = 2959) were deregulated by TBXT inhibition (1373 up and 1586 down), of which 81% overlapped with the proteins deregulated in sgNTC cells (fig. S5G). GSEA with proteins regulated by TBXT in unperturbed and IGFBP3 knockout cells using the Hallmark gene signature collection revealed enrichment of oxidative phosphorylation and epithelial-mesenchymal transition and suppression of cell cycle–related networks and interferon response in both conditions, among other gene sets (Fig. 5G), in line with the effects of TBXT inhibition found by DDA-MS (Fig. 4D). However, IGFBP3 knockout generally mitigated the changes induced by TBXT inhibition. For example, in IGFBP3-unperturbed (sgNTC) cells, TBXT inhibition markedly down-regulated interferon alpha and gamma responses (Fig. 5G; orange lines). Upon IGFBP3 knockout, the TBXT inhibition-induced down-regulation persisted, but to a lesser extent (Fig. 5G; purple lines). These analyses suggest that IGFBP3 affects certain subsets of proteins regulated by TBXT.

To determine the proteins whose levels are influenced by TBXT activity and IGFBP3 expression, we analyzed the DIA-MS data by multifactorial linear regression with interactions. In short, we isolated the effect of TBXT inhibition from the effect of IGFBP3 loss. This identified 193 proteins, of which 144 (75%) were suppressed and 49 (25%) were up-regulated after IGFBP3 loss (Fig. 5H). Notably, most of the proteins down-regulated by IGFBP3 loss were up-regulated by TBXT inhibition and vice versa, suggesting that IGFBP3 mainly counteracts the regulatory effects of TBXT (Fig. 5H). Among the modulated proteins was the embryonic notochord marker COL2A1, whose TBXT inhibition-induced suppression was further enhanced by IGFBP3 loss, whereas other notochord markers, including TBXT itself, were unaffected by IGFBP3 (Fig. 5H and fig. S5H). Eight modulated proteins are involved in the interferon response pathway, including STAT1 and the interferon-stimulated genes (ISGs) ISG15, OAS3, IFRD1, and IFIT5, whose expression was down-regulated by TBXT inhibition and partially restored by IGFBP3 loss (Fig. 5H). Western blot analysis showed that TBXT inhibition strongly down-regulated STAT1, STAT2, and ISG15 in UM-Chor1 cells, which was partially reversed in the absence of IGFBP3 in the case of STAT1 and ISG15 but not STAT2, confirming the proteomics results (Fig. 5I). For completeness, we also blotted for STAT3 and STAT5 and found a slight decrease in both proteins upon TBXT inhibition, which was enhanced in the absence of IGFBP3.

Together, we showed that part of the TBXT regulome is modulated by IGFBP3, particularly factors involved in the interferon response, suggesting that IGFBP3 can fine-tune the action of TBXT to precisely fulfill the requirements of chordoma cells for survival and proliferation.

### TBXT promotes JAK-STAT signaling and renders chordoma cells sensitive to JAK2 inhibition

**Fig. 6.**
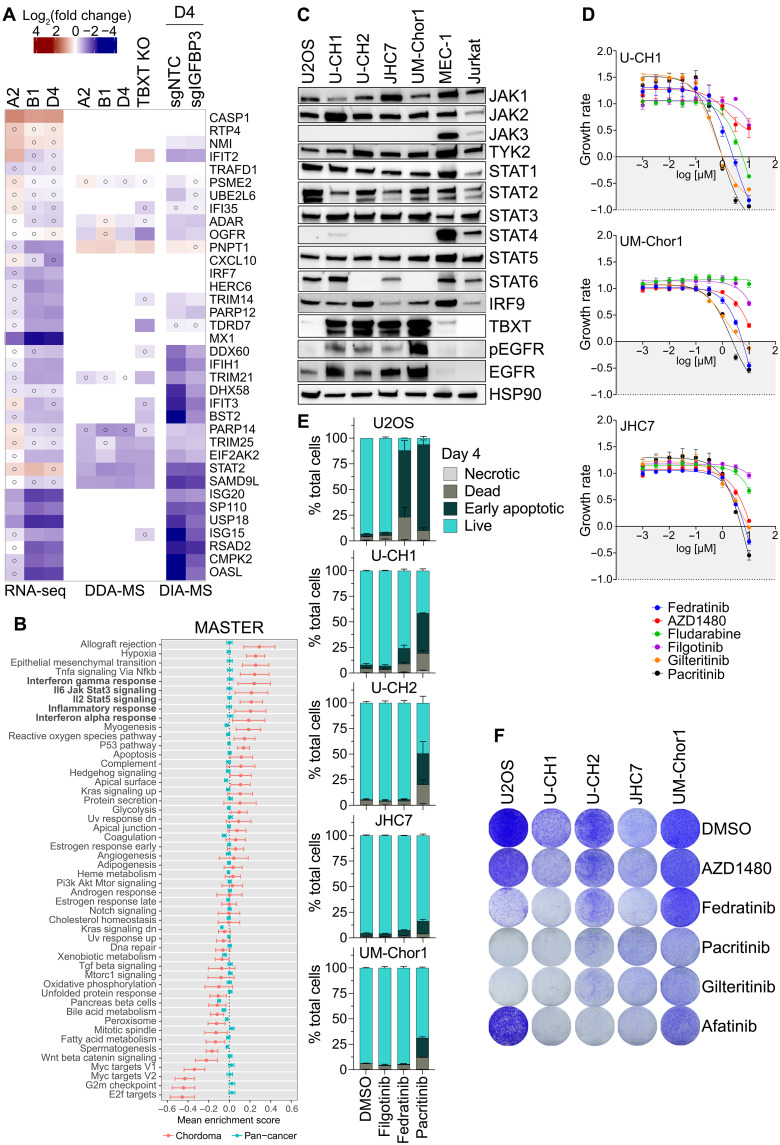
Chordoma cells depend on JAK-STAT signaling. (**A**) ISG expression in UM-Chor1 cells as determined by RNA-seq, DDA-MS, and DIA-MS under different treatments shown at the top. Open circles indicate nonsignificant expression [RNA-seq: GLM with Wald test (*n* = 3, FDR < 1%); DDA-/DIA-MS: empirical Bayes moderated *t* test (*n* = 3, FDR < 5%)]. (**B**) GSEA of 1107 tumor transcriptomes (1106 patients) using 50 Hallmark gene sets. Mean gene set enrichment scores of 23 chordoma samples (red) versus 1084 nonchordoma samples (blue). Gene sets ranked by mean enrichment score in chordoma. Error bars: 95% confidence intervals (*t* distribution). (**C**) Western blots of chordoma cell lines U-CH1, U-CH2, JHC7, and UM-Chor1; the osteosarcoma cell line U2OS; the B cell leukemia cell line MEC-1; and the T cell leukemia cell line Jurkat showing JAK/STAT pathway members, TBXT, EGFR, and phospho-EGFR (Y1068). (**D**) GR analysis after 7 days of treatment with pan-JAK (filgotinib), JAK2 (AZD1480, fedratinib, and pacritinib), STAT1 (fludarabine), and FLT3 (gilteritinib) inhibitors (10 μM to 1 nM). GR < 1 and > 0: proliferation inhibition; GR = 0: cytostasis; GR < 0: cytotoxicity. One of three representative experiments shown; data points represent technical triplicates. (**E**) Apoptosis measured by annexin V/7-AAD staining and flow cytometry after 4-day treatment with 3 μM of indicated drugs. Mean + SEM of two biological replicates. (**F**) Colony formation of U2OS and chordoma cell lines after 14-day treatment with 1 μM of indicated drugs. Representative images of three independent experiments (fig. S6D).

The multiomics experiments described above pointed to a functional link between TBXT and interferon signaling, particularly the JAK-STAT axis. Specifically, we found interferon response signaling to be significantly affected by TBXT inhibition on the transcriptomic (Fig. 4B) and proteomic (Figs. 4D and 5G) level, and observed down-regulation of STATs and ISGs after TBXT inhibition (Fig. 5, H and I). To substantiate this association, we mapped the levels of ISGs across the RNA-seq and proteomics experiments and observed down-regulation of ISGs upon TBXT inhibition in all datasets (Fig. 6A), suggesting that chordoma cells might have high baseline interferon signaling driven by TBXT. To ensure that this was not only a feature of chordoma cell lines, we analyzed RNA-seq data of 1107 tumor samples from 1106 patients with cancer, including 23 samples from 22 patients with chordoma, enrolled in DKFZ/NCT/DKTK MASTER, a cross-entity precision oncology program focusing on rare cancers (*43*, *44*). In line with the observations in cell lines, MSigDB enrichment scores revealed that interferon alpha and gamma response, inflammatory response, and IL6/JAK/STAT3 and IL2/STAT5 signaling were among the most enriched signaling axes in chordoma but not in the pan-cancer cohort (Fig. 6B).

To better understand JAK-STAT activity in chordoma, we examined the levels of JAK and STAT family proteins in multiple chordoma cell lines, the osteosarcoma cell line U2OS, the chronic B cell leukemia cell line MEC-1, and the T cell leukemia cell line Jurkat. U2OS was selected as a nonchordoma sarcoma cell line sensitive to JAK inhibition (*45*), and MEC-1 and Jurkat were chosen as lymphoid cells with intrinsically high JAK/STAT signaling. As expected, all chordoma cell lines lacked JAK3, which is only expressed in hematopoietic lineages (*46*). They also lacked STAT4 but expressed JAK1, JAK2, and TYK2 to varying degrees, as well as STAT1, STAT2, STAT3, and STAT5 to similar levels as MEC-1 and Jurkat (Fig. 6C). STAT6 was expressed in U-CH1 and JHC7 cells but not UM-Chor1 or U-CH2 cells (Fig. 6C). In addition, all chordoma cell lines expressed IRF9, which forms a complex with activated STAT1 and STAT2 that stimulates the transcription of ISGs (*46*).

On the basis of these results, we reasoned that chordoma cells might be dependent on members of the JAK-STAT pathway, representing a novel vulnerability that can be addressed by clinically approved drugs. To test this, we measured the sensitivity of chordoma cells to the pan-JAK inhibitor filgotinib (*46*); the JAK2 inhibitors AZD1480 (*46*), fedratinib (*46*), and pacritinib (*46*); the chemotherapeutic and STAT1 inhibitor fludarabine (*47*); and the FLT3 inhibitor gilteritinib as a control (*48*), because fedratinib and pacritinib also inhibit FLT3. JAK-dependent U2OS cells were used as a positive control. Drug responses were calculated using growth rate (GR) analysis (*49*) to account for the highly variable cell division rates of chordoma cell lines. While fludarabine, AZD1480, and filgotinib were mainly cytostatic, the JAK2 inhibitors fedratinib and pacritinib had the strongest effect on all cell lines and pushed the GR below 0, indicating a cytotoxic effect (Fig. 6D and fig. S6A). Unexpectedly, gilteritinib was equally toxic, although chordoma cells do not express FLT3 (fig. S6B). This is likely due to a nonspecific effect of gilteritinib and may be caused by off-target inhibition of other proteins, the investigation of which could potentially lead to insights into alternative chordoma vulnerabilities.

Consistent with their predicted cytotoxicity, fedratinib and pacritinib, but not the pan-JAK inhibitor filgotinib, induced apoptosis in U-CH1 cells after 4 days, which increased after 7 days (Fig. 6E and fig. S6C). In addition, pacritinib induced apoptosis in U-CH2, UM-Chor1, and JHC7 cells albeit to a lesser degree. Furthermore, we observed markedly reduced colony formation over 14 days after JAK2 inhibition in U2OS and chordoma cells, particularly U-CH1, with pacritinib being more effective than fedratinib (Fig. 6F and fig. S6D). The EGFR inhibitor afatinib, which we included as a positive control because it down-regulates TBXT (*14*, *16*), was equally harmful to chordoma cells but, as expected, not to the TBXT-negative and EGFR-inactive U2OS cells (Fig. 6C). The FLT3 inhibitor gilteritinib also strongly inhibited colony formation. In summary, our data indicate that chordoma cells require JAK-STAT signaling driven by TBXT, particularly JAK2, suggesting an immediately applicable strategy for targeted treatment of patients with chordoma using clinically approved JAK2 inhibitors.

## DISCUSSION

TBXT plays a crucial role in regulating chordoma cell function. Consequently, it is an attractive target for developing antichordoma drugs, which should provide a substantial therapeutic window due to the largely absent TBXT expression in normal tissues. However, many transcription factors, like TBXT, evade pharmacologic targeting. Thus, new approaches are needed to find targeted therapies for transcription factor-driven cancers. We developed DARPins that effectively and selectively bind TBXT and inhibit its transcriptional function in chordoma cells. Using these novel agents, we determined the TBXT regulome and uncovered components of TBXT-dependent signaling networks that may represent therapeutic vulnerabilities.

We developed T-DARPins for two reasons: to generate tools for studying the function of TBXT in chordoma or other TBXT-expressing cancers in a highly specific manner and to provide the basis for molecular mechanism–instructed therapies. T-DARPins A2, B1, and D4 were highly concordant in blocking the transcriptional activity of TBXT by rapidly impairing its binding to DNA and down-regulating TBXT mRNA and protein. While binding to clinically relevant TBXT variants associated with chordoma and developmental disorders of the axial skeleton was retained, the T-DARPins were highly selective, as they did not bind T-box homologs of TBXT, and only few off-target binders were observed that were mainly unique to each T-DARPin. Furthermore, protein structure and T-DARPin-TBXT docking predictions showed that A2 displays a different binding geometry to TBXT DBD than B1 and D4, suggesting inhibition of TBXT activity through different mechanisms, i.e., disruption of TBXT dimerization and impairment of DNA binding due to conformational changes, respectively. However, the structural modeling and inferred mode of action require future validation using biophysical methods and/or crystallization followed by functional studies.

So far, DARPins have been developed against secreted, cell surface, and cytoplasmic targets (*19*, *21*, *50*, *51*), and some molecules have entered clinical trials (*19*, *51*). Our T-DARPins demonstrate that nuclear proteins can also be bound and blocked by DARPins without adding a nuclear localization signal. On the basis of these properties, T-DARPins would, in principle, be suitable as therapeutic agents. However, the delivery into tumor cells is challenging for further clinical development. Potential strategies include fusion with bacterial toxins that allow translocation from the endosome to the cytosol, as demonstrated in cell culture, fusion with cell-penetrating peptides, fusion with domains forming a complex with cationic and ionizable lipids via electrostatic interactions, mRNA-based approaches, and viral systems, e.g., shielded adenoviruses that minimize targeting in the liver and recognition by the immune system (*20*, *52*–*55*). In addition to a potential role as clinically applied biologics, T-DARPins could also serve as a starting point for developing TBXT-directed small molecules.

As expected, owing to the essential role of TBXT in chordoma biology (*7*, *8*, *14*, *23*, *26*, *30*), we observed impaired cell growth by T-DARPin–mediated TBXT inhibition in various experimental models. This effect was stronger in 3D models, highlighting the importance of using such formats for chordoma drug discovery, as also demonstrated in a recent proof-of-concept study testing drug sensitivity in patient-derived chordoma organoids (*12*). In addition, T-DARPin D4 delayed tumor onset and initial growth in a U-CH1 xenograft. The finding that surviving U-CH1 cells lost T-DARPin expression by acquiring a deletion in the D4 transgene illustrated the selection pressure to restore TBXT.

The predominant mechanism by which T-DARPins impaired chordoma cell growth was cell cycle disruption, as evidenced by G_0_-G_1_ arrest and the most enriched signaling networks in the TBXT-regulated transcriptome and proteome. The effect on the cell cycle and the morphologic changes after TBXT inhibition suggest that chordoma cells undergo senescence—a fate that has been previously reported (*5*, *8*). One chordoma cell line, JHC7, underwent apoptosis upon T-DARPin expression. This exception may be related to genomic *TBXT* amplification in these cells (*5*), which could be associated with particularly strong TBXT dependence. In addition, some UM-Chor1 cells assumed an elongated, neuron-like shape after T-DARPin expression, indicating differentiation. This was also supported by the transition from transcriptional programs resembling those of embryonic stem cells to those of differentiated cell types. Because TBXT is an essential driver of mesoderm formation and notochord development (*9*), its continued expression and transcriptional activity in chordoma leads to the persistence of an immature cellular phenotype (*8*). Consequently, TBXT inhibition could impose a more differentiated identity on chordoma cells. UM-Chor-1 cells seemed to be driven toward the neural lineage. However, additional studies with multiple chordoma cell lines or primary chordoma organoids are needed to investigate all possible fates of chordoma cells after TBXT inhibition and to develop biomarkers of differentiation for guiding future TBXT-targeted therapies.

Taking into account the transcripts and proteins deregulated by three independent T-DARPins allowed us to confidently map the TBXT regulome of UM-Chor-1 chordoma cells. Besides the main impact of TBXT on the cell cycle and identity of chordoma cells, analysis of the core TBXT regulome identified additional gene programs. In particular, glycolysis and oxidative phosphorylation were up-regulated after TBXT blockade in both the transcriptome and proteome. Two previous studies have also suggested a role for metabolic processes in chordoma. Using CRISPR-Cas9 screening in chordoma cell lines, Sharifnia *et al.* (*56*) identified essential genes related to glucose, amino acid, and cholesterol metabolism. Proteomic analysis of biopsies from patients with chordoma revealed that oxidative phosphorylation was strongly deregulated (*57*). Further studies are needed to determine the exact role of these pathways in chordoma biology and their potential for clinical exploitation.

By assigning target development levels to all genes and proteins in the TBXT regulome, we identified candidates that could be exploited as therapeutic entry points, either alone or in combination with future anti-TBXT treatments to increase efficacy. This strategy recovered known targets of small-molecule inhibitors, including PDGFRB, CDK6, and PARP1, that have been (*13*, *34*) or are being (ClinicalTrials.gov: NCT03110744 and NCT03127215) tested in clinical trials enrolling patients with chordoma, demonstrating the potential to identify druggable molecules among the TBXT-regulated proteins. At present, chordoma remains an orphan malignancy with no approved drugs. It is intrinsically resistant to conventional chemotherapy, and all molecularly targeted agents tested to date, including PDGFR/VEGFR-directed multikinase inhibitors, EGFR inhibitors, CDK4/6 inhibitors, and mTOR inhibitors, have primarily achieved transient disease stabilization, with objective responses being rare. In light of this persistent therapeutic challenge, we propose that durable clinical benefit will likely require both the identification of improved therapeutic targets, such as TBXT and its downstream effectors, and the development of rational combination regimens. In this context, promising strategies include targeting JAK2 (discussed below); inhibition of MET, whose antichordoma activity has been shown preclinically (*35*); repurposing DNMT1 inhibitors approved for acute myeloid leukemia; inhibition of PRMT5 in the setting of TBXT-induced MTAP suppression (*37*, *38*); and treatment with cytotoxic drugs that inhibit RRM1 (*36*), such as gemcitabine.

In addition to immediately clinically actionable targets, the TBXT regulome contained many potentially druggable candidates. IGFBP3 stood out for its extreme up-regulation after TBXT loss in several chordoma cell lines. Of note, IGFBP3 is also up-regulated in Ewing sarcoma cells after suppression of the EWS::FLI1 fusion (*58*), indicating that it may be a more common downstream effector of oncogenic transcription factors in mesenchymal malignancies. Given that IGFBP3, among other IGFBP family members, is a component of the SASP (*41*), its up-regulation following oncogene inhibition may reflect a senescence response. However, our data also support a more direct regulatory relationship: TBXT may act as an upstream transcriptional regulator of *IGFBP3* by binding to regulatory elements, potentially explaining its outlier expression in response to T-DARPin expression in chordoma cells. IGFBP3 is an intra- and extracellular signaling protein with pro- and antitumor activity. It displaces IGF1 from its receptor, thereby inhibiting the IGF1-IGF1R signaling cascade and reducing cell growth (*40*). However, IGFBP3 has also been proposed to have IGF-independent functions, potentially explaining its pleiotropic effects in different cellular contexts. For example, it interacts with TGF-β signaling, binds to its own receptor mediating apoptosis, is suspected to be transported into the nucleus via importin-β to act as a transcription factor or transcriptional coregulator, where it could influence many cellular processes, including DNA repair, and can be cleaved by metalloproteases, with the resulting fragments having diverse functions (*40*). We found that IGFBP3 is glycosylated and secreted in chordoma cells, especially when it is highly expressed after TBXT inhibition. Our initial data on IGFBP3 function in chordoma cells showed that it impairs spheroid growth and that its knockout partially reversed the growth-inhibitory effects of TBXT loss and modulated a subset of the TBXT-regulated proteome. This included components of a tightly regulated network responsible for interferon signaling upon viral infection or cytokine stimulation. In summary, IGFBP3 may act as a regulator that fine-tunes specific transcriptional effects of TBXT in chordoma cells to create optimal growth conditions.

Our data are consistent with the recent observation that chordoma cells have high ISG scores (*56*), which reflect the activity of genes directly downstream of the interferon-α/β and γ signaling pathways (*59*). On the basis of comprehensive gene and protein expression analysis, we demonstrated that TBXT inhibition results in markedly lower ISG scores, possibly caused by lowering the transcription factors STAT1 and STAT2, which usually activate ISGs after phosphorylation by JAK family proteins and complex formation. These results have led us to conclude that TBXT directly or indirectly activates the interferon response pathway in chordoma cells and that IGFBP3 fine-tunes the TBXT-directed modulation of STAT proteins. When testing drugs that target this pathway on different levels, we found a dependence of chordoma cells on JAK2. Because several JAK2 inhibitors are approved, this finding could be immediately applied clinically. This is also supported by the fact that we observed strong interferon signaling in cell lines and patients with chordoma and that JAK2 inhibition with fedratinib inhibited the growth of patient-derived chordoma organoids (*12*). In addition, targeting STAT3 with experimental compounds has been shown to reduce the viability of chordoma cell lines (*60*, *61*). Future studies need to determine why chordoma cells exhibit elevated JAK-STAT signaling and to what extent and how TBXT contributes to this characteristic.

In conclusion, we have developed the first selective TBXT protein binders and provide proof of concept that such compounds can target nuclear proteins. In addition, the T-DARPins have allowed us to map the TBXT regulome, which provides a resource that will inform future fundamental and translational studies and has enabled the discovery of an essential signaling axis that can be targeted with clinically approved drugs and thus may represent an immediately actionable therapeutic opportunity in patients with chordoma. Finally, the T-DARPins themselves could be developed into clinical drugs for chordoma or other TBXT-expressing cancers if delivery to cells in vivo can be achieved.

## MATERIALS AND METHODS

### Generation and verification of T-DARPins

The purification of full-length TBXT and the TBXT DBD is described in the Supplementary Materials.

#### Ribosome display selection

To generate TBXT-specific DARPins, the biotinylated TBXT DBD was immobilized on MyOne T1 streptavidin-coated beads (Pierce) or Sera-Mag neutravidin-coated beads (GE Healthcare) depending on the particular selection round. Ribosome display selections were performed essentially as previously described (*62*), yet using a semiautomatic KingFisher Flex MTP96 well platform. The library included N3C-DARPins with the original randomization strategy as previously reported (*22*) but contained a stabilized C-cap (*63*). In addition, the library consisted of a mixture of DARPins with randomized and nonrandomized N- and C-terminal caps (*18*). Successively enriched pools were cloned as intermediates into a ribosome display-specific vector. Selections were performed over four rounds with decreasing concentrations of the biotinylated TBXT DBD and increasing washing steps, and the third round included a competition with the nonbiotinylated TBXT DBD to enrich binders with high affinities. The final enriched pool of cDNAs encoding putative DARPin binders was cloned into a bacterial pQE30 derivative vector (Qiagen) via unique Bam HI and Hind III sites as a fusion construct with an N-terminal MRGS(H)_8_-tag and a C-terminal Flag-tag containing a T5 lac promoter and *lacI^q^* for expression control. After the transformation of *Escherichia coli* XL1-blue cells, 380 single DARPin clones selected to bind the TBXT DBD were expressed in 96-well format by adding 1 mM IPTG and lysed by adding B-PER reagent plus lysozyme and nuclease (Pierce). After centrifugation, these crude extracts were used for initial screening to bind the TBXT DBD and full-length TBXT using HTRF and ELISA, which are described in detail in the Supplementary Materials.

#### Electrophoretic mobility shift assay

For immobilized metal affinity chromatography (IMAC) purification, 23 identified DARPins were subcloned into a pQIq vector, replacing the MRGS(H)_8_-tag by an MRGS(H)_6_-tag, expressed in small-scale deep-well 96-well plates, lysed with Cell-Lytic B (Sigma), and purified over a 96-well IMAC column (HisPur Cobalt plates, Thermo Fisher Scientific), including washing with high-salt (1 M NaCl) and low-salt (20 mM NaCl) phosphate buffer. Elution was performed with phosphate-buffered saline (PBS) containing 400 mM NaCl and 250 mM imidazole. For the EMSAs, imidazole and NaCl were removed using dialysis devices (3.5K MWCO, Thermo Fisher Scientific). To increase the amount of the 23 candidate DARPins, large-scale expression and purification were performed. DARPins were expressed on a 200-ml scale. After induction with 1 mM IPTG and incubation for 4 hours at 37°C, expression cultures were harvested by centrifugation for 10 min at 4000 rpm. Cell pellets were resuspended in sodium phosphate-based Ni-lysis buffer (PBS, 400 mM NaCl, 20 mM imidazole, and 10% glycerol, pH 7.4), adding Pierce Universal nuclease, and cells were lysed using sonication. DARPins were purified over a HisTrap FF crude column (GE Healthcare) and desalted over a HiTrap 26/10 desalting column (GE Healthcare) using an ÄKTA Pure L1 system (GE Healthcare). EMSAs were performed as follows: 72 pmol TBXT DBD were incubated with 135 pmol IMAC-purified and dialyzed DARPins for 30 min at room temperature in PBS. To analyze binding of the TBXT DBD to DNA, 1.5 pmol of a double-stranded DNA oligonucleotide containing two palindromic T-box binding elements (5′-CATGAAGGATCCATGAA**TTTCACACCT***AGGTGTGAAA*TTGC-3′; IDT) (*11*) and 5′ labels with 6-FAM on both strands was added and incubated for 20 min at room temperature in the dark. Complex formation was analyzed by native 4% acrylamide gel electrophoresis (Mini-gel, Hoefer) using 0.5× TBE as running buffer (50 mM tris hydroxymethyl aminomethane, 50 mM boric acid, and 1 mM EDTA, pH 8.0). Migration of the 6-FAM–labeled oligonucleotide was visualized using a fluorescence imager with excitation at 495 nm and emission at 520 nm.

### Dual-luciferase reporter assay

U-CH2, UM-Chor1, and HEK293T + TBXT cells were transduced with 200 μl of pLenti6.2-V5/DEST DARPin lentivirus. Medium was changed after 24 hours, and cells were seeded after 72 hours. U-CH2 and UM-Chor1 (8000 cells per well) and HEK293T + TBXT (3000 cells per well) were seeded into white, clear bottom 96-well plates, and 24 hours later, cells were transfected with pGL4.34-2X-T-Resp (150 ng per well) and pGL4.73[*hRluc*/SV40] (7.5 ng per well) plasmids using Lipofectamine 3000 (Invitrogen). Nontransfected cells and a single transfection with 150 ng of pGL4.34-2X-T-Resp were included as negative controls. After 48 hours, cells were washed once with PBS and lysed using 20 μl of Passive Lysis Buffer (Promega) with shaking at 1000 rpm for 20 min at room temperature. Measurements were performed with a Victor X3 plate reader (PerkinElmer) following the Dual-Luciferase Reporter Assay System manual (Promega) instructions. *Firefly* and *Renilla* luciferase signals were corrected by subtracting the respective control samples (nontransfected cells for *Firefly* and *Firefly* cross-talk for *Renilla*). The reporter activity was then calculated by dividing the corrected *Firefly* signal by the corrected *Renilla* signal.

### TBXT ChIP-qPCR

ChIP was performed using the iDeal ChIP-seq Kit for Transcription Factors (C01010170, Diagenode) with minor adaptations according to Tosic *et al.* (*64*). In brief, expression of E3_5 or T-DARPins A2, B1, and D4 in UM-Chor1-iDARPin cells was induced for 24 hours with doxycycline (1.5 μg/ml) in tet-free complete media, after which approximately 20 × 10^6^ cells were harvested per ChIP condition and replicate. Cells were double–cross-linked at room temperature in PBS, first with 2 mM disuccinimidyl glutarate (Thermo Fisher Scientific) for 20 min, followed by the addition of 1% formaldehyde (Thermo Fisher Scientific) for 10 min. Cross-linking was quenched with glycine (Diagenode), and cells were lysed on ice according to the manufacturer’s protocol. Chromatin was sheared with a PicoBioruptor (Diagenode) for 10 cycles (30 s on, 30 s off). For each ChIP, 5 μg of TBXT antibody (AF2085, R&D Systems) was bound to 30 μl of magnetic protein A/G beads (88802, Thermo Fisher Scientific). To assess TBXT chromatin occupancy in E3_5 control and T-DARPin–expressing UM-Chor1 cells, qPCR analysis was performed with primers amplifying a negative control region (*KLK3*) and the TBXT binding sites in *KRT8*, *TBXT*, *COL5A2*, *C7orf69*, and *SOX9* previously identified in chordoma (*8*). The qPCR reaction was performed with 1.5 μl of 1:50 diluted ChIP DNA, 3.5 μl of SYBR Green Master Mix (Bio-Rad), and 2 μl of 1.1 μM forward and reverse primers in a 384-well plate (Roche) in a Light Cycler 480 II (Roche). Enrichment of the genomic loci was calculated as a percentage of input chromatin according to the manufacturer’s guidelines.

### Cell culture, vectors, and lentiviral transduction

Detailed information on the culturing of cell lines, the generation of cDNAs and lentiviral vectors, and the lentiviral transduction of cell lines is provided in the Supplementary Materials.

### Preparation of protein lysates

For Western blotting, IGFBP3 ELISA, and AP-MS, cell pellets were washed once in cold PBS, lysed in RIPA buffer (Thermo Fisher Scientific) containing 1× Halt protease inhibitor cocktail (Thermo Fisher Scientific) and 1× Halt phosphatase inhibitor (Thermo Fisher Scientific), and incubated on ice while gently shaking for 30 min. Samples were centrifuged at 14,000*g* for 15 min at 4°C, supernatant was removed, protein concentration was quantified using BCA assay (Pierce), and lysates were stored at −80°C. For DDA-MS, proteins were generated as described above, except cells were harvested by scraping on ice before pelleting. For DIA-MS, proteins were generated as described for DDA-MS with an additional sonication step (BioRupter, Diagenode) for 15 cycles with 30 s on/off before submission.

### Immunoprecipitation

For pulldown of Flag-tagged DARPins from lentivirally transduced U-CH2 and UM-Chor1 cells, 5 mg (AP-MS) or 2 mg (Western blotting) of protein lysate was added to 50 μl of anti-DYKDDDDK (Flag) magnetic agarose beads (prewashed three times, Pierce). After incubation for 1 hour at 4°C on a rotating shaker, the supernatant was discarded, and beads were washed three times for 5 min with PBS containing 0.05% Tween 20. Elution was performed with 80 μl of 3× Flag peptide (1.5 mg/ml in PBS, Sigma-Aldrich) and by incubating for 20 min at room temperature in a ThermoMix Comfort shaker (1400 rpm, Eppendorf). The supernatant was separated from the magnetic beads, and 30 μl of eluate or input lysate was used for Western blotting, or the entire eluate was submitted to the DKFZ Proteomics Core Facility for AP-MS analysis.

For pulldown of TBXT, 275 ng of an anti-TBXT antibody (#81694, Cell Signaling Technology) or 275 ng of a rabbit isotype control antibody (#3900, Cell Signaling Technology) was incubated overnight at 4°C on a rotating shaker with 2.5 mg of UM-Chor1 protein lysate, followed by incubation with 25 μl of protein A/G magnetic beads (prewashed three times, Pierce) for 1 hour at room temperature on a rotating shaker. Afterward, supernatant was discarded, beads were washed three times for 5 min with PBS containing 0.05% Tween 20, and elution was performed in 20 μl of 0.2 M glycine (Thermo Fisher Scientific) by incubation at room temperature in a ThermoMix Comfort shaker (1400 rpm, Eppendorf) for 20 min. The supernatant was separated from the magnetic beads and 5 μl of 1 M tris-HCl (Thermo Fisher Scientific) was added to neutralize the glycine. The eluate was then submitted to the DKFZ Proteomics Core Facility for AP-MS analysis.

For pulldowns from HEK293T cells, these were cotransfected with pShuttle CMV-IRES-GFP encoding a DARPin and pLEX307 containing no cDNA, TBXT-HA, TBXT-R16L-HA, TBXT-H171R-HA, TBXT-G177D-HA, or TBXT-ΔDBD-HA. Cells were harvested by scraping, lysed in IP Lysis Buffer (25 mM tris-HCl, 150 mM NaCl, 1 mM EDTA, 1% Triton X-100, and 5% glycerol, pH 7.4) containing 1× Halt protease and phosphatase inhibitors, and centrifuged at 13,000*g* for 10 min. Protein lysate (1 mg) was added to 30 μl of anti-DYKDDDDK (Flag) magnetic agarose beads (prewashed three times) and incubated overnight at 4°C on a rotating shaker. The supernatant was discarded, and beads were washed four times for 5 min with PBS + 0.05% Tween 20. Elution was performed with 20 μl of 3× Flag peptide (1.5 mg/ml in PBS, Genaxxon BioScience) and by incubating for 20 min at room temperature in a Thermomix shaker (1400 rpm, Eppendorf). The supernatant was separated from the magnetic beads, and 10 μl of eluate or 50 μg of input lysate was used for Western blotting.

### Western blotting

Western blotting was performed using 10 μg of protein lysate (25 μg for detecting DARPins) mixed with 4× Laemmli sample buffer (Bio-Rad) and diluted to 1× with dH_2_O and the addition of β-mercaptoethanol (Sigma-Aldrich) to a final concentration of 10%. The samples were heated to 95°C for 10 min, cooled, and loaded on a 4 to 20% Mini-PROTEAN TGX gel (Bio-Rad). The gels were run for 10 min at 90 V and 45 min at 150 V. Proteins were transferred to PVDF membranes (Bio-Rad) using the Trans-Blot Turbo Transfer System (Bio-Rad) for 30 min. Membranes were blocked for 1 hour at room temperature with 5% milk in TBST (1× tris-buffered saline and 0.1% Tween 20), incubated with primary antibodies overnight at 4°C with gentle rocking, and washed three times for 10 min each in TBST before incubation with a secondary antibody for 1 hour at room temperature. After three additional washing steps in 1× TBST, blots were imaged using the Odyssey CLx (Li-Cor) or the ChemiDoc (Bio-Rad) using SuperSignal West Atto Ultimate Sensitivity Substrate (A3855, Thermo Fisher Scientific). To visualize IGFBP3, membranes were blocked with Intercept TBS buffer (Li-Cor). Primary and secondary antibodies are provided in table S6.

### Structural predictions

The prediction of T-DARPin structures, T-DARPin-TBXT docking, and modeling are described in the Supplementary Materials.

### Mass spectrometry sample preparation, raw data acquisition, and data preprocessing

Information on AP-MS, DDA-MS, and DIA-MS sample preparation, raw data acquisition, and data preprocessing is provided in the Supplementary Materials.

### Mass spectrometry data analysis

#### DDA-MS

Label-free quantification (LFQ) matrices calculated by MaxQuant were imported into R using the DEP package (*65*). Nontargeting control samples (E3_5 DARPin) were used as baselines. First, protein groups were filtered to analyze only those detected at least once for all three replicates in each group (E3_5 and T-DARPin). Next, protein groups with a median LFQ below the 0.05 quantile for all LFQ values (control or treatment) were removed. Then, the filtered LFQ matrix was normalized using variance stabilizing normalization (*66*). Missing values were imputed using the MinDet method as implemented in the imputeLCMD::impute.MinDet function. The minimum was estimated by using the 0.01 quantile. Batch effects were detected via principal components analysis and corrected using the sva::ComBat function (*67*).

#### DIA-MS

The DIA-MS data were processed similarly to the DDA-MS data, except that only proteins detected at least twice in each group were kept, and the LFQ quantile cutoff was reduced to 0.015.

#### Differential protein expression

Protein levels were compared by fitting linear models using the *limma* package in R and a robust empirical Bayes estimation with a variance trend (*68*). The following linear model was used, where DARPin represents a categorical variable encoding the E3_5 or T-DARPin conditions

$${\text{log}}_{2}\text{LFQ}=\beta_{0}+\beta_{1}\text{DARPin}+\epsilon$$ (1)

Differential expression and its statistical significance were calculated by a moderated *t* test. For each comparison of a T-DARPin with the E3_5 condition, proteins with at least 1.5-fold up- or down-regulation and an FDR of less than 5% were selected as significantly deregulated.

#### AP-MS

Data were processed similar to DDA-MS using the control treatment (DARPin E3_5 or isotype antibody) as the baseline for bait treatment (T-DARPins or anti-TBXT antibody). The same linear model (Eq. 1), moderated *t* test, and FDR filter were applied, and T-DARPin binders were then defined as protein groups with a fold change greater than 10 in the bait condition compared to the respective control. Finally, the fold change for each protein group in a bait group was defined as the enrichment of that protein in a given sample. This information is presented along with the corresponding moderated *t* statistics, which indicate the statistical certainty of protein groups as potential T-DARPin binders.

#### Multifactorial linear regression with interactions

DIA-MS data were analyzed separately for the effect of TBXT inhibition in the context of IGFBP3 knockout. First, we merged the datasets for codetected proteins in the sgNTC and sgIGFBP3 conditions. Second, we applied the following model, where *IGFBP3* and *DARPin* are categorical variables encoding the sgNTC/sgIGFBP3 and DARPin E3_5/T-DARPin D4 treatments, respectively, and extracted the effect size and *P* value from the moderated *t* test for the interaction term alone

$$\begin{array}{c} {\text{log}}_{2}\text{LFQ}=\beta_{0}+\beta_{1}\text{IGFBP}3+\beta_{2}\text{DARPin}+\\ \beta_{3}\text{IGFBP}3\cdot \text{DARPin}+\epsilon \end{array}$$ (2)

### RNA preparation

Cell pellets were resuspended with 350 μl of RLT buffer on ice, lysed with a 26-gauge needle, and processed with the RNAeasy Mini Kit (Qiagen), including a DNase I digestion step. Eluted RNA was immediately frozen at −80°C with 1 μl of RNaseOUT (Invitrogen) after quantification with a NanoDrop One system (Thermo Fisher Scientific).

### Quantitative RT-PCR

For each condition, 1 μg of RNA was reverse transcribed using the High-Capacity cDNA Reverse Transcription Kit (Applied Biosystems). cDNA was diluted 1:20 and combined with primers for the target genes and SYBR Green master mix (Bio-Rad). Amplification was performed using standard conditions on a CFX96 Real-Time system (Bio-Rad). Relative mRNA expression was quantified using the 2^−ΔΔCT^ method (*69*) after obtaining CT values from the CFX Manager software (Bio-Rad). Primer sequences are provided in table S7.

### RNA-seq and differential gene expression analysis

UM-Chor1 cells were transduced with pLenti6.2-V5/DEST encoding E3_5 or T-DARPins A2, B1, and D4, each in triplicate, selected with blasticidin (4 μg/ml) for 10 days, and cultured for 2 days without selection before RNA isolation. RNA was quantified and checked for purity on a TapeStation (Agilent). High-quality RNA (RIN > 8.0) was submitted to the DKFZ Next-Generation Sequencing Core Facility, where sequencing libraries were prepared using Illumina TruSeq Stranded Kit and sequenced on an Illumina HiSeq 4000 platform at 100 cycles (paired end). Details on RNA-seq and data preprocessing are provided in the Supplementary Materials.

Differential expression analysis was performed using the DESeq2 package (*70*). Gene-specific read count data were imported into an R environment. Active genes were selected with the zFPKM method using the suggested zFPKM cutoff of −3 (*70*). Biological replicates were analyzed for batch effects using the sva::ComBat function and principal component analysis. The batch term “Replicate” was introduced into the model to adjust for batch effects

$$\text{Expression}=\beta_{0}+\beta_{1}\text{Replicate}+\beta_{2}\text{DARPin}+\varepsilon$$ (3)

Next, differential expression analysis was performed using E3_5 samples as the baseline for all comparisons. The FDR was set to 1% using independent hypothesis weighting (*71*). Misleading fold changes of genes with low expression or high dispersion were adjusted using adaptive empirical Bayes shrinkage (*72*). For each comparison, genes with at least 1.5-fold up- or down-regulation with an FDR less than 1% were selected as significantly regulated.

### Gene set enrichment analysis

#### T-DARPin experiments

Fast preranked GSEA was performed with the *fgsea* package in R (*73*) using the human MSigDB Hallmark, GO_BP version 2023.2.Hs, and Enrichr ARCHS^4^ Tissue gene set collections (*74*, *75*). Genes and proteins were ranked on shrunk log_2_(fold changes) and moderated *t* statistics, respectively. An FDR-adjusted *P* value of 5% was used to infer significantly enriched gene sets. Because of the large number of significantly deregulated gene sets in the GO_BP and ARCHS^4^ Tissue collections, enrichment levels were simplified for representation (*76*). GO_BP terms were clustered based on semantic similarity. ARCHS^4^ Tissue gene sets were clustered based on normalized enrichment scores, but gene set identifiers were simplified in word clouds. Word sizes were determined by the product of the absolute normalized enrichment score and the negative log_10_ of the *P* value.

#### Patient samples

Patients included in this study had been enrolled in the MASTER multicenter precision oncology trial (*43*, *44*), which uses whole-genome/exome sequencing, RNA-seq, and DNA methylation profiling to inform the clinical management of young adults with advanced cancers and with advanced rare cancers regardless of age. All patients or their legal representatives provided written informed consent for banking of tumor and control tissue, molecular analysis, the collection of clinical data, and the publication of molecular and clinical information under a protocol approved by the Ethics Committee of the Medical Faculty of Heidelberg University. The study was conducted in accordance with the Declaration of Helsinki. Gene expression levels normalized as transcripts per million (TPM) for 1107 samples from 1106 patients representing more than 200 cancer types (*43*) were subjected to gene set variation analysis (*77*) using Hallmark gene sets. The minimum size of a resulting gene set after gene identifier mapping to expression data was set to 5 to improve the reliability of the enrichment scores, calculated as the difference between the maximum and minimum deviations of the random walk from the origin using a modified Kuiper statistic. The resulting scores were separated as chordoma (23 samples) and pan-cancer (1084 samples) cohorts. Mean values and 95% confidence intervals were calculated for each Hallmark gene set for each group.

### Integrative analysis of transcriptome and proteome data

#### Definition of commonly deregulated genes and proteins

At the transcript level, commonly deregulated genes were defined by a significant change in the same direction by all three T-DARPin treatments. At the protein level, more permissive criteria were used. We aimed to identify proteins significantly altered in the same direction by at least two T-DARPin treatments, eliminating discordant cases deregulated in one direction by two treatments but in the opposite direction by the third treatment. We defined two mathematical functions to apply this logic. First, we discretized the direction of deregulation into three levels (−1, 0, and 1) for each protein group *g* in each T-DARPin treatment *t* (Eq. 4), where sgn is the signum function, and *f* and *p* represent the log_2_(fold change) and FDR, respectively

dg,t(f,p)≔{sgn(f),if ∣f∣>log2(1.15) and P<0.050,otherwise (4)

Second, we encoded the “commonly deregulated” property as a Boolean value for each protein group using a discretized deregulation function (Eq. 5), where *T* is the set of all T-DARPin treatments

cg(dg,t)≔{TRUE,if ∑t∈Tdg,t>1FALSE,otherwise (5)

#### Data harmonization

DEPs and DEGs were matched by mapping UniProtKB protein identifiers to Ensembl gene identifiers. GENCODE 19 gene names were updated to the GENCODE 45 annotation, originally created for GRCh38 reference chromosomes and mapped back to the GRCh37 primary assembly.

#### Drug targetability

Potentially druggable human genes were previously identified by Jiang *et al.* (*33*). We used table S9 from this publication to annotate our integrated transcriptome and proteome data with target development level and gene family information.

### Spheroid formation assays

The 3D culture of chordoma cells in Matrigel was performed as follows: eight-well glass slides were coated with 60 μl of Matrigel (Corning, Basement Membrane Matrix, LDEV-Free) per well, and 2000 chordoma cells per well were seeded in complete chordoma medium containing 4% Matrigel. The medium was changed once a week, and after 34 (U-CH2) or 60 (U-CH12) days, spheroids bigger than 50 μm were counted with a bright-field microscope.

For single spheroid growth assays, 5000 cells per well of UM-Chor1-iDARPin, UM-Chor1-EV, UM-Chor1-IGFBP3, U-CH2-EV, U-CH2-IGFBP3, or UM-Chor1-Cas9 transduced with sgNTC, sgTBXT, or sgIGFBP3 were seeded in an ultralow attachment 96-well plate (Corning) and allowed to aggregate into one spheroid per well for 7 to 10 days. Half-medium exchanges were performed one to two times a week. DARPin, IGFBP3, or empty vector (EV) expression was induced using doxycycline (0.5 μg/ml) or sterile dH_2_O as a negative control. Spheroids were imaged every 7 days using a Lionheart FX automated microscope (BioTek) in 96-well acquisition format. Guiding beacons were set for each well, and spheroid images were acquired at fourfold magnification using bright-field illumination (LED 7, integration time 220, gain 1). A 10-step (250 μm per step) z-stack was acquired for each spheroid and compressed into a projection image (z-proj). Z-proj images were imported into FIJI version 1.53C, and the spheroid area was calculated with a customized FIJI macro and reported as μm^2^ × 10^5^. To measure their viability, spheroids were transferred using wide-bore pipette tips to white opaque 96-well plates (Corning), and CellTiter-Glo 3D reagent assay (Promega) was used following the manufacturer’s instructions. Luminescence was acquired with an Envision Multimode plate reader (PerkinElmer), and raw units (×10^7^) or raw units normalized to EV controls were reported.

### Cell viability, cell cycle, and apoptosis analysis

The quantification of viable cells by trypan blue staining and MTS assay and the analysis of cell cycle and apoptosis by flow cytometry are described in the Supplementary Materials.

### Mouse experiments

NOD.Cg-*Prkdc^scid^ Il2rg^tm1Wjl^*/SzJ (NSG) mice were housed in the DKFZ Center for Preclinical Research. All animal procedures were approved by the regional authority in Karlsruhe, Germany (reference number 35-9185.81/G-51/21) and performed according to federal and institutional guidelines. U-CH1 cells (2 × 10^5^ per well) were seeded in six-well plates, spin infected with 10 μl of concentrated pLenti6.2 E3_5 or D4 lentivirus per well after 3 days, selected with blasticidin (6 μg/ml) for 1 week, and allowed to recover in selection-free media for 4 days (fig. S3G). U-CH1 cells expressing E3_5 or D4 were harvested, washed, and resuspended in DPBS. A total of 1 × 10^6^ cells in 55 μl of DPBS were mixed with 55 μl of Matrigel (Corning, Growth Factor Reduced Basement Membrane Matrix, LDEV-Free), of which 100 μl was injected subcutaneously into the flanks of isoflurane-anesthetized, female, 6- to 8-week-old NSG mice provided by the DKFZ Center for Preclinical Research. Each cell line was applied to both flanks of three mice, resulting in six E3_5 tumors and six D4 tumors. The animals were monitored closely, and the width and length of the tumors were measured with a caliper. The tumor volume was calculated using the formula (length × width × width)/2. The mice were euthanized after reaching the permitted criteria of a maximum tumor size of 2 cm in one diameter and a maximum tumor volume of 1.5 cm^3^, and tumors were processed for histopathologic, protein, and RNA analyses.

### Immunofluorescence

Immunofluorescence analyses of UM-Chor1 cells and spheroids are described in the Supplementary Materials.

### IGFBP3 protein analyses

Information on the quantification of IGFBP3 using ELISA and the analysis of IGFBP3 glycosylation and secretion is provided in the Supplementary Materials.

### Drug treatment of cell lines

Fedratinib (#HY-10409, MedChemExpress), AZD1480 (#SML1505, Sigma), fludarabine (#F9813, Sigma), filgotinib (#HY-18300, MedChemExpress), gilteritinib (#S7754, Selleckchem), pacritinib (#S8057, Selleckchem), and afatinib (#SML3073, Sigma) were dissolved to 10 mM in sterile DMSO (Sigma) and stored at −80°C. Before use, compounds were redissolved for 10 min at 37°C, vortexed, and inspected for the absence of precipitates. Compounds were never subjected to multiple freeze-thaws. A total of 1000 cells per well were plated in technical and biological triplicate into collagen-coated white clear-bottom 96-well plates and allowed to adhere for 24 hours before a 7-day drug treatment. For the initial drug screen, compounds were serially diluted from 10 μM to 1 nM. A T_0_ plate for calculating the GR was measured using CellTiter-Glo (Promega) following the manufacturer’s instructions. After 7 days, plates were measured using CellTiter-Glo (Promega) following the manufacturer’s instructions. Luminescence was acquired using an Envision Multimode plate reader (PerkinElmer). GR values were obtained with grcalculator.org (*49*) and graphed using GraphPad Prism. Colony formation assays (14 days, 1 μM drug) in 6-well plates were performed as described previously (*78*) using normal growth medium and an initial seeding density of 10,000 cells per well for U2OS, and 25,000 cells per well for U-CH1, U-CH2, UM-Chor1, and JHC7 cells. For apoptosis measurements, cells were treated with 3 μM drug for 4 or 7 days. Information on the flow cytometry–based assays is provided in the Supplementary Materials.

### Statistical analysis

Statistical analyses were performed with GraphPad Prism version 10.2.2 or R version 4.3.2. For the analysis of experiments, averages and SEMs were generated from biological replicates, and statistical significance was determined using one-way analysis of variance (ANOVA) and the post hoc Dunnett’s test for multiple comparisons or unpaired *t* test. DEGs were identified via fitting genewise generalized linear models and subsequently using the Wald test to compare the model coefficients, as part of the DESeq2 workflow (*70*). DEPs were identified via empirical Bayes-moderated *t* test using the limma workflow (*68*).

## Supplementary Materials

### The PDF file includes:

Supplementary Text Figs. S1 to S6 Tables S1 to S7 Legends for datasets S1 to S7 References

### Other Supplementary Material for this manuscript includes the following:

Datasets S1 to S7
