# Supplementary material for: Selective targeting of TBXT with DARPins identifies regulatory networks and therapeutic vulnerabilities in chordoma
Source: Sci Adv. 2025 Sep 3;11(36):eadu2796. doi: 10.1126/sciadv.adu2796 (PMC12407079; doi:10.1126/sciadv.adu2796)
Supplement: Supplementary file 1 — Supplementary Text Figs. S1 to S6 Tables S1 to S7 Legends for datasets S1 to S7 References [file sciadv.adu2796_sm.pdf]

Science Advances
AAAS

Supplementary Materials for

Selective targeting of TBXT with DARPins identifies regulatory networks and therapeutic vulnerabilities in chordoma

Charles S. Umbaugh *et al.*

Corresponding author: Claudia Scholl, claudia.scholl@nct-heidelberg.de;
Stefan Fröhling, stefan.froehling@nct-heidelberg.de

Sci. Adv. **11**, eadu2796 (2025)
DOI: 10.1126/sciadv.adu2796

The PDF file includes:

Supplementary Text
Figs. S1 to S6
Tables S1 to S7
Legends for datasets S1 to S7
References

Other Supplementary Material for this manuscript includes the following:

Datasets S1 to S7

Supplementary Text

Generation and verification of T-DARPins

Purification of full-length TBXT and the TBXT DNA-binding domain (sequences given below): The purification of the biotinylated recombinant TBXT DBD for ribosome display selection and full-length TBXT for verification after selection was performed by the European Molecular Biology Laboratory Protein Expression and Purification Core Facility, Heidelberg, Germany. Briefly, *E. coli* BL21(DE3) cells were transformed with the pET20b-A(H6)-AviTag plasmid encoding codon-optimized His6-tagged full-length TBXT or the TBXT DBD (amino acids 41–224; sequences provided below). 10 mL overnight culture were added to 1 liter LB broth supplemented with 100 µg/mL carbenicillin. The cultures were grown at 37°C until the optical density at 600 nm reached approximately 0.6. The cultures were then cooled down to 18°C for 30 minutes, expression of TBXT was induced with 0.2 mM IPTG, and cultures were grown overnight at 18°C. Cells were harvested by centrifugation, and the pellets were stored at −20°C. After thawing, the pellets were resuspended in running buffer (50 mM Tris pH 8.0, 250 mM NaCl, 20 mM imidazole) supplemented with 5 mM $MgCl_2$, 10 µg/mL DNAse, 0.5 mg/mL lysozyme, and cOmplete EDTA-free protease inhibitors (Roche). The cells were lysed by two passages through a Microfluidizer. After centrifugation, the supernatant was loaded onto a 5 mL Protino Ni-NTA column (Macherey-Nagel) at 4°C. The Ni-NTA column was washed with running buffer until the A280 nm signal returned to baseline, and the column was eluted with running buffer containing 500 mM imidazole. The elution fractions with full-length TBXT or the TBXT DBD were pooled and dialyzed overnight at 4°C to 50 mM Tris pH 7.0, 50 mM NaCl. The next day, the dialyzed sample was loaded onto a 5 mL HiTrap SP column (Cytiva) pre-equilibrated with 50 mM Tris pH 7.0 and 50 mM NaCl. After washing, the HiTrap SP column was eluted in a gradient to 50 mM Tris pH 7.5 and 1 M NaCl over 12 column volumes. The elution fractions were pooled, concentrated, and injected onto a HiLoad 16/600 Superdex 75 pg column (Cytiva) pre-equilibrated with 20 mM Tris pH 7.5, 300 mM NaCl, 10% glycerol, and 0.1 mM TCEP. The purest protein fractions were pooled, aliquoted, flash-frozen in liquid nitrogen, and stored at −80°C. The identity of the purified protein samples was confirmed by mass spectrometry analysis.

HTRF: Binding of Flag-tagged DARPins to the streptavidin-immobilized, biotinylated TBXT DBD or full-length TBXT was measured using FRET (donor: Streptavidin-Tb cryptate [610SATLB, Cisbio], acceptor: mAb anti-FLAG M2-d2 [61FG2DLB, Cisbio]). Further, HTRF measurements against 'No Target' allowed for discrimination of TBXT-specific hits. Experiments were performed at room temperature in white 384-well Optiplate plates (PerkinElmer) using the Taglite assay buffer (Cisbio) at a final volume of 20 µL per well. FRET signals were recorded after 30 minutes using a Varioskan LUX Multimode Microplate (Thermo Scientific). HTRF signals were obtained by dividing the acceptor signal (665 nm) by the donor signal (620 nm) to derive the 665/620 ratio. The background signal was determined using reagents in the absence of DARPins.

ELISA: ELISAs were performed using streptavidin-coated 384-well plates and used for immobilization of the biotinylated TBXT DBD or full-length TBXT at a concentration of 50 nM. Detection of DARPins (1:1,000 dilution of crude extracts) was performed by using a mouse-anti-FLAG M2 monoclonal antibody (dilution 1:5,000; Sigma, F1804) as primary antibody and a goat-anti-mouse antibody conjugated to an alkaline phosphatase (dilution 1:10,000; Sigma, A3562) as secondary antibody. After addition of para-nitrophenyl phosphate, absorbance at 405 nm was determined after 30 minutes. Signals at A540 nm were subtracted as background correction.

Sequences of full-length TBXT and the TBXT DBD

Codon-optimized full-length TBXT (5’ to 3’):

ATGGCA***CATCACCATCACCATCAC***GGAAGCGGC*GGCCTGAACGATATTTTTGAAGCGCAGAAAA*
*TTGAATGGCATGAA*GGATCCATGAGCAGTCCGGGTACAGAAAGCGCAGGTAAAAGCCTGCAGTA
TCGTGTTGATCATCTGCTGAGCGCAGTTGAAAATGAACTGCAGGCAGGTAGCGAAAAAGGTGAT
CCGACCGAACGTGAACTGCGTGTTGGTCTGGAAGAAAGCGAACTGTGGCTGCGTTTTAAAGAAC
TGACCAATGAAATGATCGTGACCAAAAATGGTCGTCGCATGTTTCCGGTTCTGAAAGTTAATGT
TAGCGGTCTGGACCCGAATGCAATGTATAGCTTTCTGCTGGATTTTGTGGCAGCAGATAATCAC
CGTTGGAAATATGTTAATGGTGAATGGGTTCCTGGTGGTAAACCGGAACCGCAGGCACCGAGCT
GTGTTTATATTCATCCGGATAGCCCGAATTTTGGTGCACATTGGATGAAAGCACCGGTTAGCTT
TAGCAAAGTGAAACTGACGAATAAACTGAATGGTGGTGGTCAGATTATGCTGAATAGCCTGCAT
AAATATGAACCGCGTATTCATATTGTTCGTGTTGGT***GGT***CCGCAGCGTATGATTACCAGCCATT
GTTTTCCAGAAACACAGTTTATTGCAGTTACCGCCTATCAGAACGAAGAAATTACCGCACTGAA
AATCAAATATAACCCGTTTGCAAAAGCCTTCCTGGATGCAAAAGAACGTAGCGATCATAAAGAA
ATGATGGAAGAACCGGGTGATAGCCAGCAGCCTGGTTATAGCCAGTGGGGTTGGCTGCTGCCTG
GTACAAGCACCCTGTGTCCGCCTGCAAATCCGCATCCGCAGTTTGGTGGTGCACTGAGCCTGCC
GAGCACACATAGCTGTGATCGTTATCCGACACTGCGTAGCCATCGTAGCAGCCCGTATCCGAGT
CCGTATGCACATCGTAATAATAGCCCGACCTATAGCGATAATAGTCCGGCATGTCTGAGCATGC
TGCAGTCACATGATAATTGGAGTAGCCTGGGTATGCCTGCACATCCGAGTATGCTGCCGGTTAG
CCATAATGCAAGCCCTCCGACCAGCAGCAGTCAGTATCCGAGCCTGTGGTCAGTTAGCAATGGT
GCAGTTACACCGGGTAGCCAGGCAGCCGCAGTTTCAAATGGTCTGGGTGCACAGTTTTTTCGTG
GTTCACCGGCACATTACACACCGCTGACACATCCGGTTAGCGCACCGAGCAGCAGCGGTAGTCC
GCTGTATGAAGGTGCAGCAGCAGCAACCGATATTGTGGATAGCCAGTATGATGCAGCAGCCCAG
GGTCGTCTGATTGCAAGCTGGACACCGGTTTCACCGCCTAGCATGTAATAA

Key for the marked sequence parts:

6x-His tag; *AviTag*; codon-optimized TBXT sequence; ***wildtype G177 codon***

Codon-optimized TBXT DBD (5’ to 3’):

ATGGCA***CATCACCATCACCATCAC***GGAAGCGGC*GGCCTGAACGATATTTTTGAAGCGCAGAAAA*
*TTGAATGGCATGAA*GGATCCGAACTGCGTGTTGGTCTGGAAGAAAGCGAACTGTGGCTGCGTTT
TAAAGAACTGACCAATGAAATGATCGTGACCAAAAATGGTCGTCGCATGTTTCCGGTTCTGAAA
GTTAATGTTAGCGGTCTGGACCCGAATGCAATGTATAGCTTTCTGCTGGATTTTGTGGCAGCAG

ATAATCACCGTTGGAAATATGTTAATGGTGAATGGGTTCCTGGTGGTAAACCGGAACCGCAGGC
ACCGAGCTGTGTTTATATTCATCCGGATAGCCCGAATTTTGGTGCACATTGGATGAAAGCACCG
GTTAGCTTTAGCAAAGTGAAACTGACGAATAAACTGAATGGTGGTGGTCAGATTATGCTGAATA
GCCTGCATAAATATGAACCGCGTATTCATATTGTTCGTGTTGGT***GGT***CCGCAGCGTATGATTAC
CAGCCATTGTTTTCCAGAAACACAGTTTATTGCAGTTACCGCCTATCAGAACGAAGAAATTACC
GCACTGAAAATCAAATATAACTAA

Key for the marked sequence parts:
6x-His tag; *AviTag*; codon optimized DBD sequence; ***wildtype G177 codon***

Sequences of the three lead T-DARPins

The T-DARPins are protected by a patent (please see Competing Interests paragraph in the main text). In addition to the following sequences, the DARPins have an RGS-His-tag at the N-terminus (MRGSHHHHHHHH) and a FLAG-Tag at the C-terminus (DYKDDDDK).

T-DARPin A2 (N to C-terminus):
GSDLGKKLLEAARAGQDDEVRILMANGADVNAFDMFGETPLHLAAQEGHLEIVEVLLK
TGADVNAKDMWGITPLHLAALRGHLEIVEVLLKAGADVNAHDKAGHTPLHLAAWNG
HLEIVEVLLKHGADVNAQDKFGKTPFDLAIDNGNEDIAEVLQKAAKLN

T-DARPin B1 (5' to 3'):
GSDLGKKLLEAARAGQDDEVRILMANGADVNATDWNGVTPLHLAAHQGHLEIVEVLL
KTGADVNAHDIQGQTPLHLAALTGHLEIVEVLLKHGADVNAQDAAGMTPFDLAATFG
NEDIAEVLQKAAKLN

T-DARPin D4 (5' to 3'):
GSDLGKKLLEAARAGQDDEVRILMANGADVNAYDFRGQTPLHLAATMGHLEIVEVLL
KHGADVNADDLVGWTPLHLAALFGHLEIVEVLLKAGADVNAWDIHGWTPLHLAAAEG
HLEIVEVLLKTGADVNAQDTSGATPFDLAAHQGNEDIAEVLQKAAKLN

Cell culture

UM-Chor1 (kindly provided by the Chordoma Foundation) was maintained in IMDM:RPMI-1640 (4:1, Gibco) with 10% FBS (Sigma-Aldrich), 1% penicillin/streptomycin (P/S, Gibco), and 1x non-essential amino acids (NEAA, Sigma-Aldrich). U-CH1, U-CH2, and U-CH12 (kindly provided by Silke Brüderlein and Peter Möller) were maintained in IMDM:RPMI-1640 (4:1) with 10% FBS, 1% P/S, and 1% L-glutamine (Gibco) on collagen I-coated tissue culture flasks (Corning), and JHC7 (kindly provided by the Chordoma Foundation) in DMEM-F12 (Gibco) with 10% FBS and 1% P/S on collagen I-coated tissue culture flasks. U2OS (kindly provided by Karsten Rippe) and HT-1080 (kindly provided by Marcel Trautmann) were maintained in DMEM (Gibco) with 10% FBS and 1% P/S. MEC-1 (kindly provided by Sascha Dietrich) and Jurkat (kindly

provided by Thomas Mercher) were maintained in RPMI with 10% FBS and 1% P/S. HCT-116 (kindly provided by Levi Garraway) and MES-SA (*79*) were cultured in McCoy's 5a, 10% FBS, and P/S. For experiments, all chordoma cell lines were cultured on collagen I-coated flasks. For UM-Chor1-iDARPin cells, FBS was replaced with tet-free FBS (Takara), and DARPins were induced by the addition of 0.5 µg/mL doxycycline (Th. Geyer) or sterile H_2O as negative control. HEK293T cells were cultured in DMEM with 10% FBS and 1% P/S. All cell lines were cultured under standard conditions (37°C, 5% CO_2) and routinely tested for the absence of mycoplasma. Cell line identity was verified using the Multiplex Cell Authentication Test (Multiplexion) or the Human Cell Line Authentication Service (EurofinsGenomics Germany).

Vectors

DARPins (containing an N-terminal MRGS$(H)_6$-tag and a C-terminal Flag-tag) were cloned from the pQIq backbone into the pDONR221 entry vector (Invitrogen) and transferred to the pLenti6.2-V5/DEST lentiviral expression vector (with blasticidin or hygromycin resistance, Invitrogen) and the pLenti CMVtight Puro DEST inducible vector using Gateway Technology (Invitrogen). pLenti CMV rtTA3 Blast (w756-1), pLenti CMV Hygro DEST (w117-1), and pLenti CMVtight Puro DEST were a gift from Eric Campeau (Addgene plasmids #26429, #17454, and #26430). For transient expression in HEK293T cells, DARPins were cloned into the pShuttle CMV-IRES-GFP vector (*55*) using *SnaBI* and *NotI* restriction enzymes (NEB).

For dual-luciferase reporter assays, the pGL4.34[*luc2P*/SRF-RE/Hygro] vector (Promega, kindly provided by Thordur Oskarsson) encoding the *Firefly* luciferase gene under a minimal promoter was used. To create a TBXT-responsive reporter vector, the serum response factor element was replaced by a DNA sequence containing two palindromic TBXT response elements (2X-T-Resp) (*9*) using the *Acc65I* and *HindIII*-HF restriction enzymes (NEB) to generate the pGL4.34-2X-T-Resp vector. The 2X-T-Resp forward and reverse oligonucleotides with *Acc65I* and *HindIII* sticky overhangs were synthesized at Sigma-Aldrich with the following sequences (TBXT binding sites underlined): 5'-AGC TAA TTT CAC ACC TAG GTG TGA AAT TCC CGG GAA TTT CAC ACC TAG GTG TGA AAT T-3' (forward) and 5'- GTA CAA TTT CAC ACC TAG GTG TGA AAT TCC CGG GAA TTT CAC ACC TAG GTG TGA AAT T-3' (reverse). The pGL4.73[*hRluc*/SV40] control vector for expression of the *Renilla* luciferase gene was obtained from Promega.

For immunoprecipitation of TBXT variants, the codon-optimized full-length TBXT cDNA with or without a C-terminal HA-tag was synthesized (IDT) and cloned into pLenti6.2-V5/DEST using Gateway Technology. The TBXT-HA cDNAs containing the variants R16L, H171R, G177D, and ΔDBD (removal of amino acids 42–219) were generated in pDONR221 using the QuikChange II Site-Directed Mutagenesis Kit (Agilent). The primer sequences are provided in **Table S1**. For

transient expression in HEK293T cells, the cDNAs were transferred into the pLEX307 vector (gift from David Root, Addgene plasmid #41392) using Gateway Technology.

For CRISPR/Cas9-mediated knockout, lentiCas9-Blast for Cas9 expression (gift from Feng Zhang, Addgene plasmid #52962) and lentiGuide-Puro (gift from Feng Zhang, Addgene plasmid #52963) and lentiGuide-Hygro (gift from Caroline Goujon, Addgene plasmid #139462) for sgRNA expression were used. The sgRNA sequences (**Table S2**) were cloned into the vectors with the *BbsI* restriction enzyme (NEB).

For miR-E-based gene knockdown, we used the SGEP vector (gift from Johannes Zuber, Addgene plasmid #111170). The unmodified SGEP vector contains an shRNA targeting *Renilla* luciferase and was used as a negative control (shLuc). The shRNAs sequences (**Table S3**) were cloned into SGEP as PCR amplified 97-mer oligos (*80*) using *XhoI* and *EcoRI* restriction enzymes (NEB). For shRNA based gene knockdown, we used the pRSI12 vector as described (*81*) with sequences provided in **Table S4**.

Lentivirus production and transduction of cells

Lentivirus was produced by transfection of HEK293T cells with envelope (pMD2.G), packaging (psPAX2), and expression plasmids. pMD2.G and psPAX2 were a gift from Didier Trono (Addgene plasmids #12259 and #12260). The plasmid mix was combined with Opti-MEM (Gibco) and TransIT-LT1 (Mirusbio) transfection reagent and incubated overnight in a 10 cm dish of 70% confluent HEK293T cells. Transfection media was replaced with 3 to 4.5 mL DMEM with 30% FBS and harvested after 24 and 48 hours. Virus-containing medium was passed through a 0.45 μm polyethersulfone membrane filter (PES, Pall) and stored at −80°C or concentrated using PEG-8000 (40 % W/V, VWR International) with 1.2 M NaCl and frozen at −80°C in 1x PBS (Gibco).

For transduction, attached cells in six-well plates were spin-infected for two hours (30°C, 2,000 rpm) in culture medium containing 300 μL unconcentrated virus or 10 μL concentrated virus per well and 4 μg/mL polybrene (Merck Millipore). After overnight incubation, virus-containing medium was removed, cells were trypsinized and washed, plated into cell culture flasks, and, depending on the vector used, subjected to selection with predetermined cell line-specific concentrations of blasticidin, puromycin, or hygromycin for at least four days before the start of experiments. UM-Chor1, JHC7, U-CH1, and U-CH2 cells were transduced with pLenti6.2-V5/DEST encoding E3_5 or T-DARPins and selected with blasticidin. The HEK293T+TBXT cell line was generated with pLenti6.2-V5/DEST encoding TBXT and selected with blasticidin. The UM-Chor1-iDARPin cell lines were generated by transducing cells with pLenti CMV rtTA3 Blast (w756-1), and after selection with blasticidin, the cells were transduced with pLenti CMVtight Puro DEST (w768-1) encoding E3_5 or the T-DARPins and selected with puromycin and blasticidin. For knockout experiments, UM-Chor1-Cas9 and U-CH2-Cas9 cells were generated

with lentiCas9-Blast virus and selected with blasticidin. These Cas9-expressing cells were transduced with lentiGuide-Puro-sgRNA virus and selected with puromycin and blasticidin to express single sgRNAs. For combined IGFBP3 knockout and TBXT inhibition, UM-Chor1-Cas9 cells stably expressing sgNTC or sgIGFBP3 were transduced with pLenti CMV Hygro DEST (w117-1) encoding E3_5 or T-DARPin D4 and selected with hygromycin, blasticidin, and puromycin. For IGFBP3 and TBXT double-knockout, UM-Chor1-Cas9 stably expressing sgNTC and sgIGFBP3 were transduced with lentiGuide-Hygro containing sgNTC and sgTBXT and selected with hygromycin, blasticidin, and puromycin. For knockdown experiments, UM-Chor1 or U-CH2 cells were transduced with either pRSI12 containing shNTC or shYAP or SGEP containing shLuc or shTBXT and selected with puromycin.

Mass spectrometry sample preparation, data acquisition, and preprocessing

AP-MS: The eluate (35 µl) of each IP sample was run for 0.5 cm into an SDS-PAGE and the entire piece was cut out and digested with trypsin according to Shevchenko et al. (*82*) adapted to a DigestPro MSi robotic system (INTAVIS Bioanalytical Instruments AG). The LC-MS/MS analysis was carried out on an Ultimate 3000 UPLC system (Thermo Fisher Scientific) directly connected to a Q-Exactive HF-X Orbitrap mass spectrometer for a total of 90 minutes. Peptides were online desalted on a trapping cartridge (Acclaim PepMap300 C18, 5 µm, 300 Å wide pore; Thermo Fisher Scientific) for three minutes using a 30 µL/minute flow of 0.05% TFA in water. The analytical multistep gradient (300 nL/minute) was performed using a nanoEase MZ Peptide analytical column (300 Å, 1.7 µm, 75 µm x 200 mm, Waters) using solvent A (0.1% formic acid in water) and solvent B (0.1% formic acid in 80% acetonitrile with 20% water). For 50 minutes the concentration of B was linearly ramped from 2% to 25% and to 40% within the next 10 minutes, followed by a quick ramp to 95%, after five minutes the concentration of B was lowered to 2% and a 20 minute equilibration step appended. Eluting peptides were analyzed in the mass spectrometer using data dependent acquisition (DDA) mode. A full scan at 120k resolution (375-1500 m/z, 3e6 AGC target, 54 ms maxIT) was followed by up to 10 MS/MS scans. Peptide features were isolated with a window of 1.6 m/z, fragmented using 27% NCE. Fragment spectra were recorded at 30k resolution (1.5e5 AGC target, 54 ms maxIT). Unassigned and singly charged eluting features were excluded from fragmentation and dynamic exclusion was set to 10 seconds. Each sample was followed by a wash run (60 minutes) to minimize carry-over between samples. Instrument performance throughout the course of the measurement was monitored by regular (approximately one per 48 hours) injections of a standard sample and an in-house shiny application. Data preprocessing was carried out by MaxQuant (2.1.4.0) (*83*) using an organism specific database extracted from Uniprot.org (human reference database with one protein sequence per gene, containing 20,591 unique entries from March 1st, 2023 and nine DARPin sequences). Settings were left as default with the following adaptions: Match between runs (MBR) was enabled to transfer peptide identifications across RAW files based on accurate retention time and m/z. Fractions were set in a way that MBR was only performed within replicates. Label free

quantification (LFQ) was enabled with default settings. The iBAQ-value (*84*) generation was enabled.

DDA-MS: Proteins (10 µg) were run for 0.5 cm into an SDS-PAGE and the entire piece was cut out and digested using trypsin according to Shevchenko et al. (*82*) adapted to a DigestPro MSi robotic system (INTAVIS Bioanalytical Instruments AG). The LC-MS/MS analysis was carried out on an Ultimate 3000 UPLC system (Thermo Fisher Scientific) directly connected to an Orbitrap Exploris 480 mass spectrometer for a total of 150 minutes. Peptides were online desalted on a trapping cartridge (Acclaim PepMap300 C18, 5 µm, 300 Å wide pore; Thermo Fisher Scientific) for three minutes using 30 µL/minute flow of 0.05% TFA in water. The analytical multistep gradient (300 nL/minute) was performed using a nanoEase MZ Peptide analytical column (300 Å, 1.7 µm, 75 µm x 200 mm, Waters) using solvent A (0.1% formic acid in water) and solvent B (0.1% formic acid in acetonitrile). For 132 minutes the concentration of B was linearly ramped from 4% to 30%, followed by a quick ramp to 80%, after two minutes the concentration of B was lowered to 2% and a 10 minute equilibration step appended. Eluting peptides were analyzed in the mass spectrometer using DDA mode. A full scan at 120k resolution (380-1400 m/z, 300% AGC target, 45 ms maxIT) was followed by up to two seconds of MS/MS scans. Peptide features were isolated with a window of 1.4 m/z, fragmented using 26% NCE. Fragment spectra were recorded at 15k resolution (100% AGC target, 54 ms maxIT). Unassigned and singly charged eluting features were excluded from fragmentation and dynamic exclusion was set to 35 seconds. Each sample was followed by a wash run (40 minutes) to minimize carry-over between samples. Instrument performance throughout the course of the measurement was monitored by regular (approximately one per 48 hours) injections of a standard sample and an in-house shiny application. Data preprocessing was carried out by MaxQuant (2.1.4.0) (*83*) using an organism specific database extracted from Uniprot.org (human reference database with one protein sequence per gene, containing 79,038 unique entries from January 3rd, 2022 and four DARPin sequences). Settings were left at default with the following adaptions: MBR was enabled to transfer peptide identifications across RAW files based on accurate retention time and m/z. Fractions were set in a way that MBR was only performed within replicates. LFQ was enabled with default settings. Separate parameter groups were assigned for DARPin (E3_5, A2, B1, D4) and TBXT knock-out (sgNTC, sgTBXT) samples, including separate LFQ normalization. The iBAQ-value (*84*) generation was enabled.

DIA-MS: Proteins (10 µg) were digested with trypsin using an AssayMAP Bravo liquid handling system (Agilent technologies) running the autoSP3 protocol according to Müller et al. (*85*). The LC-MS/MS analysis was carried out on an Ultimate 3000 UPLC system (Thermo Fisher Scientific) directly connected to an Orbitrap Exploris 480 mass spectrometer for a total of 120 minutes. Peptides were online desalted on a trapping cartridge (Acclaim PepMap300 C18, 5 µm, 300 Å wide pore; Thermo Fisher Scientific) for three minutes using 30 µL/minute flow of 0.05% TFA in water. The analytical multistep gradient (300 nL/minute) was performed using a nanoEase MZ

Peptide analytical column (300 Å, 1.7 µm, 75 µm x 200 mm, Waters) using solvent A (0.1% formic acid in water) and solvent B (0.1% formic acid in acetonitrile). For 102 minutes the concentration of B was linearly ramped from 4% to 30%, followed by a quick ramp to 78%, after two minutes the concentration of B was lowered to 2% and a 10 minute equilibration step appended. Eluting peptides were analyzed in the mass spectrometer using data independent acquisition (DIA) mode. A full scan at 120k resolution (380-1400 m/z, 300% AGC target, 45 ms maxIT) was followed by data independent MS2 acquisition covering a mass range of 400-1000 m/z via 47 DIA windows of variable width with one Da overlap and 28% NCE. Fragment spectra were recorded at a resolution of 30k (1000% AGC target, 54 ms maxIT). Each sample was followed by a wash run (40 minutes) to minimize carry-over between samples. Instrument performance throughout the course of the measurement was monitored by regular (approximately one per 48 hours) injections of a standard sample and an in-house shiny application. Preprocessing of DIA RAW files was performed with Spectronaut (Biognosys, version 17.1.221229.55965) in directDIA+ (deep) library-free mode. Default settings were applied with the following adaptions: Within the Pulsar Search in Peptides, the Max Peptide Length was set to 35, in Result Filters, the Peptide Charge was enabled and the Max Charge set to 6 and the Min Charge set to 2. Within DIA Analysis under Identification the Precursor PEP, Cutoff was set to 0.01, the Protein Qvalue Cutoff (Run) set to 0.01, and the Protein PEP Cutoff set to 0.05. In Quantification, the Proteotypicity Filter was set to Only Protein Group Specific, the Quantification window was set to Not Synchronized, and the Major Group Quantity was set to Sum peptide quantity. The data was searched against the human proteome from Uniprot (human reference database with one protein sequence per gene, containing 20,591 unique entries from March 1st, 2023), the contaminants FASTA from MaxQuant (246 unique entries from December 22nd, 2022) and the sequences for E3_5 and D4.

Structural predictions for T-DARPins and TBXT

Using recently published structures (*17*), we modeled the binding of the T-DARPins to WT TBXT *in silico* using PyMOL (Version 3.1.0), AlphaFold2 (AF2) (*86*) via the COSMIC2 web server (*87*), and the HADDOCK 2.4 web server (*25*) for molecular docking efforts. T-DARPin structures were generated using AF2. For visualization of molecules, PyMOL was used. The E3_5 structure was obtained from PDB (ID: 1MJ0) (*88*). For docking, we obtained the ground-state model for WT TBXT from PDB (ID: 7HI8) (*17*) and removed water and cadmium ions using PyMOL. Subsequently, we used PDBTools on the HADDOCK server to remove alternative residue conformations. Finally, we uploaded the .pdb files for 7HI8 and the best AF2 structure for each T-DARPin to HADDOCK and performed protein-protein docking using the default settings on the server, which included the following important features. We designated active residues on T-DARPins using the sequence analyses performed previously (*89*) and assigned active residues on T-DARPins using the pockets A, B, C, and D recently identified (*17*). Then, we overlaid the best cluster T-DARPin:TBXT DBD (7HI8) docking pose for each T-DARPin against one another for further analysis.

RT-PCR

For the amplification of DARPins from xenografted tumors, mRNA was purified from the U-CH1 tumors using a Qiagen RNeasy Mini kit. 500 ng RNA was reverse transcribed using the High-Capacity cDNA Reverse Transcription Kit (Applied Biosystems). The cDNA was diluted into 100 µL of nuclease free water. 10 ng of cDNA was amplified in a 50 µL reaction with 0.5 µL Velocity polymerase (Meridian Bioscience, #BIO-21098), 10 µL Hi-Fi Buffer, 1 µL dNTPs (Qiagen, #201912), 1 µL DMSO, 1 µL forward and reverse primer, and amplified using the following program: 98°C – 3 mins, 98°C – 20s / 68°C – 30s / 72°C – 1 min for 30 cycles, 72°C – 5 min. The primer sequences were as follows: DARPin-forward 5'-ATGGGCATGAGAGGATCGC-3' and DARPin-reverse 5'-TGTCGTCGTCATCCTTGTAGT-3'.

RNA-seq data preprocessing

Reads were processed using the RNA-seq workflow 1.3.0 developed by the DKFZ Omics IT and Data Management Core Facility (https://github.com/DKFZ-ODCF/RNAseqWorkflow). First, FASTQ reads were aligned to the 1,000 Genomes Project Phase II Reference Genome (hs37d5) based on NCBI GRCh37 via two-pass alignment using STAR 2.5.3a (*90*). The STAR index was generated from the 1,000 Genomes assembly and GENCODE Version 19 gene models with a sjdbOverhang of 200. Alignment call parameters were as follows:

**Parameter**	**Value**
--alignIntronMax	1100000
--alignIntronMin	20
--alignMatesGapMax	1100000
--alignSJstitchMismatchNMax	5 -1 5 5
--alignSJDBoverhangMin	3
--chimJunctionOverhangMin	15
--chimScoreMin	1
--chimScoreJunctionNonGTAG	0
--chimSegmentMin	15
--chimSegmentReadGapMax	3
--clip3pAdapterSeq	AGATCGGAAGAGCACACGTCTGAACTCCAGTCA
--genomeLoad	NoSharedMemory
--limitBAMsortRAM	100000000000
--outBAMsortingThreadN	1
--outSAMstrandField	intronMotif
--outSAMtype	BAM Unsorted SortedByCoordinate
--outSAMunmapped	Within KeepPairs
--outFilterMismatchNmax	5

--outFilterMismatchNoverLmax	0.3
--outFilterMultimapNmax	1
--readFilesCommand	gunzip -c
--runThreadN	8
--sjdbOverhang	200
--twopass1readsN	-1
--twopassMode	Basic

Duplicate marking of the resultant main alignment file was done with sambamba 0.6.5 (*91*). The chimeric file was sorted using samtools 1.6 (*92*). BAM indexes were generated using sambamba 0.6.5. Quality control analysis was performed using samtools flagstat and the rnaseqc tool version 1.1.8 (*93*). Depth of coverage analysis for rnaseqc was turned off. Gene-specific read counting was performed using featureCounts version 1.5.1 (*94*) over exon features based on the GENCODE 19 gene models. Only unique alignments were counted. Strand specific counting was also used. A custom script was used to calculate RPKM and TPM expression values. For total library abundance calculations, all genes on chromosomes X, Y, MT as well as rRNA and tRNA genes were omitted, as they are likely to introduce library size estimation biases.

Determination of viable cell numbers

To assess the effect of DARPin expression on chordoma cell number, 30,000 cells per well were seeded in six-well plates, the medium was changed once a week, and cells were detached and counted on day 21 using a Vi-CELL counter (Beckman) and trypan blue exclusion.

To assess the effect of DARPin expression on cell proliferation, 3,000 (HCT-116 and HT-1080), or 1,500 (MES-SA) cells per well were seeded in 96-well plates and cell viability was assessed after different incubation times with the CellTiter 96® AQueous One Solution Cell Proliferation Assay (Promega) according the manufacturer's instructions. Absorbance at 490 nm was determined with an iMark Microplate Reader.

Cell cycle analysis

Twelve days after transduction, 0.5 to 1 x 10^6 UM-Chor-1 cells were trypsinized, washed using 1 mL of cold PBS containing 0.1% BSA, and centrifuged with 200 x g for five minutes at 4°C. Cell pellets were resuspended in 100 µL cold PBS/0.1% BSA and fixed by drop-wise adding 900 µL of ice-cold 80% ethanol. Cells were stored at −20°C for a minimum of six hours, washed with 1 mL cold PBS/0.1% BSA, and centrifuged with 200 x g for 10 minutes at 4°C. After two washing steps, the cells were resuspended in 500 µL PBS containing 50 µg/mL PI (Invitrogen) and 200 µg/mL RNAse A (Qiagen) and were incubated for 30 minutes at 37°C. The analysis was performed by flow cytometry using a FACSCelesta instrument (BD Biosciences), and the acquired data were analyzed using FlowJo (BD Biosciences).

Apoptosis analysis

UM-Chor1, JHC7, U-CH2, and U-CH12 cells were transduced with DARPins, selected, and reseeded without selection antibiotic after four days. After six days, i.e., ten days after transduction, adherent and floating cells were harvested, washed in PBS, and stained with annexin V and 7-AAD (FITC Annexin V Apoptosis Detection Kit I, BD Biosciences) according to the manufacturer's instructions. Cells were analyzed by flow cytometry using an LSR Fortessa instrument (BD Biosciences). For the assessment of drug sensitivity, chordoma (2 x 10^5) or U2OS (1 x 10^5) cells were seeded in collagen-coated t25 flasks and 24 hours later were incubated with DMSO or 3 µM drug for 4 or 7 days. Adherent and floating cells were harvested, washed in PBS, and stained with annexin V (Invitrogen, #R37174) and 7-AAD (BD Biosciences, #559925) and analyzed by flow cytometry using a FACSCelesta instrument (BD Biosciences) after four or seven days of treatment. Flow cytometry data were analyzed with FlowJo (version 10.10).

Immunofluorescence

UM-Chor1 cells were analyzed 10 days after infection in 12-well collagen-coated plates (Corning). The medium was removed and cells were washed with cold DPBS and fixed with the Image-iT Fixation/Permeabilization Kit according to manufacturer's instructions (Invitrogen, #R37602). Cells were stained with mouse Penta-His Alexa Fluor 488 antibody (1:250 in 1x DPBS/3% BSA, #35310, Qiagen) for one hour at room temperature, washed three times with 1x DPBS, and stained for one hour with DAPI (0.125 µg/mL) and Texas Red-X Phalloidin (1:400, #T7471, Invitrogen) in 1x DPBS/3% BSA. Cells were washed three times with 1x DPBS and then imaged using a Lionheart FX automated microscope (BioTek). Spheroids were processed for confocal microscopy using a protocol modified from (*95*). Briefly, spheroids were centifuged at 50 g for 3 minutes, and after removing the medium, were transferred to 1% BSA pre-coated 2 mL round bottom tubes. They were washed two times with cold 1x DPBS/1% BSA, fixed at 4°C using 1x DPBS/4% PFA for 45 minutes, permeabilized at 4° using cold 1x DPBS/0.1% Tween-20 for 10 minutes, blocked at 4°C using 1x DPBS/0.1% Triton/0.2% BSA for 15 minutes, and then transferred to a 24-well plate (precoated with 1% BSA). The spheroids were incubated with the primary antibodies mouse anti-Ki-67 (1:200 in 1x DPBS/0.1% Triton/0.2% BSA, #9449, Cell Signaling Technology) or rabbit anti-TBXT (1:200 in 1x DPBS/0.1% Triton/0.2% BSA, #81694, Cell Signaling Technology) overnight at 4°C with gentle rocking. They were then washed 3 times with 1x DPBS/0.1% Triton/0.2% BSA for 2 hours, stained with the secondary antibodies goat anti-rabbit IgG 488 (1:1,000 in 1x DPBS/0.1% Triton/0.2% BSA, #A32731, Invitrogen) or goat anti-mouse IgG 488 (1:1,000 in 1x DPBS/0.1% Triton/0.2% BSA, #A32723, Invitrogen) overnight at 4°C with gentle rocking, were then washed 3 times with 1x DPBS/0.1% Triton/0.2% BSA for 2 hours, incubated with DAPI (0.125 µg/mL) and Texas Red-X Phalloidin (1:400) in 1x DPBS/0.1% Triton/0.2% BSA for 30 minutes, washed 3 times in 1x DPBS/0.1% Triton/0.2% BSA (10 minutes), and then incubated in clearing solution (60% glucose, 2M fructose) for 20 minutes prior to acquisition. Spheroids were imaged using an A1R MP confocal microscope (Nikon).

IGFBP3 ELISA

10 µg chordoma cell lysate and a serial dilution of recombinant IGFBP3 protein (0.1 µg/mL to 74.97 pg/mL, Millipore) to create a standard curve for quantification was incubated overnight in an ELISA plate coated with anti-human IGFBP3 antibody (Millipore, RAB0235) at 4°C. The ELISA and quantification were performed according to the manufacturer's instructions. Absorbance was recorded at 450 nm using a Victor X3 plate reader (Perkin Elmer).

IGFBP3 glycosylation and secretion analysis

UM-Chor1-Cas9 and U-CH2-Cas9 cells were transduced with sgNTC or sgTBXT lentivirus, selected for 14 days, and serum starved for 72 hours. Protein lysate was prepared as described above. Conditioned medium (CM; 11 mL per t75 flask) was collected, centrifuged with 300 g at 4°C for five minutes to remove cell debris, and stored at −80°C. To isolate secreted proteins, we used an adapted protocol employing trichloroacetic acid (TCA, SERVA Electrophoresis) and sodium deoxycholate (DOC, #D6750, Sigma-Aldrich) (*96*). CM was thawed on ice, mixed with DOC to 1% (v/v), incubated on ice for 30 minutes, mixed with TCA to 7.5% (v/v), and incubated on ice for 60 minutes. The processed CM was transferred to 1.5 mL tubes and centrifuged with 15,000 g at 4°C for 20 minutes. The supernatant was discarded, and protein pellets were washed with 1 mL ice-cold acetone, gently vortexed, and placed at −20°C for five minutes. The tubes were centrifuged with 15,000 g at 4°C for 20 minutes, the supernatant was discarded, and the washing step was repeated. After final supernatant removal, cleaned pellets were air-dried in a chemical hood for 30 minutes and stored at −80°C. For the PNGase F digestion, two pellets per condition were dissolved in a total of 50 µL 1x PNGase F buffer (Thermo Fisher) and incubated for three hours at 4° in a tube shaker (750 rpm, Eppendorf) with mixing by pipette every 30 minutes. Residual TCA was neutralized using 5 µL 1M Tris-HCL (pH 8), and samples were frozen at −80°. PNGase F digestion was performed according to the manufacturer's instructions with a few minor modifications using a PNGase F Glycan Cleavage Kit (Thermo Fisher). For total cell lysate digestion, 25 µg protein per condition were mixed with 4 µL 10x PNGase F buffer and 0.5 µL PNGase F and adjusted to a total volume of 40 µL with HPLC-grade water. The reaction was incubated for one hour at 50°C to digest glycosylated residues. For undigested control samples, PNGase F was omitted. For CM samples, equal volumes of isolated protein were digested to normalize across conditions. Therefore, 10 µL of CM-isolated protein was mixed with 4 µL 10x PNGase F buffer and 0.5 µL PNGase F and adjusted to a total volume of 40 µL with HPLC-grade water. For subsequent SDS-PAGE and western blotting, total cell lysate samples were normalized by mass (10 µg/sample), and CM samples were normalized by volume (33.75 µL of reaction) before loading.

Supplementary Figure 1

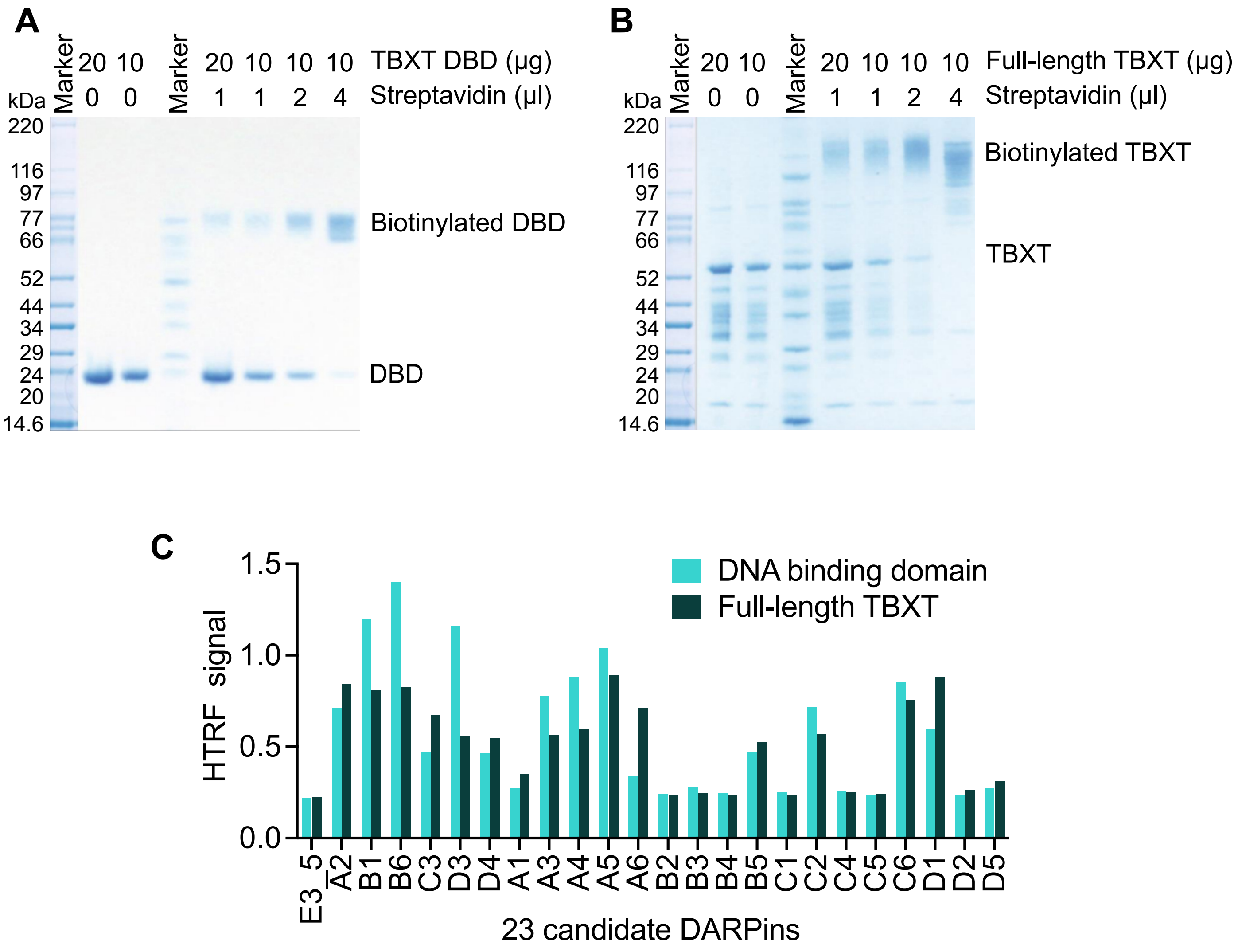

Fig. S1. Generation of TBXT-binding DARPins. (**A, B**) Coomassie blue-stained SDS-PAGE gels demonstrating biotinylation of the purified recombinant TBXT DBD (A) and full-length TBXT (B) used for the selection of TBXT-binding DARPins. The TBXT DBD was used as the target protein for ribosome display, and full-length TBXT was used in addition to the DBD for the following selection and verification experiments. (**C**) HTRF signal for binding of the non-targeting control DARPin E3_5 and the 23 candidate DARPins to the TBXT DBD and full-length TBXT.

Supplementary Figure 2

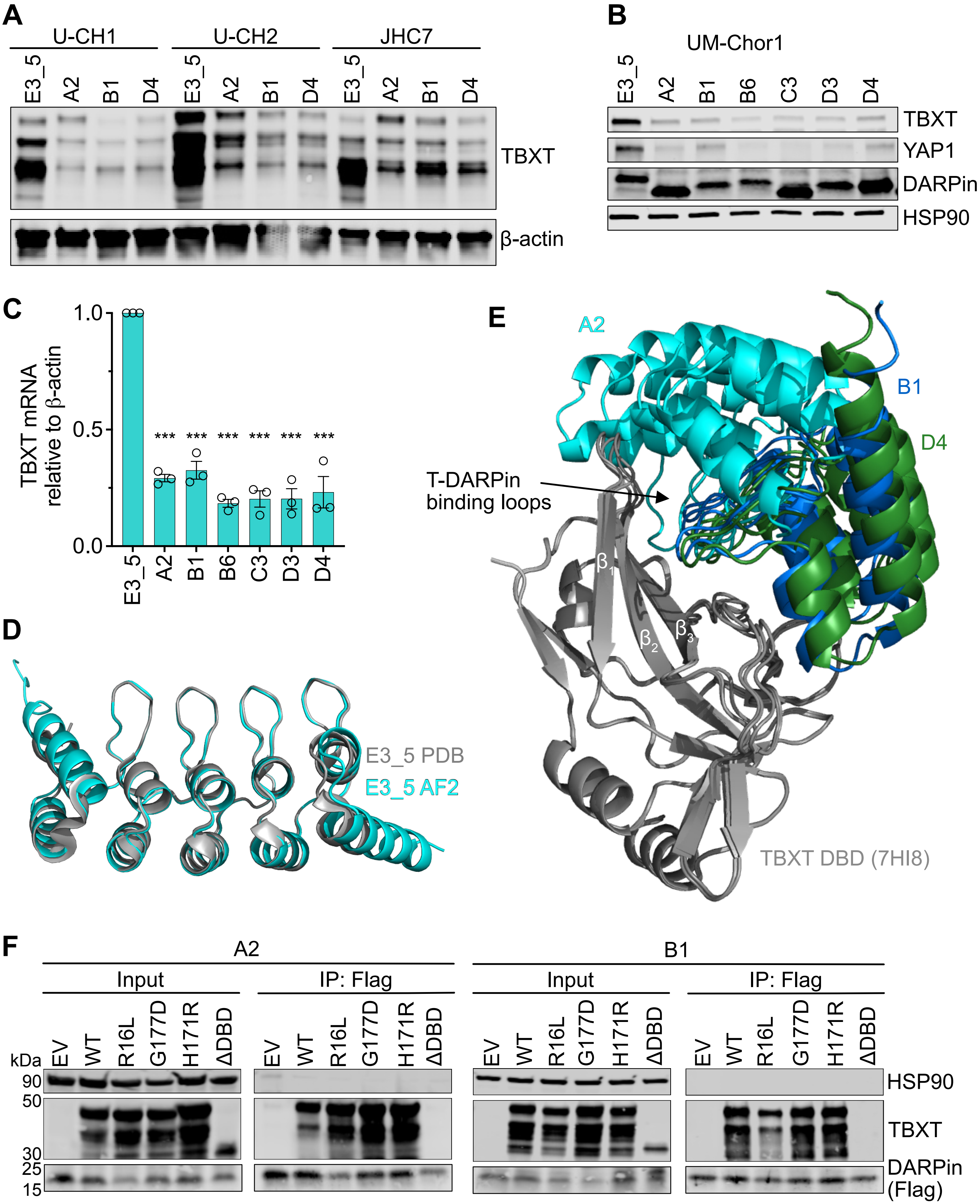

Fig. S2. Specificity and activity of T-DARPins. (**A**) Western blot of U-CH1, U-CH2, and JHC7 chordoma cell lines 12 days after transduction with the three indicated T-DARPins or E3_5. One representative blot of three independent experiments is shown. (**B**) Western blot of UM-Chor1 cells 12 days after transduction with the six lead T-DARPins or E3_5 with antibodies detecting TBXT and the TBXT downstream effector YAP1. One representative blot of three independent experiments is shown. (**C**) *TBXT* mRNA expression measured by quantitative RT-PCR in UM-Chor1 cells stably expressing the six lead T-DARPins or E3_5. One-way ANOVA with Dunnett's test for multiple comparisons; mean ± SEM of three biological replicates. *$p \leq 0.05$, **$p \leq 0.01$, ***$p \leq 0.001$. (**D**) Overlay of the PDB structure M1J0 for DARPin E3_5 (gray) with an AlphaFold2-generated E3_5 structure (teal) using the same sequence. (**E**) Interaction modeling using HADDOCK 2.4 of T-DARPin structures A2 (teal), B1 (blue), and D4 (dark green) generated with AlphaFold2 with the TBXT DBD structure PDB 7HI8 (gray). (**F**) Anti-Flag bead pulldowns of T-DARPins A2 and B1 co-transfected into HEK293T cells with EV or a vector expressing HA-tagged WT TBXT, TBXT-R16L, TBXT-G177D, TBXT-H171R, or TBXT lacking the DBD (ΔDBD). This figure is related to Figure 2D.

Supplementary Figure 3

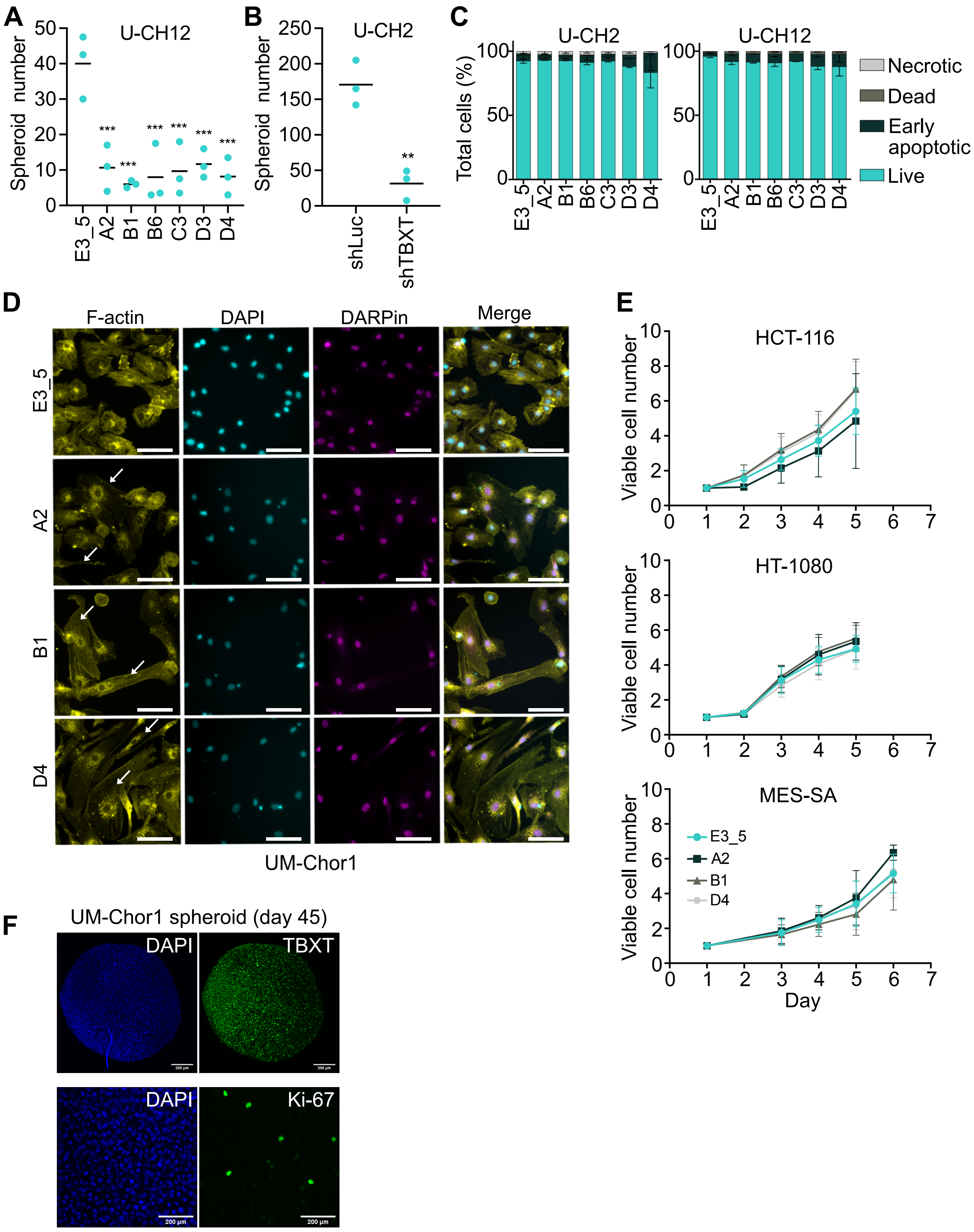

Supplementary Figure 3 continued

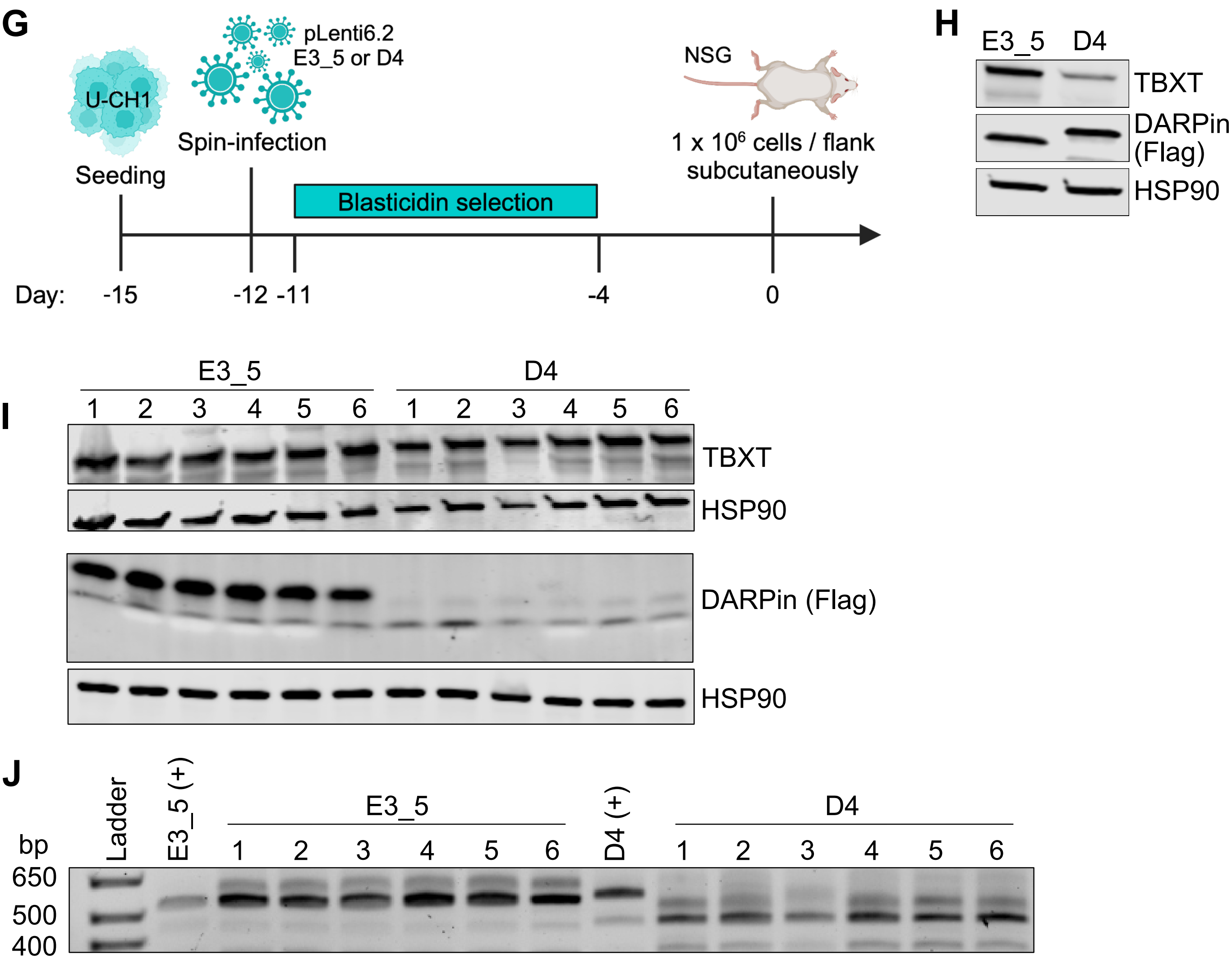

Fig. S3. Cellular effects of T-DARPin expression. (**A**) 3D Matrigel cultures of U-CH12 cells. Spheroids were counted on day 60 after lentiviral transduction with the indicated DARPins. One-way ANOVA with Dunnett's test for multiple comparisons; mean of three biological replicates. $^*p \leq 0.05$, $^{**}p \leq 0.01$, $^{***}p \leq 0.001$. (**B**) Same experimental setup as in (A) but with lentiviral transduction of an shRNA targeting TBXT or shLuc. Unpaired t-test; $^{**}p \leq 0.01$. (**C**) Apoptosis measured by flow cytometry after staining with annexin V and PI 10 days after lentiviral transduction of U-CH2 and U-CH12 cells with the indicated DARPins. Mean ± SEM of two biological replicates. Necrotic, PI^+/annexinV^-; early apoptotic, PI^-/annexin V^+; dead, PI^+/annexin V^+; live, PI^-/annexin V^-. (**D**) Immunofluorescence of UM-Chor1 cells 10 days after transduction with DARPins A2, B1, D4, or E3_5. Cells were stained with F-actin-specific phalloidin (yellow), nucleus-specific DAPI (teal), and DARPin-specific anti-His (magenta) antibodies. The original images were pseudo-colored for better visualization. White arrows depict pancake-like and spindle-shaped cells. Scale bar, 100 µm. (**E**) Number of viable cells measured by MTS assay relative to day 1 over time of the TBXT-negative cell lines HCT116 (colon cancer), HT-1080 (fibrosarcoma), and MES-SA (uterine sarcoma) expressing DARPins E3_5, A2, B1, or D4. (**F**) Confocal microscopy images of UM-Chor1 spheroids grown for 45 days in ultra-low attachment plates and stained with anti-TBXT and anti-Ki-67 antibodies and DAPI. (**G**) Schematic of the timeline of the preparation of U-CH1 cells before injection into NSG mice. Created in BioRender. Fröhling, S. (2024) BioRender.com/c53k096. (**H**) Western blot of U-CH1 cells transduced with E3_5 and T-DARPin D4 immediately before injection into NSG mice. (**I**) Western blot of lysates from 12 U-CH1 tumors (six transduced with E3_5 and six transduced with T-DARPin D4) at the endpoint of the

xenotransplantation experiment. (**J**) PCR from mRNA of 12 U-CH1 tumors (six transduced with E3_5 and six transduced with T-DARPin D4) at the endpoint of the xenotransplantation experiment with primers for amplifying the DARPin mRNAs. The positive controls E3_5 (+) and D4 (+) were amplified from DARPin mRNA extracted from transduced U-CH1 cells immediately before implantation.

Supplementary Figure 4

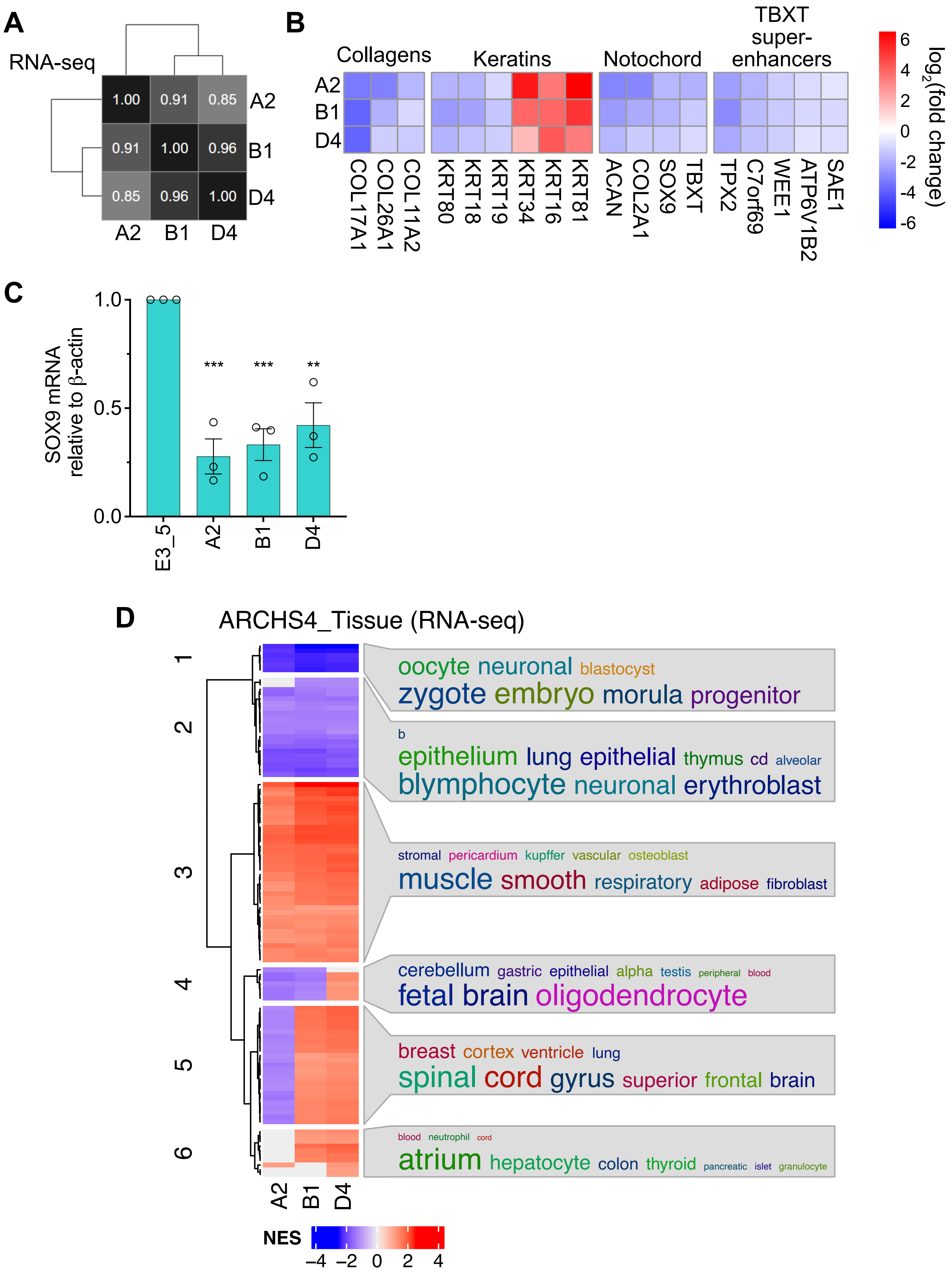

Supplementary Figure 4 continued

E

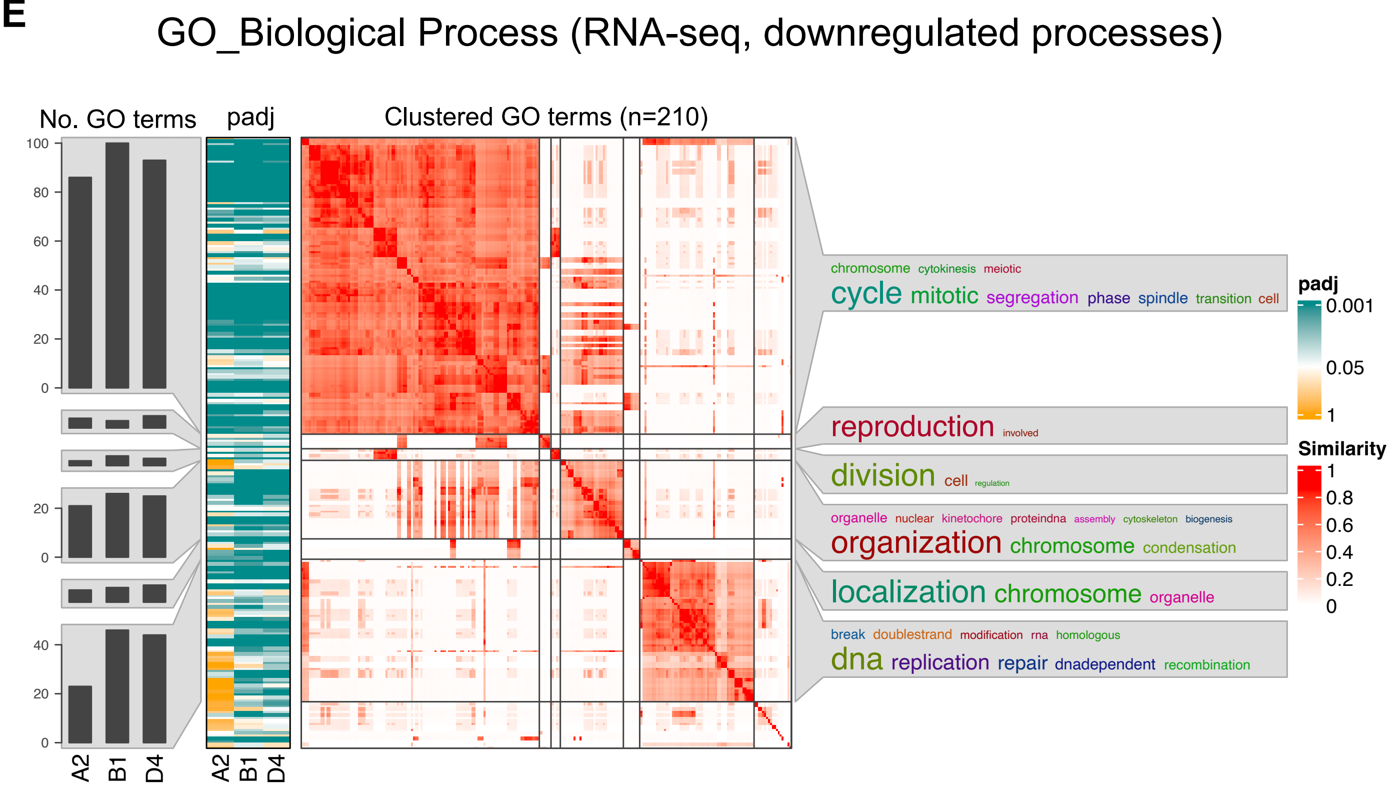

F

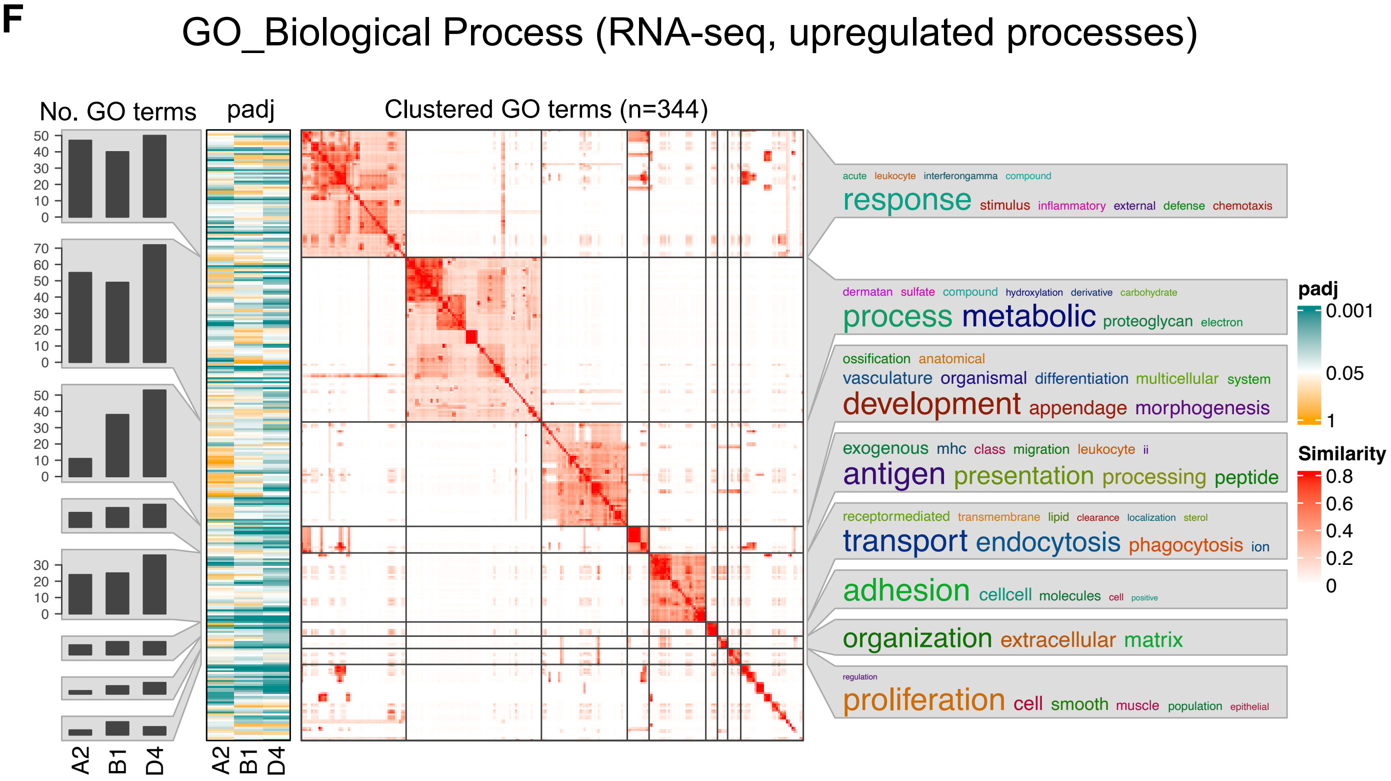

Supplementary Figure 4 continued

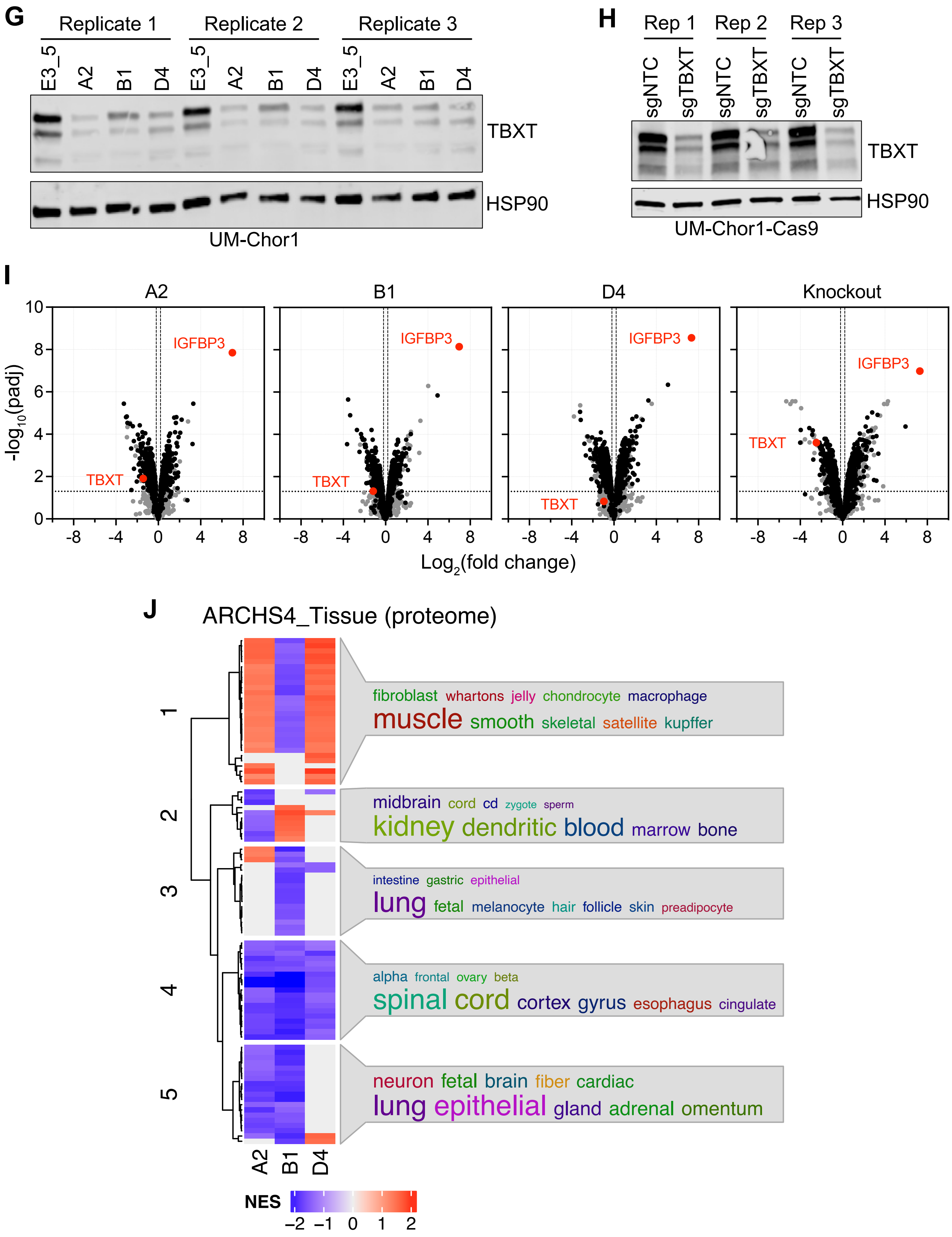

Supplementary Figure 4 continued

K

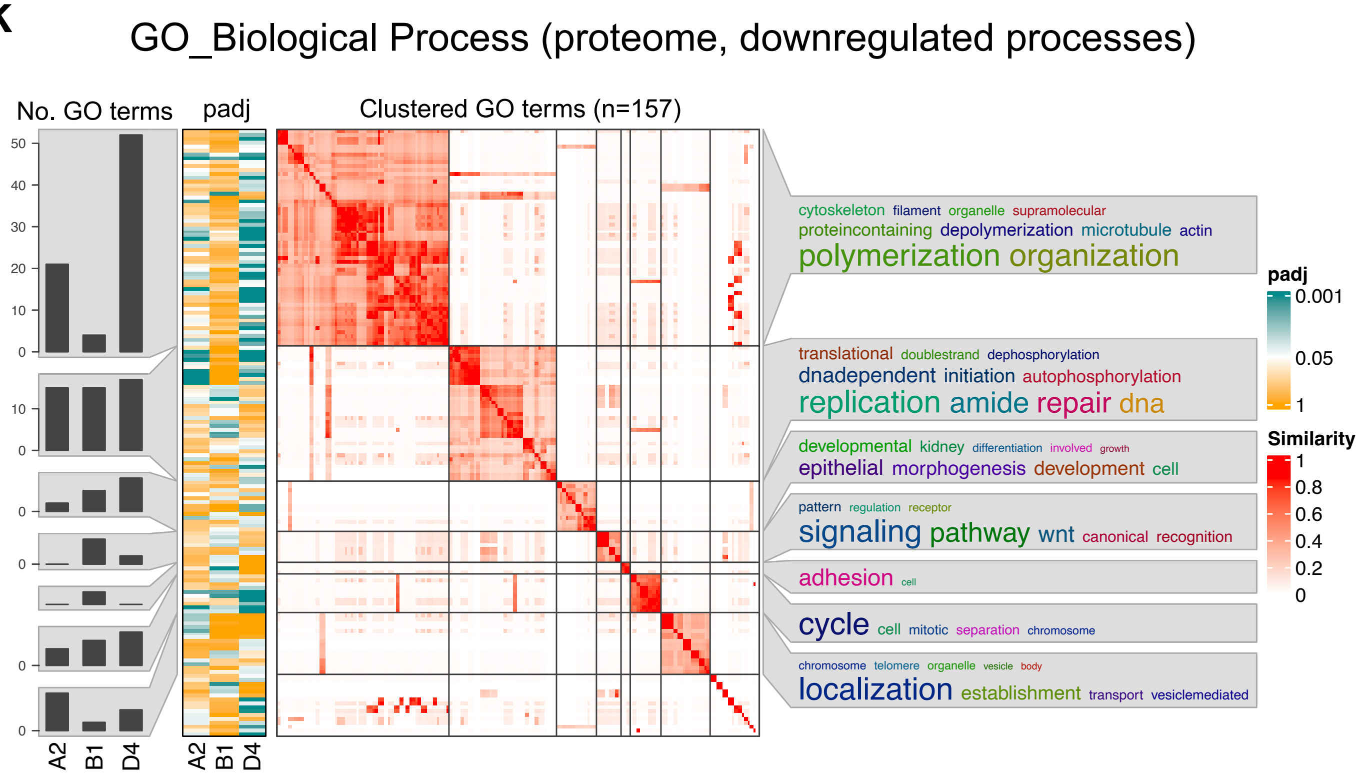

L

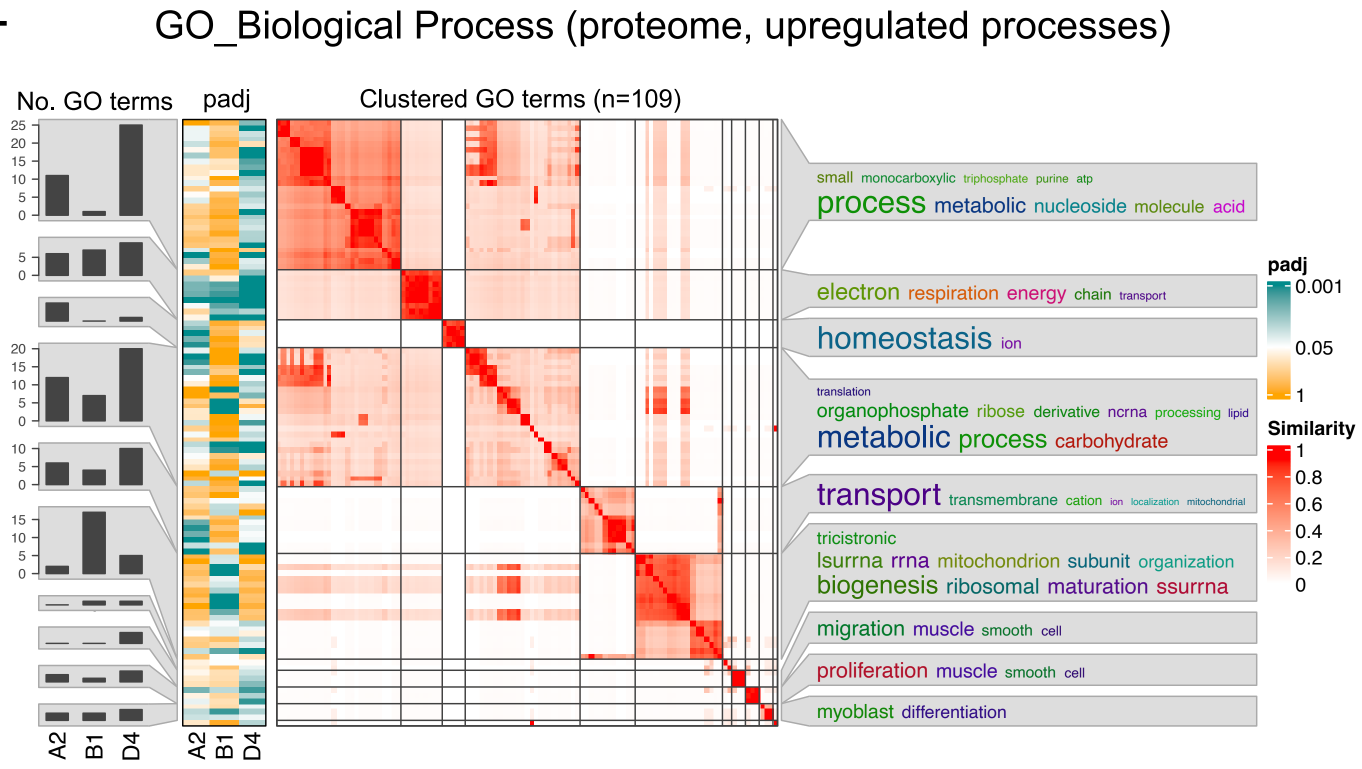

Supplementary Figure 4 continued

M

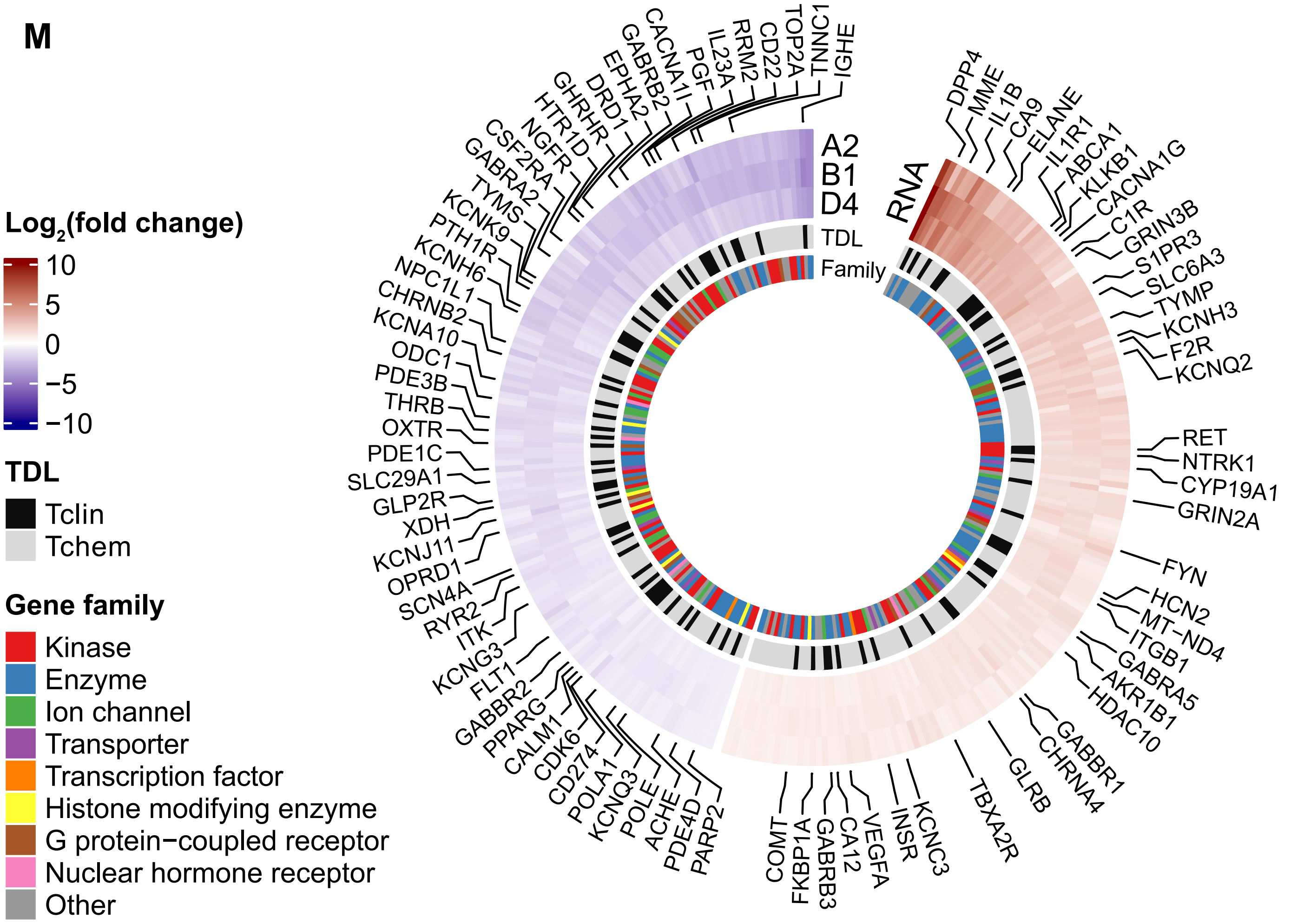

Fig. S4. TBXT regulome determined by T-DARPins in UM-Chor1 cells. (**A**) Pearson correlation coefficient matrix for pairwise similarity of differential expression levels (log_2(fold change), generalized linear model (GLM) with a Wald test, FDR < 1%) in the context of three T-DARPin conditions. RNA-seq of UM-Chor1 cells 12 days after transduction with T-DARPins A2, B1, and D4 and E3_5 performed in biological triplicates. Differential expression was calculated using the DESeq2 workflow comparing gene expression in the context of each T-DARPin treatment to E3_5. (**B**) Heatmap of RNA-seq data depicting genes known to be regulated by TBXT or chordoma-relevant genes. Log_2(fold change) was calculated as in A. (**C**) *SOX9* mRNA expression measured by quantitative RT-PCR in UM-Chor1 cells stably expressing the three T-DARPins or E3_5. One-way ANOVA with Dunnett's test for multiple comparisons; mean + SEM of three biological replicates. *p ≤ 0.05, **p ≤ 0.01, ***p ≤ 0.001. (**D**) Fast preranked GSEA of RNA-seq data with the ARCHS^4 Tissue gene collection. Gene set names were separated into words and represented in word clouds based on the frequency of occurrence of each word. Common words such as "cell" or "tissue" were omitted. NES, normalized enrichment score. FDR < 5% via permutation test followed by Benjamini-Hochberg correction. (**E**) Fast preranked GSEA of RNA-seq data with the GO_BP gene collection focused on downregulated pathways. The left panel shows the number of significant GO terms (adjusted p-value [padj] < 0.05 via permutation test followed by Benjamini-Hochberg correction) in each cluster for each T-DARPin (column); the middle panel indicates the padj as a heatmap for each GO term (row) and T-DARPin (column); and the heatmap in the right panel shows the semantic similarity clustering of GO terms by '"binary_cut". The number of GO terms clustered (n = 210) is shown above this heatmap. The enriched GO terms are summarized per cluster as word cloud by frequency of occurrence as in panel (D). (**F**) Fast preranked GSEA of RNA-seq data with the GO_BP gene collection focused on upregulated pathways. Please see panel (E) for a description. (**G, H**) Western blot of UM-Chor1 cell lysates prepared in triplicate for DDA-MS**.** Cells were transduced 14 days earlier with T-DARPins A2, B1, or D4 or E3_5 (G) or with sgNTC or sgTBXT

(H). (**I**) Volcano plots of proteins detected by DDA-MS in UM-Chor1 protein lysates prepared in biological triplicate as shown in (G) and (H). Differential protein expression changes were calculated relative to the respective negative control (E3_5 for T-DARPins and sgNTC for sgTBXT) via permutation test followed by Benjamini-Hochberg correction. (**J**) Fast preranked GSEA of proteome data with the ARCHS[4] Tissue gene collection. Please see panel (D) for a description. (**K**) Fast preranked GSEA of proteome data with the GO_BP gene collection focused on downregulated pathways. Please see panel (E) for a description. (**L**) Fast preranked GSEA of proteome data with the GO_BP gene collection focused on upregulated pathways. Please see panel (E) for a description. (**M**) Circus plot with DEGs categorized according to their target development levels (TDL). The heatmap shows the log_2(fold change) of DEGs detected by RNA-seq via GLM with a Wald test, FDR < 5% via Benjamini-Hochberg correction. DEGs with Tclin level are written out. DEGs also detected by DDA-MS are not shown here but are included in Fig. 4E.

Supplementary Figure 5

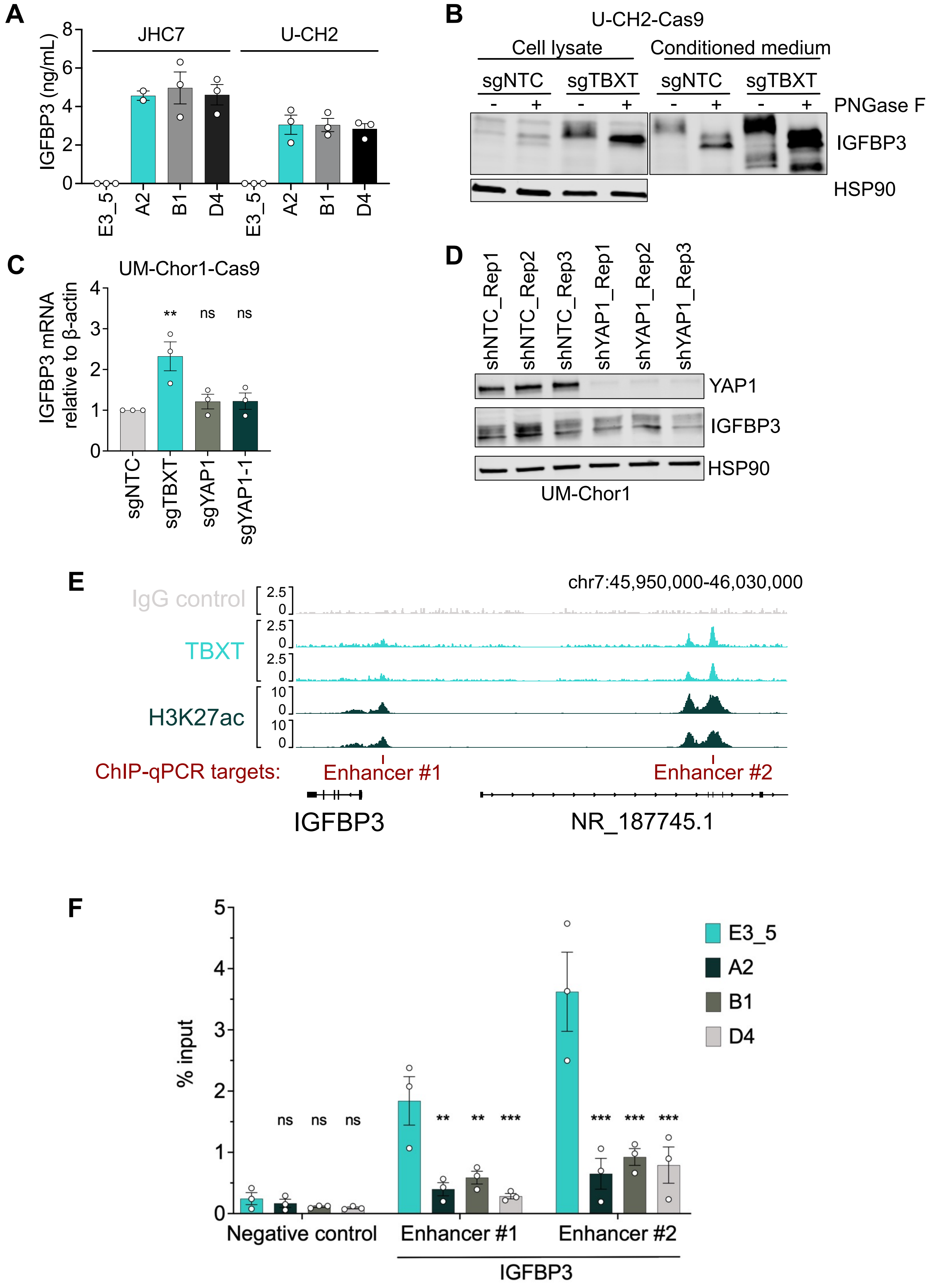

Supplementary Figure 5 continued

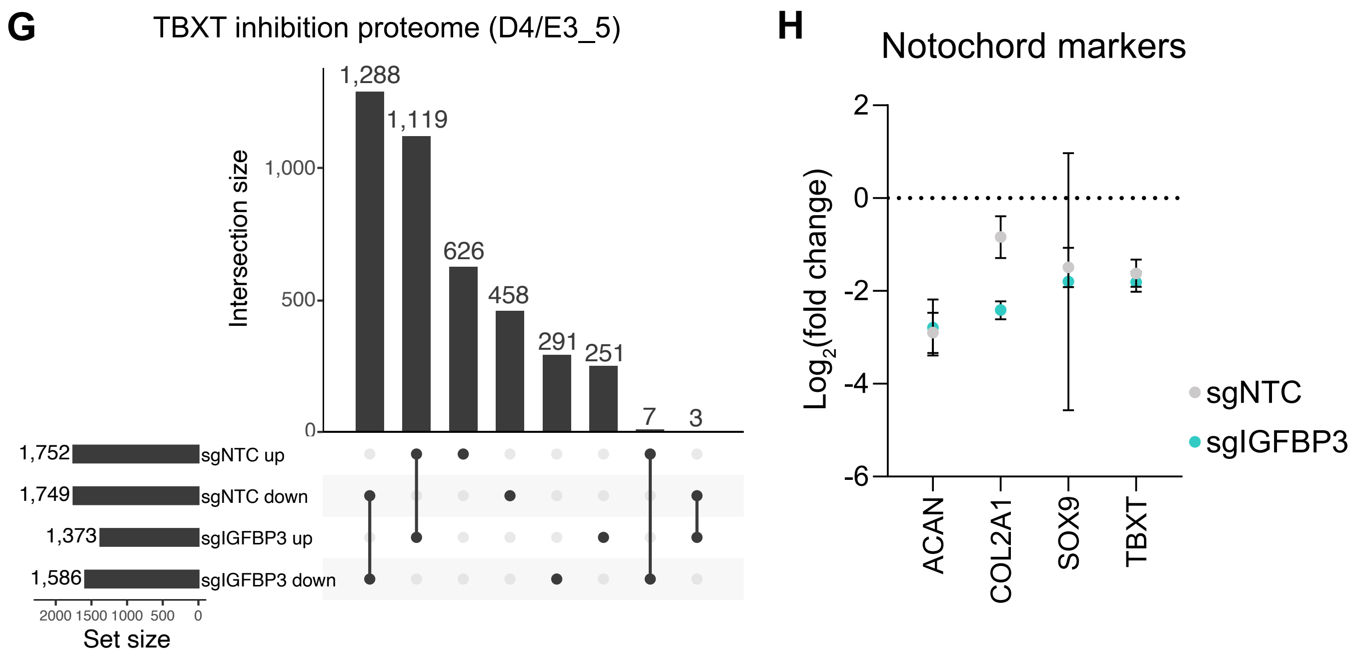

Fig. S5. Relationship between IGFBP3 expression and TBXT inhibition. (**A**) Cellular protein levels of IGFBP3 quantified by ELISA in JHC7 and U-CH2 cells transduced with DARPins E3_5, A2, B1, or D4. Mean + SEM with two (JHC7 A2) or three (all other conditions) biological replicates. (**B**) Western blot with cell lysates and culture medium of U-CH2-Cas9 cells transduced with sgNTC or sgTBXT. Samples were treated with or without the glycosidase PNGase F. (**C**) *IGFBP3* mRNA levels measured by quantitative RT-PCR in UM-Chor1-Cas9 cells transduced with sgNTC, sgTBXT, or two different sgRNAs targeting YAP1. One-way ANOVA with Dunnett's test for multiple comparisons; mean + SEM of three biological replicates. $^{**}p \leq 0.01$, ns, not significant. (**D**) Western blot of UM-Chor1 cells transduced with an shRNA targeting YAP1 or an shNTC in biological triplicate (Rep1-3). (**E**) Genome browser snapshots of publicly available ChIPmentation data (GSE153971) in UM-Chor1 cells. Shown are tracks from three pulldown conditions: IgG control (anti-HA in parental UM-Chor1 cells; gray), TBXT ChIP (two replicates, anti-HA in HA-dTAG-TBXT, TBXT−/− UM-Chor1 cells; light blue), and H3K27ac ChIP (two replicates, anti-H3K27ac in HA-dTAG-TBXT, TBXT−/− UM-Chor1 cells; black). Two putative *cis*-regulatory elements upstream of the *IGFBP3* promoter, highlighted in red, were selected for further validation. (**F**) ChIP-qPCR analysis of TBXT occupancy at the two putative regulatory elements in UM-Chor1 cells expressing either control DARPin E3_5 or the T-DARPins A2, B1, and D4. Enrichment was measured at a negative control region (*KLK3*) and the two candidate enhancer regions shown in (E) by qPCR. Two-way ANOVA with Dunnett's test for multiple comparisons vs. negative control E3_5; mean ± SEM of three biological replicates. ns, not significant, $^{**}p \leq 0.01$, $^{***}p \leq 0.001$. (**G**) UpSet plot showing set and intersection sizes for proteins that are up- or downregulated upon TBXT inhibition with (sgIGFBP3) and without (sgNTC) IGFBP3 knock-out ($\log_2$(fold change) > 0.2 or < -0.02, FDR < 5% by GLM with a Wald test and Benjamini-Hochberg correction. (**H**) Effect of TBXT inhibition (D4/E3_5) on UM-Chor1-Cas9 cells stably expressing sgNTC or sgIGFBP3. Protein levels from DIA-MS graphed as $\log_2$(fold change) with upper and lower confidence intervals for four notochord marker genes. Log_2(fold change) was calculated as in G.

Supplementary Figure 6

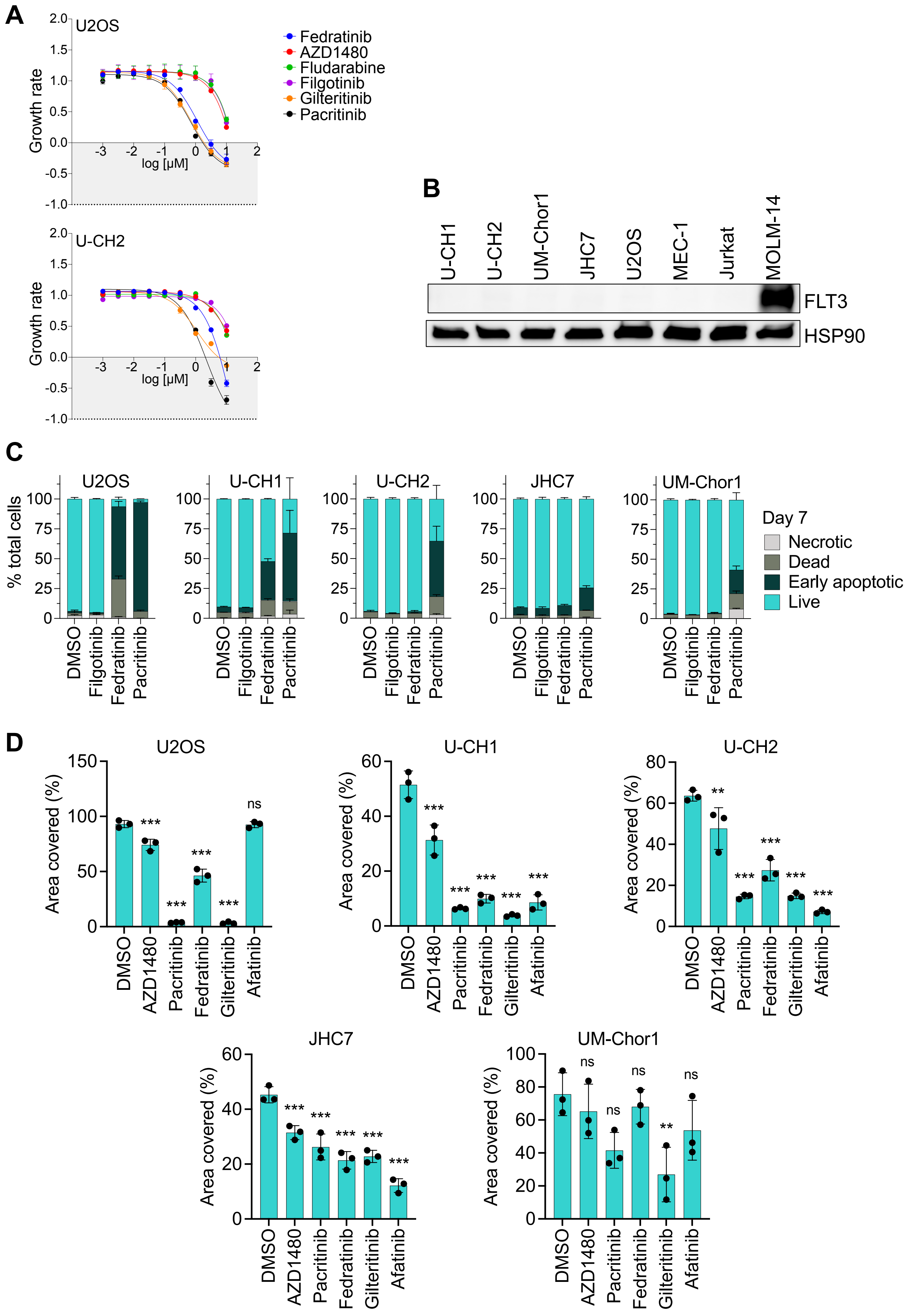

Fig. S6. Drug response of chordoma cell lines. (**A**) GR analysis of the indicated cell lines after seven days of treatment with the pan-JAK inhibitor filgotinib, the JAK2 inhibitors AZD1480, fedratinib, and pacritinib, the STAT1 inhibitor fludarabine, and the FLT3 inhibitor gilteritinib at concentrations of 10 μM to 1 nM. GR values between 1 and 0 indicate proliferation inhibition, 0 indicates a complete cytostatic effect, and values between 0 and −1 indicate additional cytotoxicity. One of three representative experiments is shown, with each data point representing a technical triplicate. (**B**) Western blot for FLT3 in chordoma cells and the FLT3-positive leukemia cell line MOLM-14. (**C**) Apoptosis measured by flow cytometry after staining with annexin V and 7-AAD of cell lines treated with 3 μM of the indicated drugs for seven days. Mean + SEM of two biological experiments. (**D**) Quantification of colony formation assay shown in Fig. 6F with U2OS and the indicated chordoma cell lines treated for 14 days with 1 μM of the indicated drugs. Mean ± SEM of three independent experiments. One-way ANOVA with Dunnett's test for multiple comparisons; *$p \leq 0.05$, **$p \leq 0.01$, ***$p \leq 0.001$, ns, not significant.

Table S1: Primers used for site-directed mutagenesis

Name	Primer sequence (5‘ to 3‘)
R16L forward	CTCAGCAGATGATCAACAAGATACTGCAGGCTTTTAC
R16L reverse	GTAAAAGCCTGCAGTATCTTGTTGATCATCTGCTGAG
H171R forward	GACCACCAACACGAACAATACGAATACGCGGTTCATATTTA
H171R reverse	TAAATATGAACCGCGTATTCGTATTGTTCGTGTTGGTGGTC
G177D forward	CATATTGTTCGTGTTGGTGATCCGCAGCGTATGATTACC
G177D reverse	GGTAATCATACGCTGCGGATCACCAACACGAACAATATG
ΔDBD forward	GTGATCCGACCGAACGTGAAGCAAAAGAACGTAGC
ΔDBD reverse	GCTACGTTCTTTTGCTTCACGTTCGGTCGGATCAC

Table S2: sgRNA oligonucleotides

Name	sgRNA sequence (5‘ to 3‘)
sgNTC	AAAAAGCTTCCGCCTGATGG
sgTBXT	TGGCTGGTGATCATGCGCTG
sgYAP1	GTGCACGATCTGATGCCCGG
sgYAP1-1	TGCCCCAGACCGTGCCCATG
sgIGFBP3	CACCAGCTCCGCGCACACGG

Table S3: miR-E shRNA oligonucleotides (sense in bold)

Name	shRNA sequence (5‘ to 3‘)
shLuc	TGCTGTTGACAGTGAGCGC**AGGAATTATAATGCTTATCT**ATAGTGAAGCCACAGATGTATAGATAAGCATTATAATTCCTATGCCTACTGCCTCGGA
shTBXT	TGCTGTTGACAGTGAGCGC**AAGTACAATCCATTTGCAAA**ATAGTGAAGCCACAGATGTATTTTGCAAATGGATTGTACTTATGCCTACTGCCTCGGA

Table S4: shRNA oligonucleotides (sense)

Name	shRNA sequence (5‘ to 3‘)
shNTC	CAACAAGATGAAGAGCACCAA
shYAP	CCCAGTTAAATGTTCACCAAT

Table S5: Primer sequences used for ChIP-qPCR.

Target gene	Genomic coordinates (hg19)	Primer	Primer sequence (5' to 3')
KLK3	chr19: 51361368-51361474	Forward<br>Reverse	CACACCCGCTCTACGATATGA<br>GAGCTCGGCAGGCTCTGA
KRT8	chr12: 53312519-53312591	Forward<br>Reverse	GCTTGACTCTCACCAACCCTC<br>TGGGGGAAATTGTGTGGAGA
TBXT	chr6: 166607729-166607832	Forward<br>Reverse	TCCACATCTGCAAACCAGCC<br>TCAAGGAGTGCTGAGTCCGA
COL5A2	chr2: 190057585-190057722	Forward<br>Reverse	TCTTTATCTCAAGCATGGGTTCCT<br>CCAAATGCGTCCTCTTGTGA
C7orf69	chr7: 47811747-47811857	Forward<br>Reverse	GCTAGGACCTGGATGTGTGG<br>CCATAGGCGTGAGAGGAGTTC
SOX9	chr17: 70093678-70093815	Forward<br>Reverse	ACCACCAGAGGGTGTTCTGT<br>TCCAGCTCAACTCTTTCCACA
IGFBP3 Enhancer 1	chr7: 45964140-45964217	Forward<br>Reverse	ACAGCAGAGAAAACAGAGAGGT<br>TGACCACACGGACAGGTTTG
IGFBP3 Enhancer 2	chr7: 46017893-46017964	Forward<br>Reverse	CAGCCCTCCTGCCACTTTAG<br>TCAGCATTCTCTGTCTCTGGAA

Table S6: Primary and secondary antibodies used for western blotting

Name	Source species	Dilution	Company	Article #
Anti-TBXT (D2Z3J)	Rabbit	1:1,000 in 5% BSA/TBST	Cell Signaling	81694
Anti-FLAG	Rabbit	1:400 in 5% BSA/TBST	Sigma-Aldrich	F7425
Anti-β-actin (AC-15)	Mouse	1:2,000 in 5% BSA/TBST	Sigma-Aldrich	A1978
Anti-β-actin	Rabbit	1:1,000 in 5% BSA/TBST	Cell Signaling	4967

Anti-HSP90 (F-8)	Mouse	1:5,000 in 5% BSA/TBST	Santa Cruz	sc-13119
Anti-YAP (D8H1X)	Rabbit	1:1,000 in 5% BSA/TBST	Cell Signaling	14074
Anti-IGFBP3 (EPR18680-153)	Rabbit	1:1,000 in 5% BSA /TBST	Abcam	ab193910
Anti-IGFBP3 (D1U9C)	Rabbit	1:1,000 in 5% BSA/TBST	Cell Signaling	25864
Anti-ISG15 (EPR3446)	Rabbit	1:1,000 in 5% BSA/TBST	Abcam	ab133346
Anti-JAK1	Rabbit	1:1,000 in 5% BSA/TBST	Cell Signaling	3332
Anti-JAK2 (D2E12)	Rabbit	1:1,000 in 5% BSA/TBST	Cell Signaling	3230
Anti-JAK3	Rabbit	1:1,000 in 5% BSA/TBST	Cell Signaling	3775
Anti-TYK2 (D4I5T)	Rabbit	1:1,000 in 5% BSA/TBST	Cell Signaling	14193
Anti-STAT1 (D1K9Y)	Rabbit	1:1,000 in 5% BSA/TBST	Cell Signaling	14994
Anti-STAT2 (D9J7L)	Rabbit	1:1,000 in 5% BSA/TBST	Cell Signaling	72604
Anti-STAT3 (124H6)	Mouse	1:1,000 in 5% BSA/TBST	Cell Signaling	9139
Anti-STAT4 (C46B10)	Rabbit	1:1,000 in 5% BSA/TBST	Cell Signaling	2653
Anti-STAT5 (D206Y)	Rabbit	1:1,000 in 5% BSA/TBST	Cell Signaling	94205
Anti-STAT6 (D3H4)	Rabbit	1:1,000 in 5% BSA/TBST	Cell Signaling	5397
Anti-IRF9 (D2T8M)	Rabbit	1:1,000 in 5% BSA/TBST	Cell Signaling	76684
Anti-EGFR	Rabbit	1:1,000 in 5% BSA/TBST	Cell Signaling	4267
Anti-pEGFR(Y1068)	Rabbit	1:1,000 in 5% BSA/TBST	Cell Signaling	3777
Anti-mouse IgG (H+L) DyLight 680 Conjugate	Goat	1:15,000 in 5% milk/TBST	Cell Signaling	5470
Anti-rabbit IgG (H+L) DyLight 800 4X PEG Conjugate	Goat	1:15,000 in 5% milk/TBST	Cell Signaling	5151

Anti-rabbit IgG, HRP-linked	Goat	1:100,000 in 5% milk/TBST	Cell Signaling	7074
Anti-mouse IgG, HRP-linked	Horse	1:100,000 in 5% milk/TBST	Cell Signaling	7076

Table S7: Primer sequences used for quantitative RT-PCR.

Name	Primer sequence (5‘ to 3‘)
SOX9 forward	AGCGAACGCACATCAAGAC
SOX9 reverse	CTGTAGGCGATCTGTTGGGG
TBXT forward	TATGAGCCTCGAATCCACATAGT
TBXT reverse	CCTCGTTCTGATAAGCAGTCAC
ACTB forward	CATGTACGTTGCTATCCAGGC
ACTB reverse	CTCCTTAATGTCACGCACGAT

Other Supplementary Materials for this manuscript include the following:

Data S1: Affinity purification mass spectrometry data

Data S2: Significantly differentially expressed genes in UM-Chor1 cells after TBXT inhibition with three T-DARPins

Data S3: Gene set enrichment analysis of RNA-seq data

Data S4: Proteins detected in UM-Chor1 cells after TBXT inhibition with three DARPins and TBXT knockout

Data S5: Gene set enrichment analysis of the DDA-MS proteome data

Data S6: Categorization of the TBXT-regulated DEGs and DEPs according to their target development levels

Data S7: DIA-MS proteomics of IGFBP3 wild-type and knock-out cells in combination with expression of DARPins D4 or E3_5

REFERENCES AND NOTES

1. P. Das, P. Soni, J. Jones, G. Habboub, J. S. Barnholtz-Sloan, P. F. Recinos, V. R. Kshettry, Descriptive epidemiology of chordomas in the United States. *J. Neurooncol* **148**, 173–178 (2020).

2. S. P. Kelley, R. U. Ashford, A. S. Rao, R. A. Dickson, Primary bone tumours of the spine: A 42-year survey from the Leeds regional bone tumour registry. *Eur. Spine J.* **16**, 405–409 (2007).

3. V. Colia, S. Stacchiotti, Medical treatment of advanced chordomas. *Eur. J. Cancer* **83**, 220–228 (2017).

4. R. Golding, R. Abuqubo, C. J. Pansa, M. Bhatta, V. Shankar, K. Mani, E. Kleinbart, Y. Gelfand, S. Murthy, R. De la Garza Ramos, J. Krystal, A. Eleswarapu, R. Yassari, E. Mostafa, M. S. Fourman, A. Schlumprecht, Immunologic and targeted molecular therapies for chordomas: A narrative review. *J. Clin. Med.* **13**, 5679 (2024).

5. W. Hsu, A. Mohyeldin, S. R. Shah, C. M. ap Rhys, L. F. Johnson, N. I. Sedora-Roman, T. A. Kosztowski, O. A. Awad, E. F. McCarthy, D. M. Loeb, J.-P. Wolinsky, Z. L. Gokaslan, A. Quiñones-Hinojosa, Generation of chordoma cell line JHC7 and the identification of Brachyury as a novel molecular target. *J. Neurosurg.* **115**, 760–769 (2011).

6. P. S. Tarpey, S. Behjati, M. D. Young, I. Martincorena, L. B. Alexandrov, S. J. Farndon, C. Guzzo, C. Hardy, C. Latimer, A. P. Butler, J. W. Teague, A. Shlien, P. A. Futreal, S. Shah, A. Bashashati, F. Jamshidi, T. O. Nielsen, D. Huntsman, D. Baumhoer, S. Brandner, J. Wunder, B. Dickson, P. Cogswell, J. Sommer, J. J. Phillips, M. F. Amary, R. Tirabosco, N. Pillay, S. Yip, M. R. Stratton, A. M. Flanagan, P. J. Campbell, The driver landscape of sporadic chordoma. *Nat. Commun.* **8**, 1–6 (2017).

7. T. Sharifnia, M. J. Wawer, T. Chen, Q.-Y. Huang, B. A. Weir, A. Sizemore, M. A. Lawlor, A. Goodale, G. S. Cowley, F. Vazquez, C. J. Ott, J. M. Francis, S. Sassi, P. Cogswell, H. E. Sheppard, T. Zhang, N. S. Gray, P. A. Clarke, J. Blagg, P. Workman, J. Sommer, F. Hornicek, D. E. Root, W. C. Hahn, J. E. Bradner, K. K. Wong, P. A. Clemons, C. Y. Lin, J. D. Kotz, S. L.

Schreiber, Small-molecule targeting of brachyury transcription factor addiction in chordoma. *Nat. Med.* **9**, 292–300 (2019).

8. H. E. Sheppard, A. Dall'Agnese, W. D. Park, M. H. Shamim, J. Dubrulle, H. L. Johnson, F. Stossi, P. Cogswell, J. Sommer, J. Levy, T. Sharifnia, M. J. Wawer, B. Nabet, N. S. Gray, P. A. Clemons, S. L. Schreiber, P. Workman, R. A. Young, C. Y. Lin, Targeted brachyury degradation disrupts a highly specific autoregulatory program controlling chordoma cell identity. *Cell Rep. Med.* **2**, 100188 (2021).

9. A. Kispert, B. Koschorz, B. G. Herrmann, The T protein encoded by brachyury is a tissue-specific transcription factor. *EMBO J.* **14**, 4763–4772 (1995).

10. C. Papapetrou, Y. H. Edwards, J. C. Sowden, The T transcription factor functions as a dimer and exhibits a common human polymorphism Gly-177-Asp in the conserved DNA-binding domain. *FEBS Lett.* **409**, 201–206 (1997).

11. C. W. Müller, B. G. Herrmann, Crystallographic structure of the T domain-DNA complex of the brachyury transcription factor. *Nature* **389**, 884–888 (1997).

12. A. A. Shihabi, A. Davarifar, H. T. L. Nguyen, N. Tavanaie, S. D. Nelson, J. Yanagawa, N. Federman, N. Bernthal, F. Hornicek, A. Soragni, Personalized chordoma organoids for drug discovery studies. *Sci. Adv.* **8**, eabl3674 (2022).

13. S. Stacchiotti, A. Longhi, V. Ferraresi, G. Grignani, A. Comandone, R. Stupp, A. Bertuzzi, E. Tamborini, S. Pilotti, A. Messina, C. Spreafico, A. Gronchi, P. Amore, V. Vinaccia, P. G. Casali, Phase II study of imatinib in advanced chordoma. *J. Clin. Oncol.* **30**, 914–920 (2012).

14. P. Magnaghi, B. Salom, L. Cozzi, N. Amboldi, D. Ballinari, E. Tamborini, F. Gasparri, A. Montagnoli, L. Raddrizzani, A. Somaschini, R. Bosotti, C. Orrenius, F. Bozzi, S. Pilotti, A. Galvani, J. Sommer, S. Stacchiotti, A. Isacchi, Afatinib is a new therapeutic approach in chordoma with a unique ability to target EGFR and brachyury. *Mol. Cancer Ther.* **17**, 603–613 (2018).

15. K. T. G. Samarasinghe, S. Jaime-Figueroa, M. Burgess, D. A. Nalawansha, K. Dai, Z. Hu, A. Bebenek, S. A. Holley, C. M. Crews, Targeted degradation of transcription factors by TRAFTACs: TRAnscription Factor TArgeting Chimeras. *Cell Chem. Biol.* **28**, 648–661.e5 (2021).

16. D. H. Chase, A. M. Bebenek, P. Nie, S. Jaime-Figueroa, A. Butrin, D. A. Castro, J. Hines, B. M. Linhares, C. M. Crews, Development of a small molecule downmodulator for the transcription factor brachyury. *Angew. Chem. Int. Ed. Engl.* **63**, e202316496 (2024).

17. J. A. Newman, A. E. Gavard, N. Imprachim, H. Aitkenhead, H. E. Sheppard, R. te Poele, P. A. Clarke, M. A. Hossain, L. Temme, H. J. Oh, C. I. Wells, Z. W. Davis-Gilbert, P. Workman, O. Gileadi, D. H. Drewry, Structural insights into human brachyury DNA recognition and discovery of progressible binders for cancer therapy. *Nat. Commun.* **16**, 1596 (2025).

18. A. Plückthun, Designed ankyrin repeat proteins (DARPins): Binding proteins for research, diagnostics, and therapy. *Annu. Rev. Pharmacol. Toxicol.* **55**, 489–511 (2015).

19. D. Callanan, D. Kunimoto, R. K. Maturi, S. S. Patel, G. Staurenghi, S. Wolf, J. K. Cheetham, T. C. Hohman, K. Kim, F. J. López, S. Schneider, J. Arnold, R. Avery, M. Bennett, B. Berger, B. Blodi, D. Boyer, D. Brown, D. Callanan, K. Csaky, F. Devin, D. W. Faber, S. Fraser-Bell, J. R. Ferencz, C. Framme, D. Gaucher, M. Gillies, M. Goldstein, R. Guymer, J. Heier, F. Holz, R. N. Khurana, G. Kokame, J.-F. Korobelnik, D. Kunimoto, P. Lanzetta, C. P. Lohmann, R. Maturi, S. Mohand-Said, G. Papastergiou, S. Patel, D. Pauleikhoff, A. Pollack, C. Pruente, S. Rose, I. Rosenblatt, U. Schmidt-Erfurth, A. Sharma, L. Singerman, E. Souied, G. Staurenghi, P. Tornambe, C. Wykoff, M. Zinkernagel, Double-masked, randomized, phase 2 evaluation of abicipar pegol (an anti-VEGF DARPin therapeutic) in neovascular age-related macular degeneration. *J. Ocul. Pharmacol. Ther.* **34**, 700–709 (2018).

20. M. Schmid, P. Ernst, A. Honegger, M. Suomalainen, M. Zimmermann, L. Braun, S. Stauffer, C. Thom, B. Dreier, M. Eibauer, A. Kipar, V. Vogel, U. F. Greber, O. Medalia, A. Plückthun, Adenoviral vector with shield and adapter increases tumor specificity and escapes liver and immune control. *Nat. Commun.* **9**, 450 (2018).

21. N. Bery, S. Legg, J. Debreczeni, J. Breed, K. Embrey, C. Stubbs, P. Kolasinska-Zwierz, N. Barrett, R. Marwood, J. Watson, J. Tart, R. Overman, A. Miller, C. Phillips, R. Minter, T. H. Rabbitts, KRAS-specific inhibition using a DARPin binding to a site in the allosteric lobe. *Nat. Commun.* **10**, 2607 (2019).

22. H. K. Binz, M. T. Stumpp, P. Forrer, P. Amstutz, A. Plückthun, Designing repeat proteins: Well-expressed, soluble and stable proteins from combinatorial libraries of consensus ankyrin repeat proteins. *J. Mol. Biol.* **332**, 489–503 (2003).

23. S. R. Shah, J. M. David, N. D. Tippens, A. Mohyeldin, J. C. Martinez-Gutierrez, S. Ganaha, P. Schiapparelli, D. H. Hamilton, C. Palena, A. Levchenko, A. Quiñones-Hinojosa, Brachyury-YAP regulatory axis drives stemness and growth in cancer. *Cell Rep.* **21**, 495–507 (2017).

24. V. E. Papaioannou, The T-box gene family: Emerging roles in development, stem cells and cancer. *Development* **141**, 3819–3833 (2014).

25. R. V. Honorato, M. E. Trellet, B. Jiménez-García, J. J. Schaarschmidt, M. Giulini, V. Reys, P. I. Koukos, J. P. G. L. M. Rodrigues, E. Karaca, G. C. P. van Zundert, J. Roel-Touris, C. W. van Noort, Z. Jandová, A. S. J. Melquiond, A. M. J. J. Bonvin, The HADDOCK2.4 web server for integrative modeling of biomolecular complexes. *Nat. Protoc.* **19**, 3219–3241 (2024).

26. C. Bettegowda, S. Yip, S.-F. L. Lo, C. G. Fisher, S. Boriani, L. D. Rhines, J. Y. Wang, A. Lazary, M. Gambarotti, W.-L. Wang, A. Luzzati, M. B. Dekutoski, M. H. Bilsky, D. Chou, M. G. Fehlings, E. F. McCarthy, N. A. Quraishi, J. J. Reynolds, D. M. Sciubba, R. P. Williams, J.-P. Wolinsky, P. L. Zadnik, M. Zhang, N. M. Germscheid, V. Kalampoki, P. P. Varga, Z. L. Gokaslan, Aos. K. F. Tumor, spinal column chordoma: Prognostic significance of clinical variables and T (brachyury) gene SNP rs2305089 for local recurrence and overall survival. *Neuro Oncol.* **19**, 405–413 (2017).

27. X. Feng, J. P. Y. Cheung, J. S. H. Je, P. W. H. Cheung, S. Chen, M. Yue, N. Wang, V. N. T. Choi, X. Yang, Y. Song, K. D. K. Luk, B. Gao, Genetic variants of TBX6 and TBXT identified in patients with congenital scoliosis in Southern China. *J. Orthop. Res.* **39**, 971–988 (2021).

28. A. V. Postma, M. Alders, M. Sylva, C. M. Bilardo, E. Pajkrt, R. R. van Rijn, S. Schulte-Merker, S. Bulk, S. Stefanovic, A. Ilgun, P. Barnett, M. M. A. M. Mannens, A. F. M. Moorman, R. J. Oostra, M. C. van Maarle, Mutations in the T (brachyury) gene cause a novel syndrome consisting of sacral agenesis, abnormal ossification of the vertebral bodies and a persistent notochordal canal. *J. Med. Genet.* **51**, 90–97 (2014).

29. A. E. Wehmeyer, K. M. Schüle, A. Conrad, C. M. Schröder, S. Probst, S. J. Arnold, Chimeric 3D-gastruloids—A versatile tool for studies of mammalian peri-gastrulation development. *Development* **149**, dev200812 (2022).

30. S. C. Halvorsen, Y. Benita, M. Hopton, B. Hoppe, H. O. Gunnlaugsson, P. Korgaonkar, C. R. Vanderburg, G. P. Nielsen, N. Trepanowski, J. H. Cheah, M. P. Frosch, J. H. Schwab, A. E. Rosenberg, F. J. Hornicek, S. Sassi, Transcriptional profiling supports the notochordal origin of chordoma and its dependence on a TGFB1-TBXT network. *Am. J. Pathol.* **193**, 532–547 (2023).

31. S. H. Peck, K. K. McKee, J. W. Tobias, N. R. Malhotra, B. D. Harfe, L. J. Smith, Whole transcriptome analysis of notochord-derived cells during embryonic formation of the nucleus pulposus. *Sci. Rep.* **7**, 10504 (2017).

32. M. Park, I. Park, C.-K. Hong, S. H. Kim, Y. J. Cha, Differences in stromal component of chordoma are associated with contrast enhancement in MRI and differential gene expression in RNA sequencing. *Sci. Rep.* **12**, 16504 (2022).

33. J. Jiang, J. Yuan, Z. Hu, Y. Zhang, T. Zhang, M. Xu, M. Long, Y. Fan, J. L. Tanyi, K. T. Montone, O. Tavana, R. H. Vonderheide, H. M. Chan, X. Hu, L. Zhang, Systematic illumination of druggable genes in cancer genomes. *Cell Rep.* **38**, 110400 (2022).

34. S. Gröschel, D. Hübschmann, F. Raimondi, P. Horak, G. Warsow, M. Fröhlich, B. Klink, L. Gieldon, B. Hutter, K. Kleinheinz, D. Bonekamp, O. Marschal, P. Chudasama, J. Mika, M. Groth, S. Uhrig, S. Krämer, C. Heining, C. E. Heilig, D. Richter, E. Reisinger, K. Pfütze, R. Eils, S. Wolf, C. Kalle, C. Brandts, C. Scholl, W. Weichert, S. Richter, S. Bauer, R. Penzel, E. Schröck, A. Stenzinger, R. F. Schlenk, B. Brors, R. B. Russell, H. Glimm, M. Schlesner, S.

Fröhling, Defective homologous recombination DNA repair as therapeutic target in advanced chordoma. *Nat. Commun.* **10**, 1–9 (2019).

35. B. Lohberger, S. Scheipl, E. Heitzer, F. Quehenberger, D. de Jong, K. Szuhai, B. Liegl-Atzwanger, B. Rinner, Higher cMET dependence of sacral compared to clival chordoma cells: Contributing to a better understanding of cMET in chordoma. *Sci. Rep.* **11**, 12466 (2021).

36. J. Wang, G. J. S. Lohman, J. Stubbe, Enhanced subunit interactions with gemcitabine-5′-diphosphate inhibit ribonucleotide reductases. *Proc. Natl. Acad. Sci. U.S.A.* **104**, 14324–14329 (2007).

37. K. J. Mavrakis, E. R. McDonald III, M. R. Schlabach, E. Billy, G. R. Hoffman, A. deWeck, D. A. Ruddy, K. Venkatesan, J. Yu, G. McAllister, M. Stump, R. deBeaumont, S. Ho, Y. Yue, Y. Liu, Y. Yan-Neale, G. Yang, F. Lin, H. Yin, H. Gao, D. R. Kipp, S. Zhao, J. T. McNamara, E. R. Sprague, B. Zheng, Y. Lin, Y. S. Cho, J. Gu, K. Crawford, D. Ciccone, A. C. Vitari, A. Lai, V. Capka, K. Hurov, J. A. Porter, J. Tallarico, C. Mickanin, E. Lees, R. Pagliarini, N. Keen, T. Schmelzle, F. Hofmann, F. Stegmeier, W. R. Sellers, Disordered methionine metabolism in MTAP/CDKN2A-deleted cancers leads to dependence on PRMT5. *Science* **351**, 1208–1213 (2016).

38. G. V. Kryukov, F. H. Wilson, J. R. Ruth, J. Paulk, A. Tsherniak, S. E. Marlow, F. Vazquez, B. A. Weir, M. E. Fitzgerald, M. Tanaka, C. M. Bielski, J. M. Scott, C. Dennis, G. S. Cowley, J. S. Boehm, D. E. Root, T. R. Golub, C. B. Clish, J. E. Bradner, W. C. Hahn, L. A. Garraway, MTAP deletion confers enhanced dependency on the PRMT5 arginine methyltransferase in cancer cells. *Science* **351**, 1214–1218 (2016).

39. O. C. Olson, J. A. Joyce, Cysteine cathepsin proteases: Regulators of cancer progression and therapeutic response. *Nat. Rev. Cancer* **15**, 712–729 (2015).

40. S. V. Shrivastav, A. Bhardwaj, K. A. Pathak, A. Shrivastav, Insulin-like growth factor binding protein-3 (IGFBP-3): Unraveling the role in mediating IGF-independent effects within the cell. *Front. Cell Dev. Biol.* **8**, 286 (2020).

41. V. Gorgoulis, P. D. Adams, A. Alimonti, D. C. Bennett, O. Bischof, C. Bishop, J. Campisi, M. Collado, K. Evangelou, G. Ferbeyre, J. Gil, E. Hara, V. Krizhanovsky, D. Jurk, A. B. Maier, M. Narita, L. Niedernhofer, J. F. Passos, P. D. Robbins, C. A. Schmitt, J. Sedivy, K. Vougas, T. von Zglinicki, D. Zhou, M. Serrano, M. Demaria, Cellular senescence: Defining a path forward. *Cell* **179**, 813–827 (2019).

42. J. M. Franklin, R. P. Ghosh, Q. Shi, M. P. Reddick, J. T. Liphardt, Concerted localization-resets precede YAP-dependent transcription. *Nat. Commun.* **11**, 4581 (2020).

43. P. Horak, C. Heining, S. Kreutzfeldt, B. Hutter, A. Mock, J. Hullein, M. Frohlich, S. Uhrig, A. Jahn, A. Rump, L. Gieldon, L. Mohrmann, D. Hanf, V. Teleanu, C. E. Heilig, D. B. Lipka, M. Allgauer, L. Ruhnke, A. Lassmann, V. Endris, O. Neumann, R. Penzel, K. Beck, D. Richter, U. Winter, S. Wolf, K. Pfutze, C. Georg, B. Meissburger, I. Buchhalter, M. Augustin, W. E. Aulitzky, P. Hohenberger, M. Kroiss, P. Schirmacher, R. F. Schlenk, U. Keilholz, F. Klauschen, G. Folprecht, S. Bauer, J. T. Siveke, C. H. Brandts, T. Kindler, M. Boerries, A. L. Illert, N. von Bubnoff, P. J. Jost, K. Spiekermann, M. Bitzer, K. Schulze-Osthoff, C. von Kalle, B. Klink, B. Brors, A. Stenzinger, E. Schrock, D. Hubschmann, W. Weichert, H. Glimm, S. Frohling, Comprehensive genomic and transcriptomic analysis for guiding therapeutic decisions in patients with rare cancers. *Cancer Discov.* **11**, 2780–2795 (2021).

44. C. E. Heilig, A. Laßmann, S. S. Mughal, A. Mock, S. Pirmann, V. Teleanu, M. Renner, C. Andresen, B. C. Köhler, B. Aybey, S. Bauer, J. T. Siveke, R. Hamacher, G. Folprecht, S. Richter, E. Schröck, C. H. Brandts, M. Ahrens, P. Hohenberger, G. Egerer, T. Kindler, M. Boerries, A. L. Illert, N. von Bubnoff, L. Apostolidis, P. J. Jost, C. B. Westphalen, W. Weichert, U. Keilholz, F. Klauschen, K. Beck, U. Winter, D. Richter, L. Möhrmann, M. Bitzer, K. Schulze-Osthoff, B. Brors, G. Mechtersheimer, S. Kreutzfeldt, C. Heining, D. B. Lipka, A. Stenzinger, R. F. Schlenk, P. Horak, H. Glimm, D. Hübschmann, S. Fröhling, Gene expression-based prediction of pazopanib efficacy in sarcoma. *Eur. J. Cancer* **172**, 107–118 (2022).

45. S. M. Corsello, R. T. Nagari, R. D. Spangler, J. Rossen, M. Kocak, J. G. Bryan, R. Humeidi, D. Peck, X. Wu, A. A. Tang, V. M. Wang, S. A. Bender, E. Lemire, R. Narayan, P. Montgomery, U. Ben-David, C. W. Garvie, Y. Chen, M. G. Rees, N. J. Lyons, J. M. McFarland, B. T. Wong,

L. Wang, N. Dumont, P. J. O'Hearn, E. Stefan, J. G. Doench, C. N. Harrington, H. Greulich, M. Meyerson, F. Vazquez, A. Subramanian, J. A. Roth, J. A. Bittker, J. S. Boehm, C. C. Mader, A. Tsherniak, T. R. Golub, Discovering the anticancer potential of non-oncology drugs by systematic viability profiling. *Nat. Cancer* **1**, 235–248 (2020).

46. X. Hu, J. Li, M. Fu, X. Zhao, W. Wang, The JAK/STAT signaling pathway: From bench to clinic. *Signal Transduct. Target. Ther.* **6**, 402 (2021).

47. D. A. Frank, S. Mahajan, J. Ritz, Fludarabine-induced immunosuppression is associated with inhibition of STAT1 signaling. *Nat. Med.* **5**, 444–447 (1999).

48. L. Y. Lee, D. Hernandez, T. Rajkhowa, S. C. Smith, J. R. Raman, B. Nguyen, D. Small, M. Levis, Preclinical studies of gilteritinib, a next-generation FLT3 inhibitor. *Blood* **129**, 257–260 (2017).

49. M. Hafner, M. Niepel, M. Chung, P. K. Sorger, Growth rate inhibition metrics correct for confounders in measuring sensitivity to cancer drugs. *Nat. Methods* **13**, 521–527 (2016).

50. Y. L. Boersma, G. Chao, D. Steiner, K. D. Wittrup, A. Plückthun, Bispecific designed ankyrin repeat proteins (DARPins) targeting epidermal growth factor receptor inhibit A431 cell proliferation and receptor recycling. *J. Biol. Chem.* **286**, 41273–41285 (2011).

51. S. Rothenberger, D. L. Hurdiss, M. Walser, F. Malvezzi, J. Mayor, S. Ryter, H. Moreno, N. Liechti, A. Bosshart, C. Iss, V. Calabro, A. Cornelius, T. Hospodarsch, A. Neculcea, T. Looser, A. Schlegel, S. Fontaine, D. Villemagne, M. Paladino, D. Schiegg, S. Mangold, C. Reichen, F. Radom, Y. Kaufmann, D. Schaible, I. Schlegel, C. Zitt, G. Sigrist, M. Straumann, J. Wolter, M. Comby, F. Sacarcelik, I. Drulyte, H. Lyoo, C. Wang, W. Li, W. Du, H. K. Binz, R. Herrup, S. Lusvarghi, S. N. Neerukonda, R. Vassell, W. Wang, J. M. Adler, K. Eschke, M. Nascimento, A. Abdelgawad, A. D. Gruber, J. Bushe, O. Kershaw, C. G. Knutson, K. K. Balavenkatraman, K. Ramanathan, E. Wyler, L. G. T. Alves, S. Lewis, R. Watson, M. A. Haeuptle, A. Zürcher, K. M. Dawson, D. Steiner, C. D. Weiss, P. Amstutz, F. J. M. van Kuppeveld, M. T. Stumpp, B.-J. Bosch, O. Engler, J. Trimpert, The trispecific DARPin ensovibep inhibits diverse SARS-CoV-2 variants. *Nat. Biotechnol.* **40**, 1845–1854 (2022).

52. K. Deprey, L. Becker, J. Kritzer, A. Plückthun, Trapped! A critical evaluation of methods for measuring total cellular uptake versus cytosolic localization. *Bioconjug. Chem.* **30**, 1006–1027 (2019).

53. A. Chan, R. M. Haley, M. A. Najar, D. Gonzalez-Martinez, L. J. Bugaj, G. M. Burslem, M. J. Mitchell, A. Tsourkas, Lipid-mediated intracellular delivery of recombinant bioPROTACs for the rapid degradation of undruggable proteins. *Nat. Commun.* **15**, 5808 (2024).

54. E. Rohner, R. Yang, K. S. Foo, A. Goedel, K. R. Chien, Unlocking the promise of mRNA therapeutics. *Nat. Biotechnol.* **40**, 1586–1600 (2022).

55. B. Dreier, A. Honegger, C. Hess, G. Nagy-Davidescu, P. R. E. Mittl, M. G. Grütter, N. Belousova, G. Mikheeva, V. Krasnykh, A. Plückthun, Development of a generic adenovirus delivery system based on structure-guided design of bispecific trimeric DARPin adapters. *Proc. Natl. Acad. Sci. U.S.A.* **110**, E869–E877 (2013).

56. T. Sharifnia, M. J. Wawer, A. Goodale, Y. Lee, M. Kazachkova, J. M. Dempster, S. Muller, J. Levy, D. M. Freed, J. Sommer, J. Kalfon, F. Vazquez, W. C. Hahn, D. E. Root, P. A. Clemons, S. L. Schreiber, Mapping the landscape of genetic dependencies in chordoma. *Nat. Commun.* **14**, 1933 (2023).

57. J. Hang, H. Ouyang, F. Wei, Q. Zhong, W. Yuan, L. Jiang, Z. Liu, Proteomics and phosphoproteomics of chordoma biopsies reveal alterations in multiple pathways and aberrant kinases activities. *Front. Oncol.* **12**, 941046 (2022).

58. A. Prieur, F. Tirode, P. Cohen, O. Delattre, EWS/FLI-1 silencing and gene profiling of ewing cells reveal downstream oncogenic pathways and a crucial role for repression of insulin-like growth factor binding protein 3. *Mol. Cell. Biol.* **24**, 7275–7283 (2004).

59. J. W. Schoggins, Interferon-stimulated genes: What do they all do? *Annu. Rev. Virol.* **6**, 1–18 (2019).

60. C. Yang, J. H. Schwab, A. J. Schoenfeld, F. J. Hornicek, K. B. Wood, G. P. Nielsen, E. Choy, H. Mankin, Z. Duan, A novel target for treatment of chordoma: Signal transducers and activators of transcription 3. *Mol. Cancer Ther.* **8**, 2597–2605 (2009).

61. C. Yang, F. J. Hornicek, K. B. Wood, J. H. Schwab, E. Choy, H. Mankin, Z. Duan, Blockage of Stat3 with CDDO-me inhibits tumor cell growth in chordoma. *Spine* **35**, 1668–1675 (2010).

62. B. Dreier, A. Plückthun, Rapid selection of high-affinity binders using ribosome display. *Methods Mol. Biol.* **805**, 261–286 (2012).

63. M. A. Kramer, S. K. Wetzel, A. Plückthun, P. R. E. Mittl, M. G. Grütter, Structural determinants for improved stability of designed ankyrin repeat proteins with a redesigned C-capping module. *J. Mol. Biol.* **404**, 381–391 (2010).

64. J. Tosic, G.-J. Kim, M. Pavlovic, C. M. Schröder, S.-L. Mersiowsky, M. Barg, A. Hofherr, S. Probst, M. Köttgen, L. Hein, S. J. Arnold, Eomes and *Brachyury* control pluripotency exit and germ-layer segregation by changing the chromatin state. *Nat. Cell Biol.* **21**, 1518–1531 (2019).

65. X. Zhang, A. H. Smits, G. B. van Tilburg, H. Ovaa, W. Huber, M. Vermeulen, Proteome-wide identification of ubiquitin interactions using UbIA-MS. *Nat. Protoc.* **13**, 530–550 (2018).

66. W. Huber, A. von Heydebreck, H. Sültmann, A. Poustka, M. Vingron, Variance stabilization applied to microarray data calibration and to the quantification of differential expression. *Bioinformatics* **18**, S96–S104 (2002).

67. W. E. Johnson, C. Li, A. Rabinovic, Adjusting batch effects in microarray expression data using empirical Bayes methods. *Biostatistics* **8**, 118–127 (2007).

68. M. E. Ritchie, B. Phipson, D. Wu, Y. Hu, C. W. Law, W. Shi, G. K. Smyth, *limma* powers differential expression analyses for RNA-sequencing and microarray studies. *Nucleic Acids Res.* **43**, e47–e47 (2015).

69. K. J. Livak, T. D. Schmittgen, Analysis of relative gene expression data using real-time quantitative PCR and the 2−ΔΔCT method. *Methods* **25**, 402–408 (2001).

70. M. I. Love, W. Huber, S. Anders, Moderated estimation of fold change and dispersion for RNA-seq data with DESeq2. *Genome Biol.* **15**, 550 (2014).

71. N. Ignatiadis, B. Klaus, J. B. Zaugg, W. Huber, Data-driven hypothesis weighting increases detection power in genome-scale multiple testing. *Nat. Methods* **13**, 577–580 (2016).

72. M. Stephens, False discovery rates: A new deal. *Biostatistics* **18**, 275–294 (2017).

73. G. Korotkevich, V. Sukhov, N. Budin, B. Shpak, M. N. Artyomov, A. Sergushichev, Fast gene set enrichment analysis. bioRxiv 060012 [Preprint] (2021). https://doi.org/10.1101/060012.

74. A. Liberzon, C. Birger, H. Thorvaldsdóttir, M. Ghandi, J. P. Mesirov, P. Tamayo, The molecular signatures database hallmark gene set collection. *Cell Syst.* **1**, 417–425 (2015).

75. A. Lachmann, D. Torre, A. B. Keenan, K. M. Jagodnik, H. J. Lee, L. Wang, M. C. Silverstein, A. Ma'ayan, Massive mining of publicly available RNA-seq data from human and mouse. *Nat. Commun.* **9**, 1366 (2018).

76. Z. Gu, D. Hübschmann, simplifyEnrichment: A Bioconductor package for clustering and visualizing functional enrichment results. *Genom. Proteom. Bioinform.* **21**, 190–202 (2023).

77. S. Hänzelmann, R. Castelo, J. Guinney, GSVA: Gene set variation analysis for microarray and RNA-seq data. *BMC Bioinform.* **14**, 7 (2013).

78. J. Schöpf, S. Uhrig, C. E. Heilig, K.-S. Lee, T. Walther, A. Carazzato, A. M. Dobberkau, D. Weichenhan, C. Plass, M. Hartmann, G. D. Diwan, Z. I. Carrero, C. R. Ball, T. Hohl, T. Kindler, P. Rudolph-Hähnel, D. Helm, M. Schneider, A. Nilsson, I. Øra, R. Imle, A. Banito, R. B. Russell, B. C. Jones, D. B. Lipka, H. Glimm, D. Hübschmann, W. Hartmann, S. Fröhling, C. Scholl, Multi-omic and functional analysis for classification and treatment of sarcomas with FUS-TFCP2 or EWSR1-TFCP2 fusions. *Nat. Commun.* **15**, 51 (2024).

79. P. Chudasama, S. S. Mughal, M. A. Sanders, D. Hübschmann, I. Chung, K. I. Deeg, S.-H. Wong, S. Rabe, M. Hlevnjak, M. Zapatka, A. Ernst, K. Kleinheinz, M. Schlesner, L. Sieverling, B. Klink, E. Schröck, R. M. Hoogenboezem, B. Kasper, C. E. Heilig, G. Egerer, S. Wolf, C. von

Kalle, R. Eils, A. Stenzinger, W. Weichert, H. Glimm, S. Gröschel, H.-G. Kopp, G. Omlor, B. Lehner, S. Bauer, S. Schimmack, A. Ulrich, G. Mechtersheimer, K. Rippe, B. Brors, B. Hutter, M. Renner, P. Hohenberger, C. Scholl, S. Fröhling, Integrative genomic and transcriptomic analysis of leiomyosarcoma. *Nat. Commun.* **9**, 144 (2018).

80. R. Pelossof, L. Fairchild, C.-H. Huang, C. Widmer, V. T. Sreedharan, N. Sinha, D.-Y. Lai, Y. Guan, P. K. Premsrirut, D. F. Tschaharganeh, T. Hoffmann, V. Thapar, Q. Xiang, R. J. Garippa, G. Rätsch, J. Zuber, S. W. Lowe, C. S. Leslie, C. Fellmann, Prediction of potent shRNAs with a sequential classification algorithm. *Nat. Biotechnol.* **35**, 350–353 (2017).

81. M. Trautmann, Y. Cheng, P. Jensen, N. Azoitei, I. Brunner, J. Hüllein, M. Slabicki, I. Isfort, M. Cyra, R. Berthold, E. Wardelmann, S. Huss, B. Altvater, C. Rossig, S. Hafner, T. Simmet, A. Ståhlberg, P. Åman, T. Zenz, U. Lange, T. Kindler, C. Scholl, W. Hartmann, S. Fröhling, Requirement for YAP1 signaling in myxoid liposarcoma. *EMBO Mol. Med.* **11**, e9889 (2019).

82. A. Shevchenko, H. Tomas, J. Havli, J. V. Olsen, M. Mann, In-gel digestion for mass spectrometric characterization of proteins and proteomes. *Nat. Protoc.* **1**, 2856–2860 (2006).

83. S. Tyanova, T. Temu, J. Cox, The MaxQuant computational platform for mass spectrometry-based shotgun proteomics. *Nat. Protoc.* **11**, 2301–2319 (2016).

84. B. Schwanhäusser, D. Busse, N. Li, G. Dittmar, J. Schuchhardt, J. Wolf, W. Chen, M. Selbach, Global quantification of mammalian gene expression control. *Nature* **473**, 337–342 (2011).

85. T. Müller, M. Kalxdorf, R. Longuespée, D. N. Kazdal, A. Stenzinger, J. Krijgsveld, Automated sample preparation with SP3 for low-input clinical proteomics. *Mol. Syst. Biol.* **16**, e9111 (2020).

86. J. Jumper, R. Evans, A. Pritzel, T. Green, M. Figurnov, O. Ronneberger, K. Tunyasuvunakool, R. Bates, A. Žídek, A. Potapenko, A. Bridgland, C. Meyer, S. A. A. Kohl, A. J. Ballard, A. Cowie, B. Romera-Paredes, S. Nikolov, R. Jain, J. Adler, T. Back, S. Petersen, D. Reiman, E. Clancy, M. Zielinski, M. Steinegger, M. Pacholska, T. Berghammer, S. Bodenstein, D. Silver, O. Vinyals, A. W. Senior, K. Kavukcuoglu, P. Kohli, D. Hassabis, Highly accurate protein structure prediction with AlphaFold. *Nature* **596**, 583–589 (2021).

87. M. A. Cianfrocco, M. Wong, C. Youn, R. Wagner, A. E. Leschziner, "COSMIC^2: A science gateway for cryo-electron microscopy structure determination," in *PEARC '17: Practice and Experience in Advanced Research Computing 2017: Sustainability, Success and Impact* (Association for Computing Machinery, 2017), pp. 9–13.

88. A. Kohl, H. K. Binz, P. Forrer, M. T. Stumpp, A. Plückthun, M. G. Grütter, Designed to be stable: Crystal structure of a consensus ankyrin repeat protein. *Proc. Natl. Acad. Sci. U.S.A.* **100**, 1700–1705 (2003).

89. F. Radom, E. Paci, A. Plückthun, Computational modeling of designed ankyrin repeat protein complexes with their targets. *J. Mol. Biol.* **431**, 2852–2868 (2019).

90. A. Dobin, C. A. Davis, F. Schlesinger, J. Drenkow, C. Zaleski, S. Jha, P. Batut, M. Chaisson, T. R. Gingeras, STAR: Ultrafast universal RNA-seq aligner. *Bioinformatics* **29**, 15–21 (2013).

91. A. Tarasov, A. J. Vilella, E. Cuppen, I. J. Nijman, P. Prins, Sambamba: Fast processing of NGS alignment formats. *Bioinformatics* **31**, 2032–2034 (2015).

92. H. Li, B. Handsaker, A. Wysoker, T. Fennell, J. Ruan, N. Homer, G. Marth, G. Abecasis, R. Durbin, 1000 Genome Project Data Processing Subgroup, The sequence alignment/map format and SAMtools. *Bioinformatics* **25**, 2078–2079 (2009).

93. D. S. DeLuca, J. Z. Levin, A. Sivachenko, T. Fennell, M.-D. Nazaire, C. Williams, M. Reich, W. Winckler, G. Getz, RNA-SeQC: RNA-seq metrics for quality control and process optimization. *Bioinformatics* **28**, 1530–1532 (2012).

94. Y. Liao, G. K. Smyth, W. Shi, featureCounts: An efficient general purpose program for assigning sequence reads to genomic features. *Bioinformatics* **30**, 923–930 (2014).

95. J. F. Dekkers, M. Alieva, L. M. Wellens, H. C. R. Ariese, P. R. Jamieson, A. M. Vonk, G. D. Amatngalim, H. Hu, K. C. Oost, H. J. G. Snippert, J. M. Beekman, E. J. Wehrens, J. E. Visvader, H. Clevers, A. C. Rios, High-resolution 3D imaging of fixed and cleared organoids. *Nat. Protoc.* **14**, 1756–1771 (2019).

96. B. Paré, L. T. Deschênes, R. Pouliot, N. Dupré, F. Gros-Louis, An optimized approach to recover secreted proteins from fibroblast conditioned-media for secretomic analysis. *Front. Cell. Neurosci.* **10**, 70 (2016).
